# Genomic imprinting, methylation and parent-of-origin effects in reciprocal hybrid endosperm of castor bean

**DOI:** 10.1093/nar/gku375

**Published:** 2014-05-05

**Authors:** Wei Xu, Mengyuan Dai, Fei Li, Aizhong Liu

**Affiliations:** 1 Kunming Institute of Botany, Chinese Academy of Sciences, 132 Lanhei Road, Kunming 650201, China; 2 Key Laboratory of Tropical Plant Resource and Sustainable Use, Xishuangbanna Tropical Botanical Garden, Chinese Academy of Sciences, 88 Xuefu Road, Kunming 650223, China; 3 Graduate University of the Chinese Academy of Sciences, Beijing, China

## Abstract

Genomic imprinting often results in parent-of-origin specific differential expression of maternally and paternally inherited alleles. In plants, the triploid endosperm is where gene imprinting occurs most often, but aside from studies on *Arabidopsis*, little is known about gene imprinting in dicotyledons. In this study, we inspected genomic imprinting in castor bean (*Ricinus communis*) endosperm, which persists throughout seed development. After mapping out the polymorphic SNP loci between accessions ZB306 and ZB107, we generated deep sequencing RNA profiles of F1 hybrid seeds derived from reciprocal crosses. Using polymorphic SNP sites to quantify allele-specific expression levels, we identified 209 genes in reciprocal endosperms with potential parent-of-origin specific expression, including 200 maternally expressed genes and 9 paternally expressed genes. In total, 57 of the imprinted genes were validated via reverse transcriptase-polymerase chain reaction sequencing, and analysis of the genomic DNA methylation distribution between embryo and endosperm tissues showed significant hypomethylation in the endosperm and an enrichment of differentially methylated regions around the identified genes. Curiously, the expression of the imprinted genes was not tightly linked to DNA methylation. These results largely extended gene imprinting information existing in plants, providing potential directions for further research in gene imprinting.

## INTRODUCTION

Genomic imprinting is a typical epigenetic phenomenon that mainly manifests itself in the placenta of mammals and the endosperm of flowering plants (1,2). Countering the standard Mendelian understanding of genetics, genomic imprinting leads to parent-of-origin specific differential expression of both maternally and paternally inherited alleles (3,4). In plants, genomic imprinting is predominantly found in endosperm tissues derived from the unique form of double-fertilization present in flowering plants, wherein one sperm fertilizes a haploid egg cell, generating a diploid embryo, and another sperm fertilizes this diploid central cell, resulting in a triploid endosperm. While the resulting endosperm is well-known for both its nutritional and necessity in ensuring viability, no wonder, it makes up 80% of a given seed, there are some intriguing biological aspects of gene imprining that occurs only during endosperm development that remains largely unknown. Our typical understanding of imprinting is that it arises as a by-product of a silencing mechanism targeting invading foreign DNA (5–7). Imprinting has likewise been attributed to conflicts over the distribution of resources from mother to offspring (8). There is also other evidence showing that imprinted genes affect the demand and supply of nutrients in both the mammalian placenta and during plant endosperm development (4,9). If correct, this last possibility may imply that gene imprinting could actually be an essential element of successful reproduction that manifests itself in both flowering plants and mammals (9–11).

Both DNA methylation and chromatin modifications have been widely proposed as the major underlying molecular mechanisms that modulate the expression of imprinted genes; a hypothesis that fits well with the observed genome-wide hypomethylation of the maternal genome in endosperm tissue in *Arabidopsis* (5,12), maize (13) and rice (14). Several current lines of evidence suggest that many regions of genomic endosperm hypomethylation correspond to fragments of transposable elements (TEs) (5,12,14), implying that DNA methylation may be a driving force in establishing gene imprinting. Recent studies have further demonstrated that the DNA glycosylase DEMETER (DME) demethylates maternal alleles of imprinted genes in the central cell prior to fertilization, resulting in lower observed DNA methylation levels in endosperm as compared with either embryos or vegetative tissues of *Arabidopsis* (5,12). Parental differences in DNA methylation in plants have also been found in different imprinted genes identified, such as FIS2 (15), FWA (16), ZmFie1 (17), ZmFie2 (18) and Mez1 (19). However, observations on genomic DNA methylation revealed that the presence of DNA methylation apparently has, at least for most of the currently identified imprinted genes, comparatively little effect on gene expression, with only a subset of DNA methylation likely impacting gene expression (5). Alongside the suggestive evidence on the role DNA methylation in gene imprinting, there is also evidence that chromatin modifications mediated by polycomb group proteins contribute to imprinted regulation (20,21).

While both DNA methylation and chromatin modification offer some interesting possibilities for the mechanisms underlying gene imprinting, the existing information is largely speculative. The known or predicted imprinted genes present in plants appear to be involved in a variety of functions, but the biological bases of which are uncertain (22). To date, the understandings on the origin and evolution of imprinted genes in plants has been restricted by our relatively sparse knowledge of imprinted loci, and consequently the identification and characterization of imprinted genes is restricted to a limited number of plant species, including *Arabidopsis* (6,7), rice (23) and maize (24,25). In most dicots, including *Arabidopsis*, cellularized endosperms do not undergo significant proliferative growth and are instead absorbed by the developing embryo during seed development (26). By contrast, in some species of dicots (such as castor bean, jatropha and coffee), endosperms undergo an intense period of cell division immediately following cellularization, followed by differentiation and specialization that act to form a nutritive tissue for supporting the embryo and seedling during germination (26,27). Comparisons of imprinted genes found in such developmentally distinct seed types of different evolutionary origin would likely provide much greater insight into the conservation, role and importance of imprinted genes in seed development, and may offer some clues into understanding the nature of gene imprinting.

Given the state of research on genomic imprinting among dicots, identifying and separating imprinted genes from reciprocal endosperms would be greatly beneficial for dissecting the genetic mechanism underlying gene imprinting in plants. To that end, testing the genome-wide differential expression of alleles with single nucleotide polymorphisms (SNPs) identified from paternal or maternal origin in endosperm tissues provides an excellent model to identify and characterize imprinted loci and parent-of-origin effects. Castor bean (*Ricinus communis*, Euphorbiaceae, 2n = 20), unlike *Arabidopsis*, an important non-edible oilseed crop which has not been studied in such a fashion, may provide an excellent system for identifying imprinted loci and characterizing parent-of-origin effects of imprinted genes in endosperm development among dicots, because though castor bean endosperm is relatively large and persists throughout seed development, its endosperm can be easily separated from its embryo, and in doing so avoid tissue contamination of embryo and endosperm during sampling. Likewise, the castor endosperm tissues accumulate a variety of rich and unique storage materials, such as ricinoleic acid, widely is used in aviation oil, lubricants and as feedstock for biodiesel production (28), as well as the ricin protein, a poisonous compound often used as bio-weapon (29).

Due to both the myriad uses of the castor bean and their importance to a number of fields, dissecting the genetic mechanism of endosperm development would not only provide useful information in both understanding the genetic basis of storage material accumulation in seed filling, but offer novel approaches to more effectively utilizing the castor bean. More importantly, the castor bean is an excellent test case for such an experiment, as its genome has been well annotated, providing a great deal of information on genomic organization and TEs (29). In this study, we used the castor reference genome to perform a deep mRNA-seq and identifying allele SNPs between two elite inbred lines ZB306 (with small seed size) and ZB107 (with large seed size). Using ZB306 and ZB107 as parents, we constructed reciprocal endosperm mRNA-seq libraries. According to the differential expression of alleles in reciprocal seeds we identified 209 imprinted genes with parent-of-origin specific expression in the endosperm. Further genomic DNA methylation analyses showed that gene imprinting was closely associated with DNA methylation, greatly extending the available genomic imprinting information in eudicot plants and providing opportunities for understanding the mechanisms that drive gene imprinting and seed development in plants.

## MATERIALS AND METHODS

### Samples collection and mRNA-seq

In this study, we used castor bean var. ZB306 and ZB107 elite inbred lines, because they are each important progenitors for castor hybrid breeding. Both sets of seeds (kindly provided by Zibo Academy of Agricultural Sciences, Shandong, China) were planted in a glasshouse (13 h day/11 h night, 28°C/22°C) at Kunming Institute of Botany (Kunming, Yunnan, China) between April and October 2012. To obtain high-quality SNPs between ZB306 and ZB107, we gathered developing seed tissues at several stages: early (ca. 15 days after self-fertilization, DAF), middle (ca. 30 DAF) and late (ca. 45 DAF) stages of seed development. The samples for each variety at each stage were harvested and pooled for later total RNA extraction and deep high-throughput RNA sequencing. For the reciprocal crosses, designed female partners were emasculated and hand-pollinated after the female flowers were mature and the pollens were appropriately developed. Developing seeds from reciprocal crosses nearly attained their maximal volume at 35 DAF, at which time endosperm tissues from reciprocal crosses of F1 (ZB107×ZB306) and F1′ (ZB306×ZB107) were collected and the seed coat and embryo were dissected. A small amount of endosperm tissues rooted in the embryo was removed and watered using sterile water to avoid tissue contamination.

To create three biological replicates for determining gene expression, endosperm tissues collected from three seeds were pooled and immediately frozen in liquid nitrogen prior to total RNA extraction. Total RNA was isolated using TRIzol (Invitrogen, Carlsbad, CA, USA) and purified using an RNeasy Mini Kit (Qiagen, Valencia, CA, USA) following the manufacturer's protocols. The quality of total RNA samples was checked using a NanoDrop Spectrometer (ND-1000 Spectrophotometer, Peqlab) as well as agarose gel electrophoresis. Approximately 35 μg of total RNA from parental seeds and endosperm tissues for each reciprocal cross were sent to BGI-Shenzhen for transcriptome library preparation, and were then paired-end -90 bp sequenced using an Illumina HiSeq 2000 system.

### SNP detection and identification of SNP-associated reads

Raw sequencing data from each library were preprocessed to filter out clipped adapter sequences, contaminated sequences, low-quality reads and reads that mapped to more than one position in the reference genome (http://castorbean.jcvi.org/index.php). For parental transcriptomic data, clean reads were synchronously aligned to the castor bean reference genome for SNP identification between ZB107/ZB306 using SOAPsnp-v1.03 (30). To reduce the detection rate of false-positive SNPs, SNP detection was performed using the following stringent criteria: (i) the sufficient bases quality (*Q* values > 20); (ii) at least five reads coverage across SNP site; (iii) removal of the SNPs located in reads ends and (iv) exclusion of heterozygous sites of each sample. The remaining SNPs between ZB107/ZB306 defined as being high­-quality were used for subsequent analysis.

According the method used by Wolff *et al.* (7), we extracted a 191nt genomic flanking sequence around the ZB107/ZB306 SNP (SNP position ±90 bp) from the reference genome, and then the nucleotide in the SNP was replaced by the ZB107 and ZB306 variant named as ZB107 and ZB306 windows, respectively, with a Perl script. Clean reads of each reciprocal cross were mapped to ZB107 and ZB306 windows, respectively, using SOAP3 (31) with the following criterion: (i) both forward and reverse complementary orientation, (ii) up to four mismatches, (iii) the whole read must map and (iv) they generated maximal substring matches. Next, we counted the read number of ZB107 and ZB306 alleles of each reciprocal crosses that could be used to perform differential allele expression analysis.

### Detecting imprinting SNPs

For each locus in the endosperm tissue, we determined a joint *P*-value for the null hypothesis (no imprinting) using a binomial one-sided test based on Wolff *et al.*'s method (7). First, two *P*-values (*p*_*1*_, *p_2_*) were calculated for the probabilities of deviation from the expected 2m:1p ratio, toward either larger maternal expression or larger paternal expression under the null hypothesis of an unbiased 2m:1p expression in the endosperm. *p_1_* was defined as the ZB107 portion for a given locus in ZB107×ZB306 crosses, and *p_2_* as the portion of ZB306 in ZB107×ZB306 crosses. Next, the two *P*-values for maternal expression from the two reciprocal crosses (*p_1_*, *p_2_*) were summarized in a joint *P*-value based on the distribution of the second-order statistic by calculating *P* = max (*p_1_*, *p_2_*)∧2. Joint *P*-values for paternal expression were analogously calculated. Collectively, joint *P*-values either describing reciprocal maternal expression or reciprocal paternal expression were sorted in ascending order (from significant to insignificant), and for each joint *P*-value the false-discovery rate (FDR) was calculated as FDR = P*n/*i*, where *n* was the overall number of joint *P*-values and *i* was the rank of a given *P*-value. Genes with a FDR ≤ 0.05 were selected as maternally or paternally expressed genes , and these candidates were further filtered in endosperm to identify imprinted genes for which at least 90% of reads were derived from one parent in both reciprocal hybrids.

### Validation of imprinted gene and expression analysis

Imprinted loci were validated using independently prepared mRNAs from reciprocal hybrid endosperms between ZB107 and ZB306. Primers used for allele-specific expression analysis of selected genes are listed in Supplementary Table S1. The amplified products were analyzed on agarose gels and then sequenced. Additionally, we collected the endosperms at 25, 30 and 50 DAF for developmental analyses. Other tissues from the leaf, stem, root, male and female flower, embryo and endosperm were harvested from the parent for testing tissue specificity.

### Functional characterization of imprinted features

TEs have been considered as a driving force for the evolution of imprinting gene expression, so we investigated the number of TEs around the imprinted genes within 4 kb flanking regions of a given imprinted gene. Annotated TEs were downloaded from castor bean genome database (ftp://ftp.jcvi.org/pub/data/castorbean/temp/). Gene annotation of imprinted gene was carried out using Blast2GO program, while Gene Ontology (GO) enrichment analysis was performed with WEGO (32). We also investigated homologous sequences of castor bean imprinted genes in *Arabidopsis*, rice and maize as well. Candidate imprinted genes previously reported in other species were downloaded and aligned to castor bean transcript database (http://castorbean.jcvi.org/index.php) using Basic Local Alignment Search Tool. The top three hits with significant *E* value (*E* ≤ 0.001) and high similarity score (Identity > 60%) corresponding to castor bean imprinted genes were selected for further study.

### Bisulfite sequencing

Bisulfite sequencing was performed as described previously (12). Briefly, genomic DNA (about 3 μg) from five replicates of 35-DAF endosperm and embryo from the cross ZB107×ZB306 was isolated using the Plant DNeasy Minikit (Qiagen, Valencia, CA, USA), and then was shared by sonication to fragments of 300–500 bp. Custom Illumina adapters were ligated following the manufacturer's protocols. Fragments with adapters were bisulfite converted twice by sodium bisulfite using EpiTech Bisulfite Kits (Qiagen, Valencia, CA, USA) and then amplified by 18 cycles of polymerase chain reaction (PCR) using Pfu DNA polymerase (TaKaRa, Dalian, China). Following this process, the libraries were sequenced at BGI (Shenzhen), generating paired-end 90 bp reads from each library.

### Differentially methylated regions (DMRs) analyses

Similar to Hsieh *et al.* (12), the data we obtained were aligned to the castor bean genomic scaffolds using the BSMAP software, allowing up to two mismatches per read. Reads that mapped to more than one position were removed. Next, we used Perl scripts to count the methylation level of each cytosine based on aligned reads. To identify significant CpG methylation differences between endosperm and embryo tissue, we selected the regions corresponding to the following stringent criteria: (i) more than five methylated CpG sites at least one sample; (ii) more than 10 reads for each cytosine; (iii) segment length >40 bp, with the distance between adjacent methylated sites <200 bp; (iv) mean difference for fractional CpG methylation of at least 0.5 and (v) Pearson's *χ*^2^ test value of *P* ≤ 0.05. In the present study, the DMRs overlapping at adjacent 2 kb (upstream or downstream) or gene body regions of imprinted genes were chosen and turned into candidate DMRs for further study.

For the identification of allele-specific DMRs, we followed the procedures of Zhang *et al.* (25), extracting parent-of-origin reads with a Perl script according to the SNP loci between ZB107 and ZB306 accessions to determine the methylation level of each allele. We calculated an allelic methylation difference score in overlapping 200 bp segments at adjacent 2 kb and gene body regions of imprinted genes. These differential regions contained at least five informative sequenced methylcytosine and more than five reads coverage. Moreover, the methylation level of one allele should be less than 40% while the other allele more than 70%.

## RESULTS

### Identification of imprinted loci in endosperm

To discriminate the parental origin of allelic expression in hybrids, we conducted a deep high-throughput RNA sequencing for seed libraries of two castor bean varieties ZB107 and ZB306 to discover SNPs between the two varieties, yielding some 50 million reads. Initially, 10,164 SNP sites with at least one read support were identified between ZB107 and ZB306, but after filtering ambiguous loci with low abundance reads, 8,540 high-quality SNPs finally were retained for identifying and measuring allelic expression (Supplementary Table S2). We next performed reciprocal crosses of two castor bean accessions ZB107 and ZB306, and obtained the hybrid F1 (ZB306×ZB107) and F1′ (ZB107×ZB306) seeds. Anatomical observations indicated that both hybrid F1 and F1′ seeds were normally developed, and that their embryos and endosperms were well developed (see Supplementary Figure S1). Interestingly though, physical observation showed that the mature hybrid seeds morphologically exhibited strong similarities to their maternal parents (see Supplementary Figure S1), implying some potentially unusual genetic regulatory mechanisms and a strong maternal effects in castor bean.

**Figure 1.**
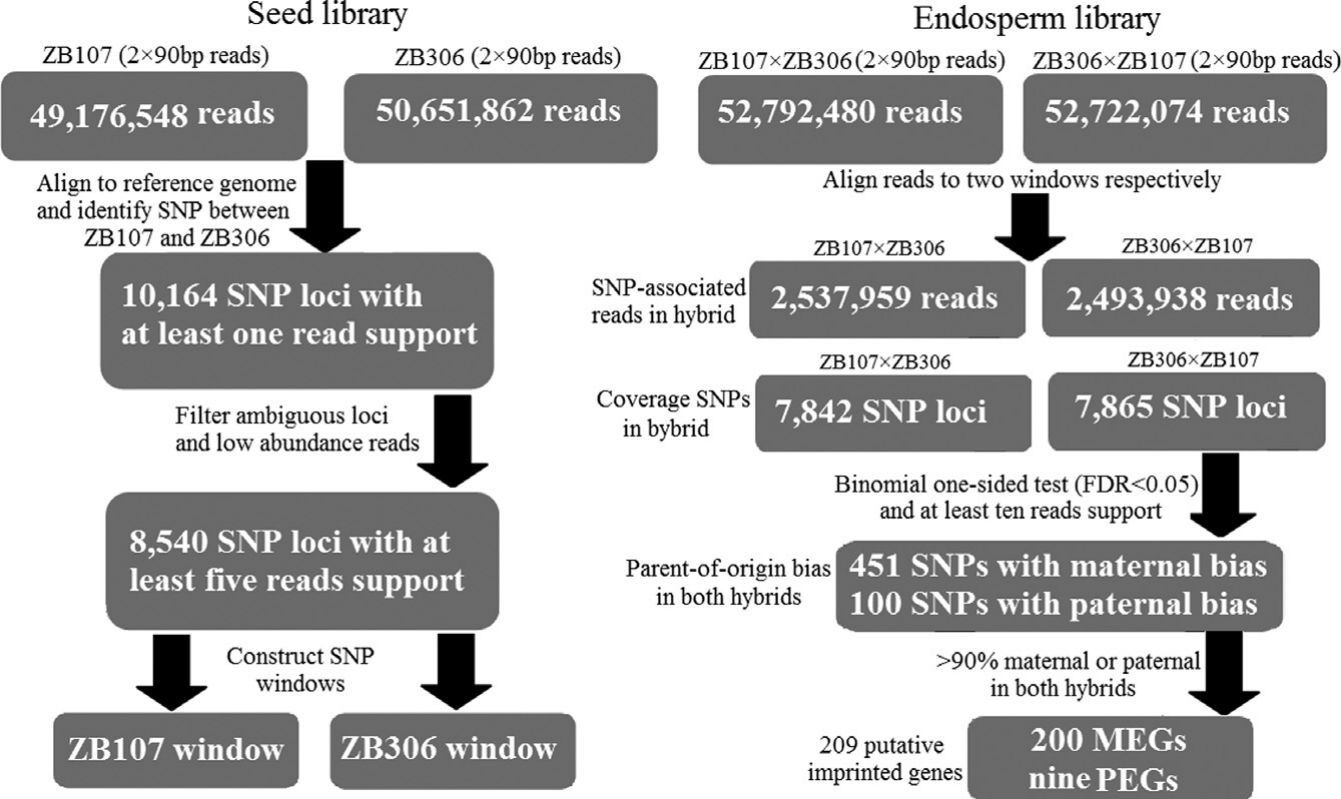
Flow chart for identification of imprinted genes in castor bean endosperm.

We next collected the developing endosperm tissues (ca. 35 DAF) from reciprocal hybrid seeds and constructed two endosperm tissue libraries before performing deep high-throughput mRNA-seq (see Materials and Methods for details), yielding a total of 53 million 90-bp pair-end reads (∼4.7 Gb) with ∼68× transcriptome depth. Clean reads were then aligned to the ZB107 and ZB306 windows to allow for calling SNP coverage reads and characterizing the allelic expression of a given hybrid endosperm (see Figure 1). Totally, about 7,800 SNPs with at least one read could be assigned to a specific allele in two reciprocal hybrid endosperms (Figure 1), of which there were 5,279 high-quality SNP loci containing more than 10× read coverage. These high-quality SNP loci allowed us to accurately assess allele-specific expression in reciprocal hybrid endosperms.

**Figure 2.**
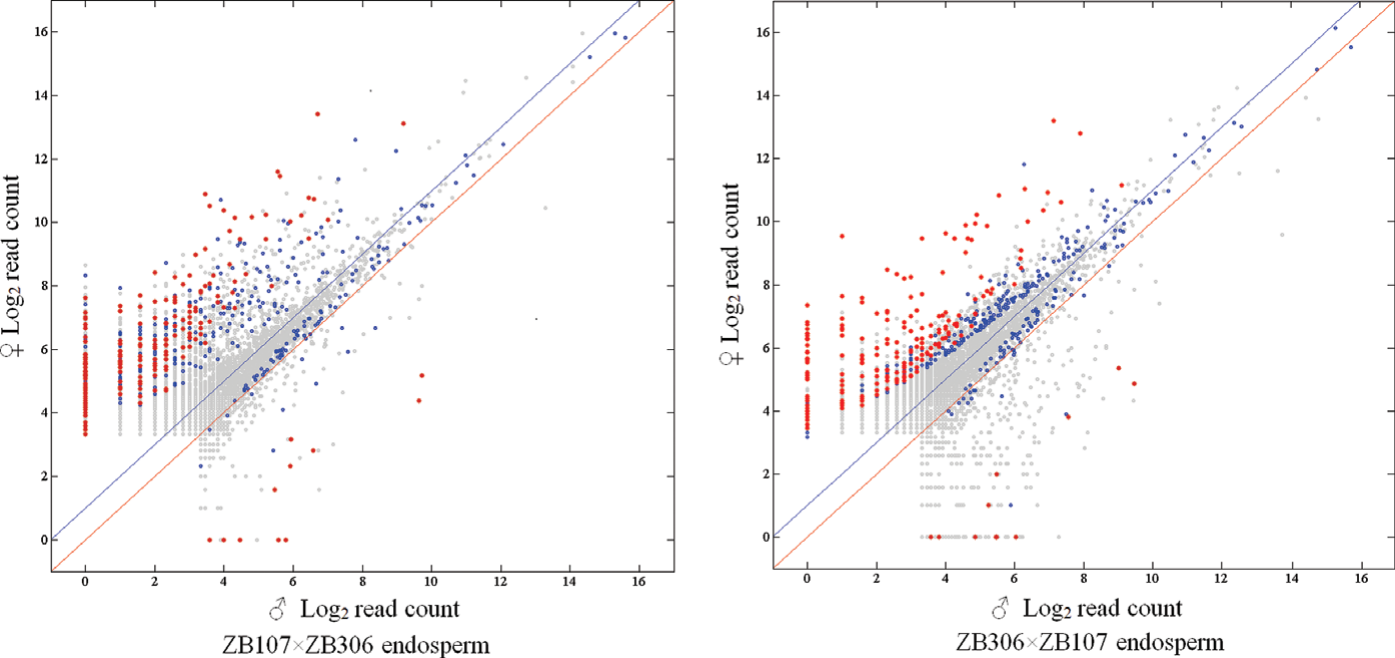
Log2 normalized read counts for all SNP loci for the endosperms. All color dots (including the blue and red) represent the imprinted loci generated from the first selection (FDR < 0.05). Only the red dots represent the imprinted loci sorted out from the second selection with >90% read percentage bias in both reciprocal cross endosperms. The red and blue lines denote the 1:1 and 2:1 ratios (maternal to paternal), respectively.

Generally in a triploid endosperm, when two alleles are equivalently expressed a 2:1 allelic-specific expression ratio from endosperm tissues can be expected. Here, the expression of the major SNP sites tested (∼90%) were proportional to the genomic contribution in both reciprocal hybrids (Figure 2). However, a small proportion of SNP sites (∼10%) exhibited parent-of-origin differences in the expression of maternal and paternal alleles. Among these, 451 SNP sites exhibited maternally biased expression (see Figure 2 and Supplementary Table S3) another 100 SNPs displayed paternally biased expression (see Figure 2 and Supplementary Table S4) in reciprocal hybrids (Binomial one-sided test, FDR < 0.05). These differentially expressed SNPs were considered as putatively imprinted loci.

One potential complication worth considering during our identification of the imprinted loci was the RNA contamination from maternal tissues, such as long-lived RNAs or gametophytic tissue that might mimic gene imprinting. Though we presumed that the 35 DAF endosperm tissues likely contain rare transcripts generated from parental gametes, it is difficult to rule out the interference of potential genes transcripted in seed coat tissues that may have been transported into the endosperm. To avoid a bias toward strongly expressed genes in seed coat, we applied stringent standards, requiring >90% uniparental transcripts in both reciprocal crosses samples, in doing so, we were able to obtain a subset of high-confidence imprinted loci. Under this criterion, we were able to shortlist 225 candidate imprinted loci in castor bean endosperm. Of these, 184 imprinted loci were maternally expressed (covering 178 expressed genes, MEGs; see Supplementary Table S5) and 11 loci were paternally expressed (covering 9 expressed genes, PEGs; see Supplementary Table S6). Interestingly, we noted that most of the imprinted genes identified from our mRNA-seq analysis exhibited partial imprinting (preferential expression of one allele) rather than complete imprinting (strict monoallelic expression). The remaining 30 imprinted loci were maternally expressed and mapped to the intergenic regions within the upstream or downstream of annotated genes on the genome. These may represent extended UTRs (untranslated regions) of annotated genes, novel transcripts or non-coding RNAs, so consequently we realigned the endosperm sequencing reads to castor bean genome sequences and assembled them, yielding a large number of extended regions at 5′ or 3′ end of known genes, and novel transcripts mapped to non-annotated genomic regions that were identified and characterized.

Totally, 22 imprinted loci were located in the 5′ or 3′ UTR of annotated genes, and 8 derived from the novel transcript region with an average length of 532 bp (Supplementary Table S7). These novel transcripts had no complete ORF (open reading frame) or any known homologs in other species. A further search of reported small RNAs data set in castor bean (33) with the sequences of these intergenic loci detected no known match to small non-coding RNAs, and also lacked the conserved hairpin structure of microRNAs. These imprinted non-coding transcripts with relatively greater lengths are likely a class of long non-coding RNAs, but the transcription and imprinting status of these intergenic transcripts remain to be further tested. In summary, collective *in silico* transcriptome analysis of the reciprocal endosperm tissues identified 200 maternally and 9 paternally imprinted genes (see Figure 1), as well as 8 maternally biased non-genic transcripts present in the endosperm.

In addition, it is important to note that a panel of genes exhibited non-imprinted allele-specific expression pattern or subspecies biased expression in our data set. A ZB107 allele might, for example, be either exclusively or more highly expressed than a ZB306 allele, independent of the direction of cross. These subspecies biased expression potentially are particularly interesting, as they may allow for the characterization of expressional genetic regulation in these subspecies. In this study, 61 ZB107 genes and 10 ZB306 genes exhibited >90% uni-parentally biased expression in both reciprocal crosses (Binomial one-sided test, FDR < 0.05). Subsequently, we selected 10 exclusively expressed genes (6 ZB107 genes and 4 ZB306 genes) for experimental confirmation of this supposition, which are listed in Supplementary Table S8.

### Validation of imprinted loci

**Figure 3.**
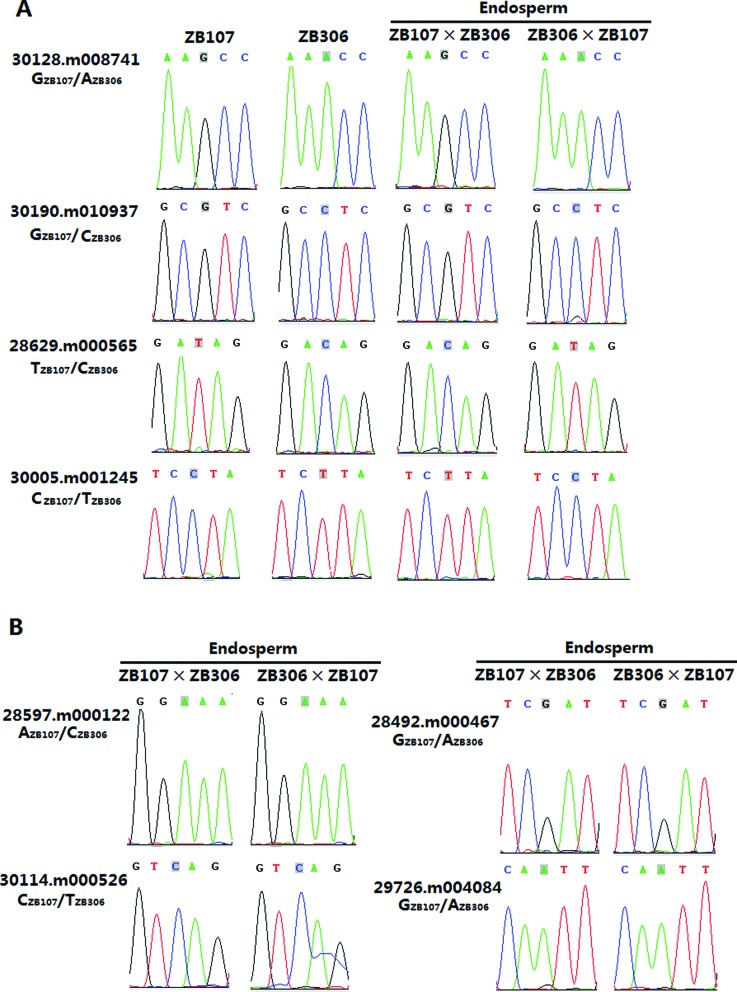
Allele-specific expression analysis detected in castor bean seed. (A) Validation of imprinted genes (MEGs: 30128.m008741, 30190.m010937; PEGs: 28629.m000565, 30005.m001245) by RT-PCR sequencing. (B) Confirmation of genes with non-imprinted allele-specific expression pattern (ZB107: 28597.m000122, 28492.m000467, 30114.m000526; ZB306: 29726.m004084).

For experimental confirmation, we tested the allelic-specific expression patterns observed among the mRNA-seq analysis for a subset of MEGs with 95% or greater bias, and more than 100 reads abundance (see Supplementary Table S5) and all the PEGs. Using reverse transcriptase-PCR (RT-PCR) sequencing analysis of an independent sample of hybrid 35-DAF endosperm tissue, we found 51 of the 58 tested MEGs were predominantly expressed from maternal alleles in reciprocal crosses, whereas 6 of all 9 PEGs tested were preferentially paternally expressed from the maternal alleles in reciprocal crosses (see Figure 3A and Supplementary Figure S2), consistent with mRNA-seq data. Five genes (30174.m008685, 30170.m014148, 29279.m000134, 29816.m000679, 29827.m002618), however, exhibited a significant accession dependency in 35 DAF hybrid endosperm tissue, in which they were exclusively maternally or paternally expressed in one direction of cross but biallelically expressed in the other reciprocal cross (Supplementary Figure S2).

Totally, about 85% of imprinted genes tested were experimentally confirmed and five genes were determined to accession-dependent imprinted genes. We concluded that the majority of the predicted MEGs and PEGs were subject to genomic imprinting and furthermore that the applied mRNA-seq approach used in this study was robust enough to identifying the parent-of-origin specific gene expression. To investigate whether gene imprinting was persistent through the endosperm development, we randomly selected 19 confirmed imprinted genes (17 MEGs and 2 PEGs) for the expression analysis at additional time points earlier at 25 DAF and later at 50 DAF. The results of our analysis showed that most of these maintained their imprinting pattern at the earlier stage of development except that one MEG (29633.m000919) was not detected at this stage, but instead was lost in later stage as expression become biallelic in at least one cross (Supplementary Figure S2B). The accession-dependent imprinted genes confirmed at 35 DAF endosperm exhibited monoallelic expression or complete imprinting at the earlier stage of development, suggesting a dynamic expression of imprinted genes during the development of the castor bean.

Previous studies noted that many imprinted genes are expressed exclusively in endosperm tissue (7,24), so to test whether these validated imprinted genes are expressed exclusively in endosperm tissue in castor bean, we used RT-PCR of various tissues including leaf, stem, root, male and female flower, embryo and endosperm. The results of this experiment showed that 35 of 67 (∼61%) were expressed in different tissues and 22 genes were confined to endosperm-specific or preferential expression (Supplementary Figure S3), implying that most of imprinted genes identified may not be functionally restricted to the endosperm. Additionally, the four non-imprinted allele-specific genes (28597.m000122, 28492.m000467, 30114.m000526 and 29726.m004084) identified from high-through sequencing analyses were experimentally validated by RT-PCR sequencing analyses (Figure 3B).

### Characterization analysis of imprinting genes identified in castor bean

While the existing studies on mammals show clustering of imprinted genes, little evidence shows a similar phenomenon in plant species. Comparisons of the genomic distance between imprinted genes showed that most of imprinted loci were not organized in co-localized clusters, and that only three regions in the genome near to where the two or three imprinted genes were nested, including 29693.m002027/29693.m002028/29693.m002029, 29851.m002492/29851.m002493 and 30128.m008600/30128.m008601 (see Supplementary Figure S4). The average distance between the imprinted genes within the three regions was significantly different from the average distance between randomly selected informative genes. Whether the expression of these mini-clustered imprinted genes is governed by an imprinting control region remains to be seen. GO analysis showed no significant evidence for function enrichment of candidate imprinted genes based on Fisher's exact test. The dominant GO terms related to the imprinted genes are shown in Supplementary Figure S5.

One of the main objectives in this study is to identify the conservation of imprinted genes between monocots and eudicots or different seed types, which would provide a more robust foundation for understanding the evolution and function of imprinted genes. We compared all 209 imprinted protein-coding genes with the known imprinted genes of *Arabidopsis* (6,7,34), rice (23) and maize (24,25,35). Our analysis showed that only 8 imprinted genes were conserved between *Arabidopsis* and castor bean, 5 between rice and castor bean and 12 between maize and castor bean (Supplementary Table S9). Among these conserved genes, only the K domain gene 29765.m000727 (homologous to AT3G08620) encoding a RNA-binding protein showed maternally preferential expression in all four species. The gene 30147.m014492 is common in castor bean, *Arabidopsis* and maize, but it encodes a protein of unknown function (Supplementary Table S9). Collectively, these results showed that the conservation of imprinted genes identified in plants was quite limited.

**Figure 4.**
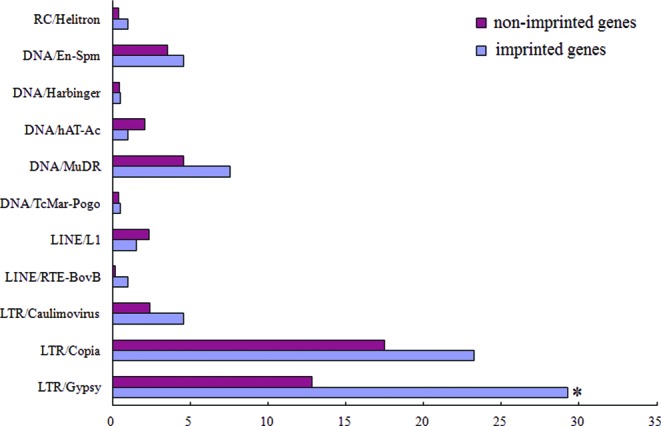
Type of TEs present in imprinted genes (blue bars) in comparison to non-imprinted genes detected in both hybrid endosperms (red bars).

TEs are extensively demethylated during seed development, and are accordingly considered a major driving force being the rise of gene imprinting (5,7,12). As expected, we found significant evidence supporting the enrichment of transposons around the identified imprinted genes identified (Fisher's exact test, *P* = 8.21*e*^−5^). We also tested whether a particular subset of these TEs were enriched in the vicinity near the imprinted genes. Results showed a significant enrichment for the LTR/Gypsy Retrotransposon type (Fisher's exact test, *P* = 2.97*e*^−6^; Figure 4) in the vicinity of castor bean imprinted genes that seemed to be distinctly different from the DNA/MuDR identified in *Arabidopsis* (7) and CACTA TEs in maize (25).

### Endosperm-specific hypomethylation

**Figure 5.**
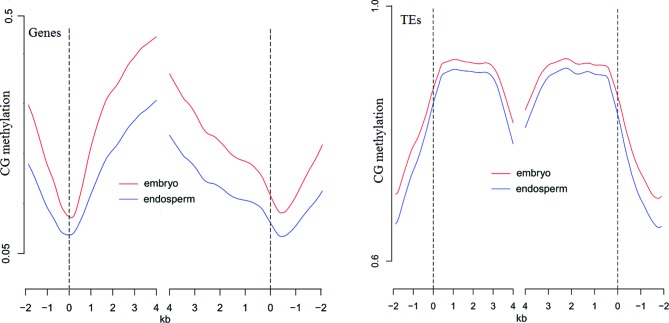
Methylation profiles of CG sequence context in endosperm (blue line) and embryo (red line) tissues from the 35 DAF hybrid of ZB107×ZB306. Castor bean annotated genes and TE were aligned at the 5′ end (Left) or the 3′ end (Right), and the average methylation levels for each 200-bp interval are plotted. The dashed line represents the point of alignment.

**Figure 6.**
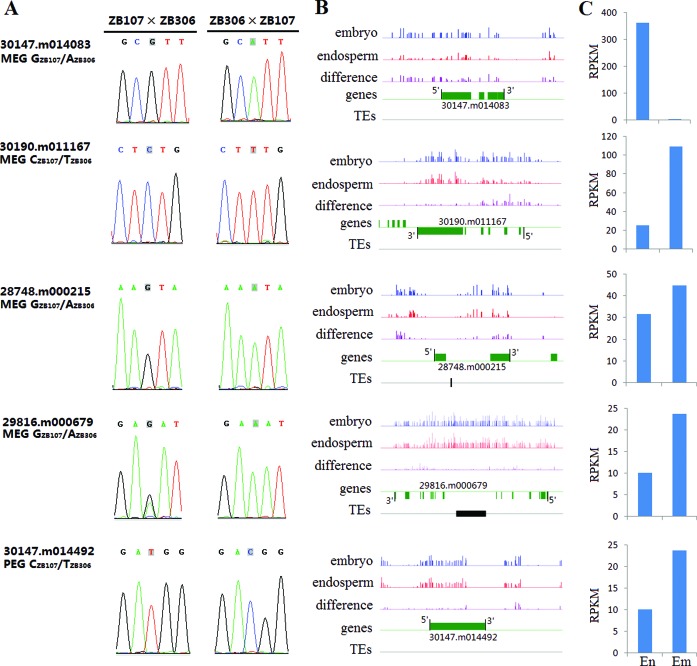
Impact of DNA methylation on the regulation of confirmed imprinted genes. (A) Validation of allele-specific expression in the endosperms form crosses of ZB107×ZB306 and ZB306×ZB107. (B) DNA methylation profiles of indicated imprinted genes in endosperm and embryo. The green boxes represent the gene body regions. The black boxes represent TEs. (C) Expression levels of imprinted genes (RPKM) tested at the 35-DAF endosperm (En) and embryo (Em) tissues.

Several lines of evidence demonstrated that the expression of imprinted genes was intimately tied to the methylation status of allelic-specific DMRs. In the present study, we quantified methylation profiles of the castor bean endosperm and embryo tissues from the 35 DAF hybrid of ZB107×ZB306 using high-throughput bisulfite sequencing (bisulfite treatment converts unmethylated cytosine to uracil). We mapped 10.8 billion bases for embryo and endosperm tissue collectively, corresponding to 30-fold coverage of the castor bean nuclear genome. Testing the CpG methylation level within the 2 kb upstream, gene body or TE and 2 kb downstream between endosperm and embryo libraries, we found a wide hypomethylation in castor bean endosperm compared with embryo tissue (see Figure 5). Meanwhile, we identified 20 imprinted genes (including 19 MEGs and 1 PEG) having a region of endosperm-specific hypomethylation within the body of gene or 2 kb 5′ or 3′ ends (Figure 6 and Supplementary Figure S6). This test showed that gene imprinting was associated with DNA hypomethylation (Fisher's exact test, *P* = 6.6*e*^−6^). Indeed, of the 20 identified imprinted genes, 5 (4 MEGs and 1 PEG) were experimentally confirmed via RT-PCR sequencing (Figure 6A and B). Using this embryonic and endosperm transcriptomic data, expression analysis of the major imprinted genes showed little effect of DNA methylation on gene expression (Figure 6C and Supplementary Figure S6) except for two endosperm preferentially expressed genes (30147.m014083 and 27467.m000162). We further tested allele-speciﬁc methylation patterns in 35 DAF endosperm (see criteria applied in Materials and Methods), which highlighted six imprinted genes with differential methylation region between the parental alleles within upstream, gene body or downstream (Table 1). The six genes all exhibited hypomethylation in maternal alleles and hypermethylation in paternal alleles, while the CpG methylation of four genes was completely derived from the paternal allele (Table 1).

**Table 1.** Summary of identified allele-specific DMRs associated with the imprinted genes in endosperm.

Gene ID	Imprinting Type	DMR Star	DMR End	DMR Length	Methyl% of paternal allele	Methyl% of maternal allele	Genomic region of DMR
27448.m000067	MEG	22494	22836	342	100	33	Gene body
27448.m000067	MEG	24533	24799	266	82	37	Gene body
29669.m000820	MEG	182894	183424	530	71	0	Gene body
29609.m000609	MEG	272404	273161	757	96	0	Gene body
29736.m002024	MEG	190784	191231	447	74	7	Gene body
29453.m000062	MEG	-1947	-1727	220	77	0	Upstream
28738.m000149	MEG	-3406	-3119	287	94	0	Upstream
28738.m000149	MEG	110785	111156	371	96	0	Downstream

## DISCUSSION

### Genomic imprinting in castor bean endosperm

Aside from *Arabidopsis*, there have been no other studies on gene imprinting among dicotyledon endosperms. The present study is the first genome-wide survey of imprinted genes in a dicotyledon species with persistent endosperm throughout seed development, thereby offering a model system for further follow-up studies on endosperm development among dicots. The major aim of our study was to enrich the existing information on genomic imprinting and enlarge the number of identified imprinted genes present in castor bean endosperm. This information may hold the basis for answering several unknown questions on the potential functions, and mechanisms behind gene imprinting in dicots, and perhaps even shed some light into the evolution and biological significance of gene imprinting among plants in general.

Applying high-throughput mRNA-seq analysis with a highly stringent selection criterion, we identified 200 MEGs and 9 PEGs from hybrid endosperms. We noted an overabundance of MEGs and partial imprinting pattern, which may be unexplained by the prevailing view on parental conflict theory. A large number of maternal genes-specific expression may be a consequence of strong maternal-effect phenotype, consistent with our previous observations that the strong similarities between the F1 hybrid seed and the maternal parent. Here, our results largely favored the maternal-offspring coadaptation theory that predicts maternal-specific expression affecting the offspring development and fitness that are vital at the interface of the maternal-offspring interaction (36,37). Potentially, the data supporting this conclusion may help test the hypotheses on how males and females differentially control offspring fate and seed size via the currently enigmatic epigenetic phenomenon of genomic imprinting.

Of the 67 imprinted genes used to examine this possibility, ∼85% were validated using RT-PCR sequencing, suggesting that our strategy used to identify imprinted loci was highly effective. By tracking the imprinting status of several imprinted genes throughout the lifecycle, we found that most of them exhibited a dynamic imprinted expression pattern during endosperm development (see Supplementary Figure S2), consistent with the earlier observations of *Arabidopsis*, rice and maize (6,23,35). By extension, it is quite possible that the imprinted expression in the early stage of endosperm development may be effected by the reprogramming of epigenetic marks following fertilization. Also, it is important to emphasize that our list of imprinted genes is specific to very discrete stages of the castor bean lifecycle, endosperm development and the number of imprinted genes should logically be larger. In addition, five imprinted genes were identified to be accession-dependent. This type of gene imprinting may be an epigenetic natural variation (38–40), and one worth further inquiry.

### Characterization of imprinted genes in the endosperm tissue

The analysis of GO function enrichment for imprinted genes revealed that a majority of MEGs were involved in metabolic process, suggesting these genes are likely associated with specific endospermogenesis processes. At the developmental stages we tested, the cellular endosperm increases dramatically in volume, leading to a rapid increase of seed weight, similar to what occurs during the IV–V stage of seed development (41). Additionally, the specific LTR/gypsy TE family enriched around MEGs was identified in castor bean, which is distinctly different from that observed in *Arabidopsis* (7) and maize (25). This discrepancy may be related to the number and position of specific TEs in the castor bean, because TEs usually vary among different species. Moreover, several imprinted genes located in the syntenic regions in *Arabidopsis* and castor bean are surrounded by totally different sets of TEs. While there is no significant evidence attributing enrichment to TEs, we cannot ignore the possibility that some of these elements may affect a specific subset of imprinted genes.

Suppose gene imprinting functionally plays a similar role in all flowering plants, regardless of the differential seed types, it might be expected that there would be strong conservation for the targets of imprinting (42). Interestingly, we found that the overlap of commonly identified imprinted genes was rather limited between eudicots (castor bean and *Arabidopsis*) as well as between castor bean and maize or rice (monocots). This finding potentially suggests that the differences of gene imprinting between the dicots and monocots may not have resulted from their separate evolutionary history. In particular, poor conservation of imprinted genes was likewise observed in the different developmental stages of seed development or the different accessions within a species (34,35,42). The substantial variation in the targets of imprinting in different species implies the existence of different mechanisms for the origin of imprinted genes and their maintenance in plants (42). The active transposons could be a potential factor in driving gene imprinting for nearby genes, resulting in substantial variation that does not persist over evolutionary time. Our current study revealed that endosperm demethylation occurred primarily at TEs, providing evidence for understanding the potential mechanism that fueled the rise of gene imprinting, at least among castor beans, though potentially on a broader scale. This is at best a logical supposition or extension, and further investigation into the functional differentiation of imprinted genes identified in different plants will likely provide much greater insight into understanding the mechanisms behind gene imprinting in plants.

### Limited regulation of DNA methylation in gene imprinting

DNA methylation has long been regarded as a key player in epigenetic regulation. Differential methylation levels of the maternal and paternal alleles in endosperm tissues have been identified in *Arabidopsis*, rice and maize (5,12–14), and now in castor beans. We noted a significant enrichment of DMRs around the imprinted genes that we identified. Generally, DNA methylation of promoters is considered to inhibit transcriptional initiation, but whether gene expression is significantly linked to DNA methylation remains controversial. In this study, we found that only imprinted genes with endosperm-preferred expression exhibited less methylation in the endosperm than embryo, consistent with previous reports on *Arabidopsis* and maize (5,24), whereas most imprinted genes expression levels were little affected by DNA CpG methylation. Moreover, several MEGs exhibited a dramatic differential methylation level between the two parental alleles (see Table 1). The maternal hypomethylation of MEGs observed in castor bean endosperm implies that there could, potentially, be a DME gene functioning in the castor bean central cell that results in demethylation and gives rise to hypomethylation in the endosperm. The gene 29428.m000327 was identified to be highly homologous to the DME (AT5G04560) of *Arabidopsis*, though its function remained to be determined. Most of the imprinted genes identified display no differential methylation level between the parental alleles, suggesting that DNA methylation being the underlying or driving factor in the rise of gene imprinting may be limited in castor bean. More likely, other factors (such as histone modification) may be the causal mechanisms underlying the rise of gene imprinting in the castor bean. Such possibilities remain to be seen, but it is possible that partial imprinted genes identified might be transcripted in the seed coat and transported into the endosperm embedded in developmental maternal seed coat tissues.

## ACCESSION NUMBERS

The all sequencing raw data used for this study were deposited to the NCBI (National Center for Biotechnology Information) Short Read Archive under accession number SRX485027.

## SUPPLEMENTARY DATA

Supplementary Data are available at NAR Online.

SUPPLEMENTARY DATA
