# Supplementary material for: Genomic imprinting, methylation and parent-of-origin effects in reciprocal hybrid endosperm of castor bean
Source: Nucleic Acids Res. 2014 May 5;42(11):6987–98. doi: 10.1093/nar/gku375 (PMC4066788; doi:10.1093/nar/gku375)
Supplement: SUPPLEMENTARY DATA [file supp_42_11_6987__index.html]

Genomic imprinting, methylation and parent-of-origin effects in reciprocal hybrid endosperm of castor bean — SUPPLEMENTARY DATA 

# Genomic imprinting, methylation and parent-of-origin effects in reciprocal hybrid endosperm of castor bean

## SUPPLEMENTARY DATA

**Files in this Data Supplement:**

- SUPPLEMENTARY DATA
- SUPPLEMENTARY DATA
