# Supplementary material for: Genomic imprinting, methylation and parent-of-origin effects in reciprocal hybrid endosperm of castor bean
Source: Nucleic Acids Res. 2014 May 5;42(11):6987–98. doi: 10.1093/nar/gku375 (PMC4066788; doi:10.1093/nar/gku375)

Supplementary Figure S1. Anatomical analysis of fruit derived from selfed parents (ZB107 and ZB306) and reciprocal crosses (ZB107×ZB306 and ZB306×ZB107). The developing castor bean fruit at indicated days after fertilization (DAF) showing seed (sd) and embryo (em) morphology.

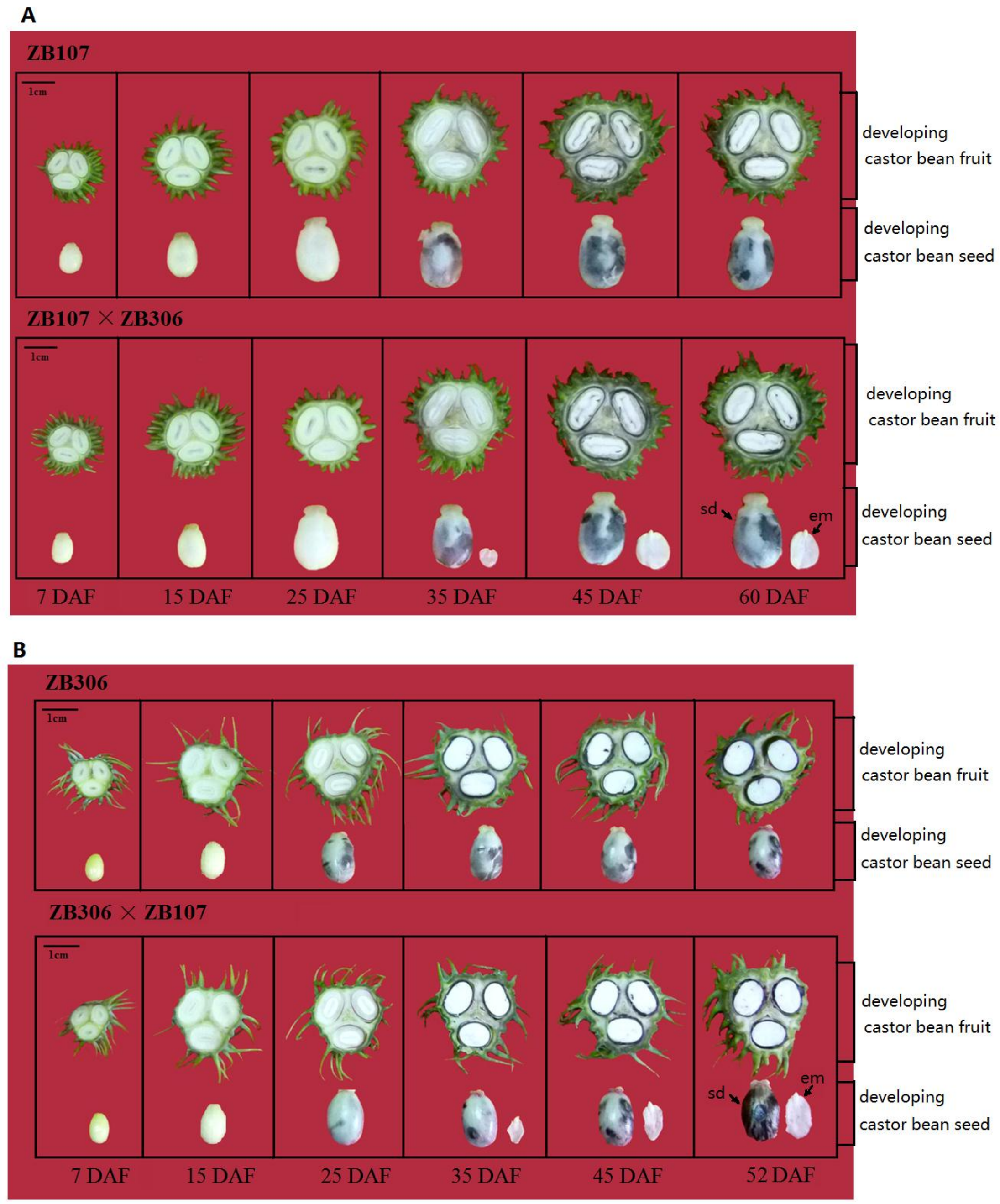

Supplementary Figure S2. Validation of imprinted genes by RT-PCR sequencing at 35 DAF endosperm from reciprocal crosses (A and B) and expression profiles of imprinted genes at different stages of development (B).

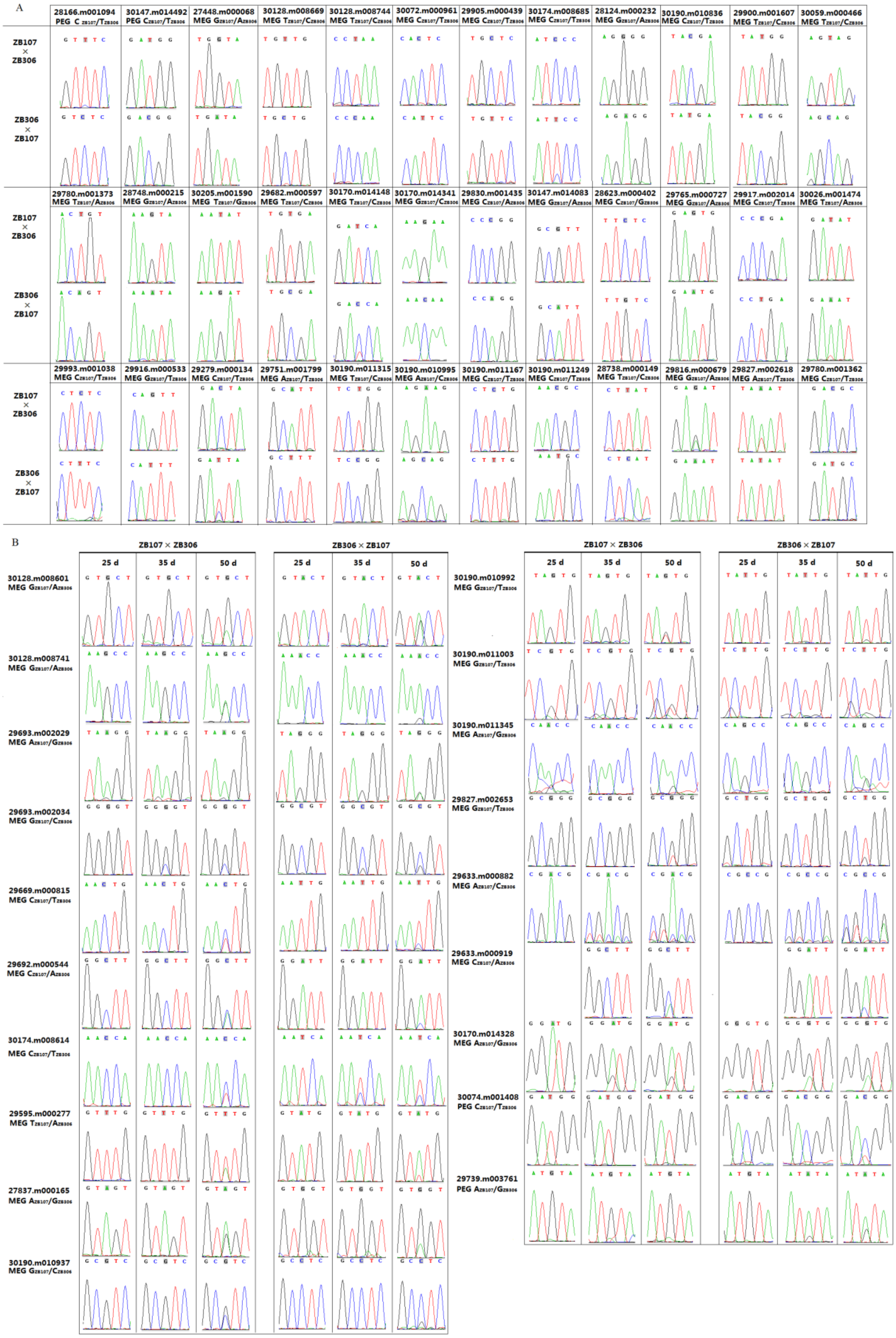

Supplementary Figure S3. Tissue-specific expression analysis for imprinted genes in root (Ro), stem (St), leaf (Le), female flower (Fe), pollen (Po) embryo (Em) and endosperm (En) tissues.

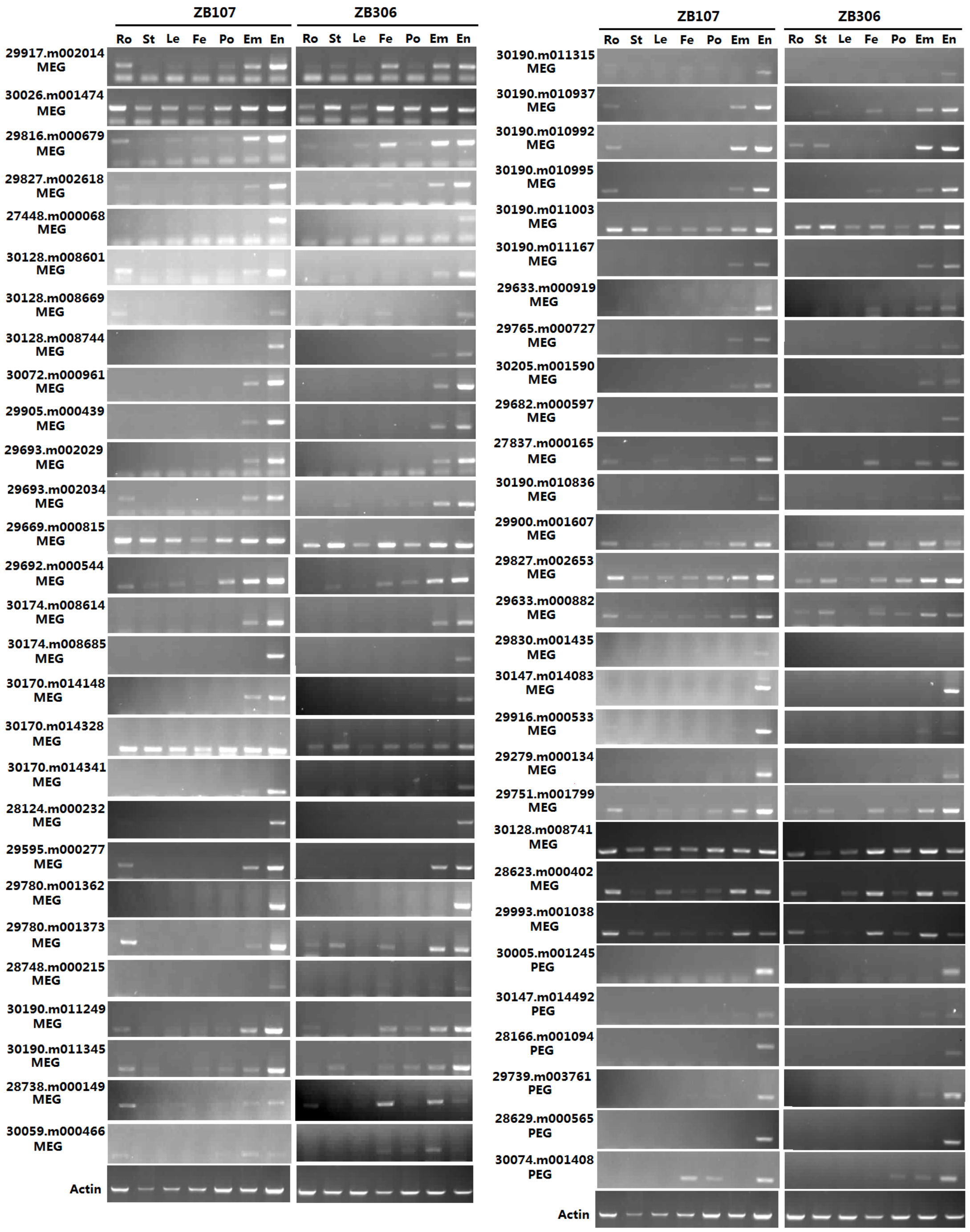

Supplementary Figure S4. Imprinted genes localized in mini-clusters were shown. The blue lines represent the location of imprinted genes on the scaffold (in green). The genomic structures of imprinted genes (in grey boxes) clustered within 10 kb are displayed.

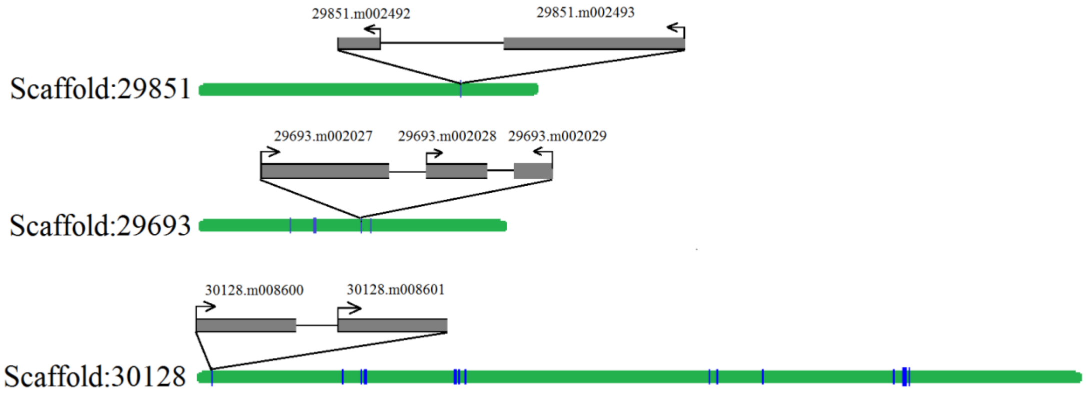

Supplementary Figure S5. Gene ontology analysis of identified imprinted genes. MEG represent all maternally expressed genes, all genes represent all annotated castor bean genes that have GO terms.

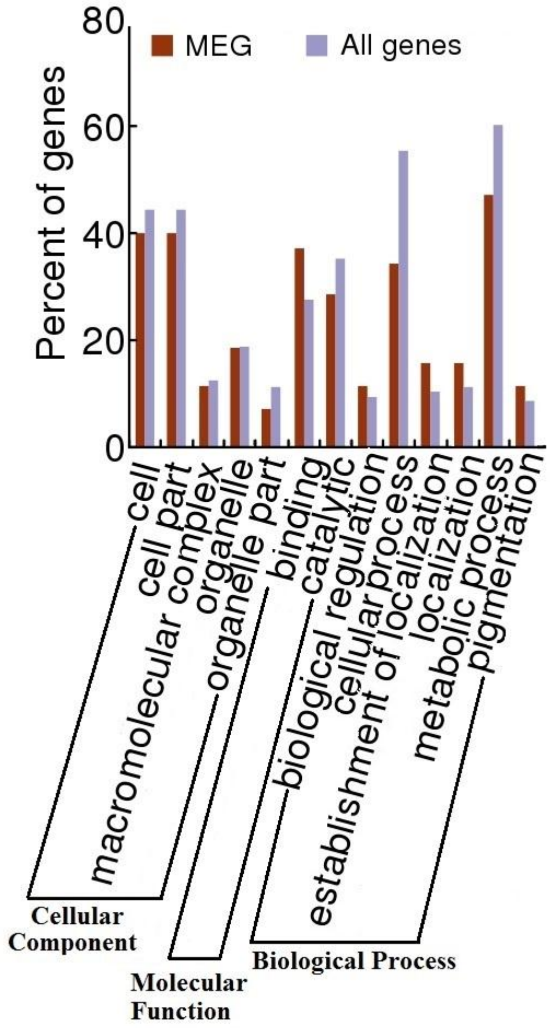

Supplementary Figure S6. Comparison of DNA methylation levels and gene expression level (RPKM) of imprinted genes identified between the endosperm (En) and embryo (Em) tissues. The green boxes represent the gene body regions. The black boxes represent transposable elements.

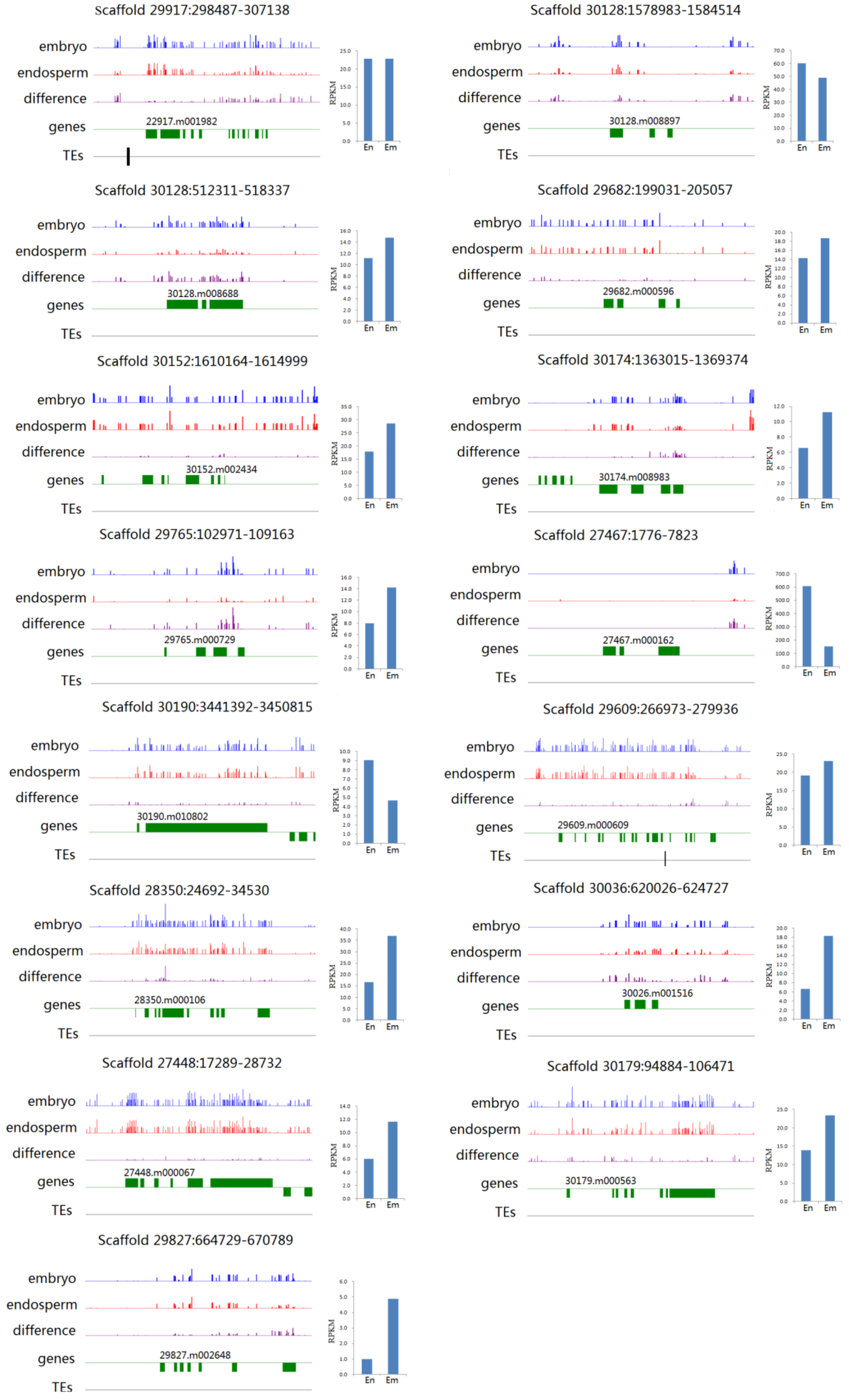

Supplement: SUPPLEMENTARY DATA [file supp_gku375_nar-00469-v-2014-File008.pdf]
