# Supplementary material for: Genomic imprinting, methylation and parent-of-origin effects in reciprocal hybrid endosperm of castor bean
Source: Nucleic Acids Res. 2014 May 5;42(11):6987–98. doi: 10.1093/nar/gku375 (PMC4066788; doi:10.1093/nar/gku375)
Supplement: SUPPLEMENTARY DATA [file supp_gku375_nar-00469-v-2014-File009.zip › Supplementary_Table_S9.docx]

**Supplementary Table S9.** Conserved imprinting identified in castor bean and other plant species including *Arabidopsis* (At), rice (Os) and maize(Zm). The marks * denote that these genes were experimentally confirmed.

| **Gene ID in Castor bean** | **Imprinting type**  **In castor bean** | **Gene ID in**  **At/Os/Zm** | **Imprinting type**  **In At/Os/Zm** | **Castor bean Description** | **Reference** |
| --- | --- | --- | --- | --- | --- |
| 29780.m001362 | MEG | AT1G14580 | MEG | indeterminate(ID)-domain 4 | Gehring et al., |
| 30170.m014221 | MEG | AT1G22180 | MEG | Sec14p-like phosphatidylinositol | Hsieh et al., |
| 29765.m000727 | MEG | AT3G08620 | MEG | nucleic acid binding protein, putative | Gehring et al., |
| 29827.m002644 | MEG | AT3G45870 | MEG | Auxin-induced protein 5NG4, putative | Gehring et al., |
| 29917.m002015 | MEG | AT3G50720* | PEG | Protein kinase | Wolff et al., |
| 30190.m011245 | MEG | AT4G11400 | PEG | ARID/BRIGHT DNA-binding domain-containing protein | Wolff et al., |
| 30170.m014165 | MEG | AT4G36120 | MEG | Myosin heavy chain, striated muscle, putative | Gehring et al., |
| 30147.m014492 | PEG | AT5G53150 | PEG | conserved hypothetical protein | Gehring et al., |
| 29682.m000597 | MEG | GRMZM2G374088* | MEG | big map kinase/bmk, putative | Waters et al., |
| 29765.m000727 | MEG | GRMZM2G472052 | PEG | nucleic acid binding protein, putative | Zhang et al., |
| 29827.m002656 | MEG | GRMZM2G341027 | PEG | conserved hypothetical protein | Zhang et al., |
| 30170.m013983 | MEG | GRMZM2G017845 | PEG | zinc ion binding protein, putative | Waters et al., |
| 30170.m014245 | MEG | GRMZM2G106798 | PEG | Squamosa promoter-binding protein, putative | Waters et al., |
| 30147.m014492 | PEG | GRMZM2G064905 | PEG | conserved hypothetical protein | Waters et al., |
| 29751.m001870 | MEG | GRMZM2G059453 | MEG | protein phosphatase 2c, putative | Xin et al., |
| 29828.m000387 | MEG | GRMZM2G149923 | MEG | calmodulin, putative | Xin et al., |
| 29900.m001577 | MEG | GRMZM2G419806 | PEG | Magnesium-chelatase subunit chlI, chloroplast precursor | Xin et al., |
| 30174.m008673 | MEG | GRMZM2G002786 | MEG | conserved hypothetical protein | Xin et al., |
| 30190.m011229 | MEG | GRMZM2G392975 | MEG | Aquaporin PIP1.3, putative | Xin et al., |
| 30147.m014492 | PEG | GRMZM2G064905 | PEG | conserved hypothetical protein | Xin et al., |
| 28350.m000106 | MEG | LOC_Os02g21430.1 | PEG | RNA-binding protein, putative | Luo et al., |
| S29634.m002088 | MEG | LOC_Os08g43480.1 | MEG | ring finger protein, putative | Luo et al., |
| 29765.m000727 | MEG | LOC_Os07g12490.1* | PEG | nucleic acid binding protein, putative | Luo et al., |
| 30179.m000563 | MEG | LOC_Os02g54510.1 | MEG | serine/threonine protein kinase, putative | Luo et al., |
| 30190.m011281 | meg | LOC_Os06g06870.1 | peg | sumo ligase, putative | Luo et al., |
