# Supplementary material for: Genomic imprinting, methylation and parent-of-origin effects in reciprocal hybrid endosperm of castor bean
Source: Nucleic Acids Res. 2014 May 5;42(11):6987–98. doi: 10.1093/nar/gku375 (PMC4066788; doi:10.1093/nar/gku375)
Supplement: SUPPLEMENTARY DATA [file supp_gku375_nar-00469-v-2014-File009.zip › Supplementary_Table_S1.docx]

| **Supplementary Table S1.** Primers used for the validation of gene imprinting based on RT-PCR sequencing. | | | | |
| --- | --- | --- | --- | --- |
| **Gene ID** | **SNP ID** | **Forward primer: 5'-3'** | **Reverse primer: 5'-3'** | **Imprinted type** |
| 27448.m000068 | 358.snp | ACGATCCCTAAGAGTGATGGAA | CCTGTTCTTGTTACGGCATACA | endosperm-MEG |
| 27837.m000165 | 3204.snp | ACTTTTGTGATACAGCCGACCT | CAAGCAGCAAAGAGAAGGAGAT | endosperm-MEG |
| 28124.m000232 | 3023.snp | TTCAGCCGGTTCTTCTCTTATC | TAGGGAGGAACTGGAACAAGAA | endosperm-MEG |
| 28623.m000402 | 6054.snp | TCCAACTTTCTTTGTTGCTCCT | TGAATGAAAAGTCCGGATCAAG | endosperm-MEG |
| 28738.m000149 | 7973.snp | ATCGATAGAGCTCGAGGACCTT | ATCAAAGGAGAGCACAGAAACC | endosperm-MEG |
| 28748.m000215 | 3425.snp | CAGCAGAAATCCTTGGAAATGT | TGAATAGAAAACTGAGGCAGCA | endosperm-MEG |
| 29279.m000134 | 6738.snp | ATGCTTTCCGTTCACAAATACC | TTAATGGCAGAGGAGTTGGAAT | endosperm-MEG |
| 29595.m000277 | 3098.snp | CTTTCTTGCTGAGGTTTCCACT | AAGATTCTCCTCGCTCAATCTG | endosperm-MEG |
| 29633.m000882 | 7680.snp | GAACAAGAGAAGCAACCCAAAC | AGCCCTTGAAGAAGCCTTTAGT | endosperm-MEG |
| 29633.m000919 | 7686.snp | AGGCAGAGAATCAAAATGGGTA | ATCGCAGCTGTTCACAACTTTA | endosperm-MEG |
| 29669.m000815 | 1596.snp | ATAGGCTGAAACCCATGTCATC | CTCTTCTGGCAAAGCTTGGTAT | endosperm-MEG |
| 29682.m000597 | 3802.snp | TGGTAAAAGTGGGCCTGTAGTT | AGACAGTTGCCTCTGAGATTCC | endosperm-MEG |
| 29692.m000544 | 2010.snp | TCCATTGAAGATCCCATTGTTC | CTCATTTCAAGCAAAGTCATGG | endosperm-MEG |
| 29693.m002029 | 1431.snp | GAGCGTATATGTCCACGTTCAA | TACTATCGGAAACGCTCCATCT | endosperm-MEG |
| 29693.m002034 | 1436.snp | TTACCAGCAACAATGCAATACC | AACCCCGCTCCATAAAAATACT | endosperm-MEG |
| 29751.m001799 | 6932.snp | AGTCCTGGGTAGTGGCATAGAA | GCTGCCTCGAGCTGTTATATCT | endosperm-MEG |
| 29758.m000646 | 4542.snp | AAGGCTTCCAATGAGGTACAGA | AACGAAATGGGAGGTACCCTAT | endosperm-MEG |
| 29765.m000727 | 3283.snp | ATTGGTCTTCCATTTGTCTGCT | TCAACAATAAAAAGGGCAGAGG | endosperm-MEG |
| 29780.m001362 | 3237.snp | GATCACCCATTATGTGGTTTGA | TCAAGTGACATTTTACGCCTTG | endosperm-MEG |
| 29780.m001373 | 3243.snp | CCTAGCAATGCGTTTAACATGA | ATCGGAGCATATCAAAATCAGC | endosperm-MEG |
| 29816.m000679 | 7236.snp | ATCAGCTGGAAAACTGTGGATT | ATATGGTCTCCCTTCTGGATGA | endosperm-MEG |
| 29827.m002618 | 7603.snp | AAGAGGGTGAAAAGTTGTTCCA | GACAATTCCTTTTTGAGCCATC | endosperm-MEG |
| 29827.m002653 | 7628.snp | CATGCCAGAATTAGCACTTGAA | AAGTTCCAGTTCCCAGAAAACA | endosperm-MEG |
| 29830.m001435 | 5447.snp | TGAAAGACGATCAAACCCTACA | AAGCTCAGGATTTCGATCAATG | endosperm-MEG |
| 29900.m001607 | 7478.snp | TATGGTGGCATACCCTGATACA | GAATCAGTATGGTGTTGCTCCA | endosperm-MEG |
| 29905.m000439 | 1236.snp | GAATTACTTCGGCAATGGTGTT | TGACTAAATTCCTCTCGTGCAT | endosperm-MEG |
| 29916.m000533 | 6502.snp | CTGTTTCTCGCTCATTCAACAC | TAACCAAAGTGTCATGCTCCAC | endosperm-MEG |
| 29917.m002014 | 2025.snp | GTTGGCTGTTGATAGCAGAGGT | TCAAATCATCACATGGTGTAAG | endosperm-MEG |
| 29923.m000812 | 623.snp | AAGAAGCAGGAAACTGTTGAGG | AAGATCTTTGCACGAGCCTTAG | endosperm-MEG |
| 29993.m001038 | 6095.snp | CTCCTTCTCCTCGATCCCTTAT | GGAGTAGCTCGAACCTCAAGAA | endosperm-MEG |
| 30026.m001474 | 2234.snp | TTGAACCGTTACATACCGACAG | GTGCCAATTATCTTGTCAGACG | endosperm-MEG |
| 30026.m001516 | 2251.snp | CTTTGCGAAGGAAGCACTAAAT | ACTTTTGCGTGAAGAAGACCAT | endosperm-MEG |
| 30059.m000466 | 8183.snp | GAAACTGATGACGAGATTGCTG | ATTATAGCTTCGCCTTCCCATC | endosperm-MEG |
| 30072.m000961 | 1046.snp | GCCTTCTCCATGAAATTACGTC | GAATCTCCTGAATTGTCGGAAG | endosperm-MEG |
| 30128.m008601 | 490.snp | TATGGCAGAGGAACAAGGATTT | GCTTTAGTGCCTTCCCAACTAA | endosperm-MEG |
| 30128.m008669 | 513.snp | GGAGCTTGCATCGTAACAGTAA | CAAAGGAACATGATCGGTTGAC | endosperm-MEG |
| 30128.m008741 | 531.snp | CGTGTGCTAATATCAACCGAAA | GTCGTGGTGAATGAAGATTTGA | endosperm-MEG |
| 30128.m008744 | 532.snp | TGAAGATGCAGACCTGTGTTCT | TGTTAGGCAAATGATGATGACC | endosperm-MEG |
| 30147.m014083 | 6023.snp | AATGCTCCAGTTCTCCAACAAT | CCATGCATTACTTTGAACCTCA | endosperm-MEG |
| 30170.m013704 | 4146.snp | CAAACATCATATGCAACCAAGG | AGCATATACTTGCGAAACAGCA | endosperm-MEG |
| 30170.m014093 | 4157.snp | GCCGTTTTAGTGAGGGTACAAG | GTTTCTTTGCCTCGTTCAGTTT | endosperm-MEG |
| 30170.m014148 | 4163.snp | ATAGCGAGATCTCTTGGCAAAC | GGAGTGTTTTCCCTTCTTCCTT | endosperm-MEG |
| 30170.m014221 | 4149.snp | AGCAAACTTTCGTGACAGACAA | CCAAAGTCTGTTGGAAGATTCA | endosperm-MEG |
| 30170.m014328 | 4190.snp | AGCGTTTCTGGTTGGAGCTT | ATGCCGCCAACCCAATACTA | endosperm-MEG |
| 30170.m014341 | 4195.snp | TAAATCAGCCACCCCAACTTAC | ATATCAAGATCCCGATCACCAG | endosperm-MEG |
| 30174.m008614 | 2562.snp | TCTGCTACTGATGCCAAGAGAA | TAGACGACGTGTAAGGTGGTTG | endosperm-MEG |
| 30174.m008685 | 2631.snp | TGGCAAGAGAACTTGTGACACT | GCCACCAGGTAAGATTTCATTC | endosperm-MEG |
| 30174.m008690 | 2637.snp | GTGGTTGACATCTTGGACTGAA | GACATTTCTGTGACCACTTCCA | endosperm-MEG |
| 30190.m010836 | 7128.snp | TCTGGAAAGCTTGAGAATGATG | CTTTGATGTGTCAAACGATTCC | endosperm-MEG |
| 30190.m010937 | 7013.snp | TGTTTCCTCCATCAGTGAATTG | TCCAAATATCTTCCACGGATTC | endosperm-MEG |
| 30190.m010992 | 7027.snp | ATGCCATACTGTGTCAACGAAC | CACATATGCTGCTGGATCTCAT | endosperm-MEG |
| 30190.m010995 | 7029.snp | CAAGATGATGCAGGAGATTCAG | TCAACAGCCTTGTCACAGTCTT | endosperm-MEG |
| 30190.m011003 | 7030.snp | GCGCTACTCTTCTTAACCACCA | CAGCTTTGCTACAAGCACTGAG | endosperm-MEG |
| 30190.m011167 | 7048.snp | ATTCTCCTTCAAGTGTGGCTGT | AAGAATAGCGACAAACCTGAGC | endosperm-MEG |
| 30190.m011249 | 7074.snp | GTACTACCATGTGGTGCTGGAA | GCAGTTAGCCCGAGTTTACAGT | endosperm-MEG |
| 30190.m011315 | 6995.snp | CACTGTGATCAAAGGGACCATA | ACAGAGAGCATTGAAGTGAGCA | endosperm-MEG |
| 30190.m011345 | 7109.snp | CAGATAGGCCTTCAGCACTTTT | CACATCAACGCAGCTCTAATTC | endosperm-MEG |
| 30205.m001590 | 3436.snp | TTCAGCTACCCCAAGATCCTTA | GCTTAGGCACTGGTTTTCACTT | endosperm-MEG |
| 30074.m001408 | 2212.snp | CAAGATTGCTCAACTCTCGTCA | TCCCAGAAAAGTAACAAGCACA | endosperm-PEG |
| 30074.m001408 | 2214.snp | TCAAGTCTTTGTGCAACTCCAG | GGCGTTGTCTAGGGTAAATGTT | endosperm-PEG |
| 30074.m001409 | 244.snp | GATTAACAGGCCAAGATGAAGG | TGAAGCCTCAGTTTGAAGAACA | endosperm-PEG |
| 28166.m001094 | 4035.snp | GGCTTTATGGCTGTGGTTAGAG | ATGGTGGGTAAGATCCCTTTTT | endosperm-PEG |
| 29739.m003761 | 4036.snp | GATCAGTTTGCTTGCCAAAGTA | TTTCCTATCCTTAGGTGGAACG | endosperm-PEG |
| 29739.m003761 | 5759.snp | CAGACGTCGATCCTGATATGAA | TGCCTATCCAGAAGTCTCCATT | endosperm-PEG |
| 28629.m000565 | 6910.snp | AAGTTCTCCTTTGCTCTGATGC | TCTGCGTCCTTATTTTCTCAGG | endosperm-PEG |
| 30005.m001245 | 7646.snp | GAAGTTGGTTGCAGTGATGAAG | ACTTATGGATCCACCAAGCTGT | endosperm-PEG |
| 29693.m001994 | 1418.snp | ACCGGTTCAAGTGTGGTAGTTT | CCATCCACTAGATATGCGCTAA | embryo-MEG |
| 29709.m001197 | 3927.snp | AGTGAGGTTCAATGTGCTGAAG | AATTCACAAGCAAGAGCAGCTT | embryo-MEG |
| 29709.m001207 | 3930.snp | GGGATGGAAACACTTTCTTCAA | CAAGAACCGCAGGTATAACACA | embryo-MEG |
| 29633.m000883 | 7684.snp | ATCAAGTGTCTGATGCTGAGGA | GGGAACAAATCCAGCTGAGTAA | embryo-MEG |
| 28200.m000195 | 5058.snp | AGAGCTTGTAAAGTGGGACCAA | CCAATAACCCATGAAAGGATTG | embryo-MEG |
| 30174.m008614 | 2564.snp | GCTGCATTGTACCACCAGATAA | GAAGCACACTCATTTGGGACTT | embryo-PEG |
| 29333.m001057 | 3109.snp | TTTGGGTTCGTCTCTTTTGAGT | GCTTCGAATCTTGCCATTATGT | embryo-PEG |
| 29912.m005280 | 5278.snp | CATGCAAGGGTTGATTAATGTG | TTAAGGACTTCACCAGCGATTT | embryo-PEG |
| 28597.m000122 | 1858.snp | CGTGAATTTGAAGCAGTGAAAG | CCTCAAAGATGAAGCCACTTTT | cis-ZB107 |
| 30114.m000526 | 1950.snp | GATCATAGCAACCTTCCCTCTG | GGAAGCTCAAGAACCCTGTCTA | cis-ZB107 |
| 29904.m002897 | 3317.snp | AAAGGCTAGGGAAAAGTTGAGG | ATCTCCATCAACTCTGGCATCT | cis-ZB107 |
| 30027.m000848 | 4787.snp | CGTTTTCCATGGCTACTCACTA | CCATTAAGGAGTTTTGGAGCAC | cis-ZB107 |
| 28492.m000467 | 5083.snp | CATCCAAGTCACTGAAGATCCA | GCAATAGTGATCAGCAGAGGAA | cis-ZB107 |
| 29785.m000971 | 1027.snp | TCACACTCTCACACTCACTTCA | CCAGACTGTCCAAACACAAAGT | cis-ZB306 |
| 29726.m004084 | 1810.snp | GTTTTCTCCAGTTCTACTCGGC | GCAGCAGTCCATGATCTTAGTG | cis-ZB306 |
| 29629.m001355 | 3829.snp | GTCACCATCAGAATCACAGTCG | GCCTCTCAGATCATTTTCACCC | cis-ZB306 |
