# Supplementary material for: Genomic imprinting, methylation and parent-of-origin effects in reciprocal hybrid endosperm of castor bean
Source: Nucleic Acids Res. 2014 May 5;42(11):6987–98. doi: 10.1093/nar/gku375 (PMC4066788; doi:10.1093/nar/gku375)
Supplement: SUPPLEMENTARY DATA [file supp_gku375_nar-00469-v-2014-File009.zip › Supplementary_Table_S3.docx]

| **Supplementary Table S3.** Genes with maternally-biased expression (MEGs, FDR<0.05) in both hybrid endosperms. m, Maternal alleles; p, Paternal alleles. | | | | | | | | | | |
| --- | --- | --- | --- | --- | --- | --- | --- | --- | --- | --- |
| **SNP No.** | **Scaffold** | **Position** | **SNP_ZB107** | **SNP_ZB306** | **ZB107×ZB306** | | **ZB306×ZB107** | | **p-value** | **FDR** |
|  |  |  |  |  | **m_ZB107** | **p_ZB306** | **m_ZB306** | **p_ZB107** |  |  |
| 52.snp | 27471 | 16926 | G | A | 1057 | 53 | 700 | 184 | 2.1568e-25 | 2.9326e-24 |
| 68.snp | 27471 | 168030 | G | A | 10 | 0 | 10 | 0 | 9.7656e-04 | 6.3948e-03 |
| 71.snp | 28725 | 43292 | G | C | 71 | 3 | 101 | 32 | 2.2586e-03 | 1.3776e-02 |
| 72.snp | 28725 | 43409 | C | T | 47 | 6 | 81 | 25 | 4.0983e-03 | 2.3534e-02 |
| 86.snp | 29745 | 106238 | C | A | 26 | 4 | 20 | 3 | 3.2917e-03 | 1.9642e-02 |
| 199.snp | 29792 | 198561 | C | T | 10 | 0 | 77 | 1 | 5.5620e-21 | 7.3293e-20 |
| 200.snp | 30068 | 15645 | T | G | 123 | 27 | 118 | 34 | 9.1161e-05 | 7.0894e-04 |
| 201.snp | 30068 | 41581 | G | A | 121 | 7 | 82 | 12 | 2.7138e-09 | 2.9148e-08 |
| 202.snp | 30068 | 75824 | A | G | 66 | 4 | 116 | 17 | 1.9513e-12 | 2.2983e-11 |
| 206.snp | 30068 | 337763 | G | A | 27 | 3 | 59 | 8 | 1.6195e-04 | 1.2163e-03 |
| 212.snp | 30068 | 896517 | C | T | 10867 | 105 | 9500 | 1401 | 0 | 0 |
| 213.snp | 30068 | 906096 | G | A | 46 | 4 | 94 | 31 | 5.0642e-03 | 2.8263e-02 |
| 223.snp | 30068 | 1004195 | C | T | 150 | 6 | 258 | 47 | 1.1434e-19 | 1.4838e-18 |
| 298.snp | 28565 | 49913 | T | A | 29 | 1 | 21 | 3 | 3.2917e-03 | 1.9752e-02 |
| 299.snp | 28565 | 61069 | G | A | 30 | 6 | 126 | 40 | 2.7541e-03 | 1.6679e-02 |
| 356.snp | 27448 | 22694 | G | T | 16 | 1 | 33 | 7 | 4.0851e-03 | 2.3538e-02 |
| 358.snp | 27448 | 28491 | G | A | 132 | 6 | 88 | 1 | 2.4423e-06 | 2.2281e-05 |
| 372.snp | 29839 | 123752 | T | G | 76 | 1 | 49 | 10 | 2.7802e-04 | 2.0222e-03 |
| 374.snp | 29839 | 213331 | A | G | 112 | 18 | 58 | 17 | 6.2317e-03 | 3.3764e-02 |
| 419.snp | 28350 | 30030 | A | G | 34 | 0 | 38 | 7 | 3.6699e-04 | 2.6445e-03 |
| 431.snp | 29610 | 111285 | C | T | 146 | 10 | 134 | 34 | 9.7326e-07 | 9.2136e-06 |
| 433.snp | 29610 | 116390 | G | A | 30 | 0 | 38 | 9 | 3.7734e-03 | 2.2360e-02 |
| 450.snp | 29673 | 82290 | T | C | 175 | 44 | 116 | 38 | 1.7413e-03 | 1.0845e-02 |
| 457.snp | 29673 | 222451 | A | G | 24 | 4 | 71 | 21 | 3.9359e-03 | 2.2801e-02 |
| 458.snp | 29673 | 232083 | A | C | 17 | 0 | 20 | 0 | 3.2917e-03 | 1.9711e-02 |
| 459.snp | 29673 | 250451 | T | C | 47 | 8 | 78 | 15 | 4.9006e-05 | 3.9403e-04 |
| 462.snp | 29673 | 272901 | A | G | 165 | 44 | 45 | 11 | 3.3193e-03 | 1.9765e-02 |
| 472.snp | 29673 | 326477 | T | C | 16 | 0 | 17 | 0 | 4.2811e-03 | 2.4403e-02 |
| 490.snp | 30128 | 45520 | G | A | 118 | 12 | 104 | 27 | 2.3699e-05 | 1.9743e-04 |
| 509.snp | 30128 | 394703 | T | G | 249 | 12 | 121 | 41 | 3.5285e-03 | 2.0996e-02 |
| 513.snp | 30128 | 441911 | T | C | 125 | 0 | 109 | 20 | 4.7002e-08 | 4.8124e-07 |
| 522.snp | 30128 | 514696 | G | T | 41 | 5 | 20 | 2 | 5.0450e-04 | 3.4995e-03 |
| 523.snp | 30128 | 514763 | T | G | 30 | 3 | 22 | 4 | 5.9002e-03 | 3.2358e-02 |
| 530.snp | 30128 | 790197 | C | T | 25 | 0 | 22 | 0 | 5.9002e-03 | 3.2338e-02 |
| 531.snp | 30128 | 797269 | G | A | 100 | 7 | 64 | 10 | 3.6737e-07 | 3.5769e-06 |
| 532.snp | 30128 | 814607 | T | C | 114 | 5 | 113 | 7 | 1.9726e-06 | 1.8309e-05 |
| 533.snp | 30128 | 836801 | G | A | 140 | 15 | 133 | 42 | 4.4462e-04 | 3.1481e-03 |
| 536.snp | 30128 | 1153453 | A | T | 104 | 3 | 400 | 69 | 6.6212e-25 | 8.9887e-24 |
| 545.snp | 30128 | 1582552 | A | G | 309 | 6 | 303 | 8 | 1.4750e-11 | 1.6816e-10 |
| 547.snp | 30128 | 1609590 | G | T | 23 | 1 | 34 | 5 | 1.1283e-04 | 8.7188e-04 |
| 548.snp | 30128 | 1614824 | T | C | 39 | 2 | 21 | 3 | 3.2917e-03 | 1.9683e-02 |
| 549.snp | 30128 | 1617345 | A | G | 157 | 18 | 178 | 63 | 1.2398e-03 | 7.9779e-03 |
| 551.snp | 30128 | 1744911 | G | A | 57 | 5 | 25 | 4 | 2.4051e-03 | 1.4638e-02 |
| 587.snp | 30128 | 2139523 | C | T | 36 | 4 | 55 | 10 | 2.5986e-05 | 2.1522e-04 |
| 594.snp | 30128 | 2189060 | T | C | 15 | 0 | 14 | 0 | 1.5259e-03 | 9.6374e-03 |
| 595.snp | 30128 | 2195151 | A | T | 27 | 2 | 42 | 7 | 6.6098e-05 | 5.2116e-04 |
| 596.snp | 30128 | 2195334 | G | A | 51 | 2 | 68 | 21 | 6.6304e-03 | 3.5586e-02 |
| 620.snp | 29923 | 215012 | A | G | 86 | 6 | 122 | 25 | 8.2098e-09 | 8.6334e-08 |
| 623.snp | 29923 | 261767 | G | A | 349 | 33 | 257 | 79 | 4.1532e-07 | 4.0392e-06 |
| 631.snp | 29923 | 470392 | C | T | 31 | 3 | 30 | 7 | 8.6751e-03 | 4.5113e-02 |
| 639.snp | 28202 | 3410 | C | A | 33 | 0 | 91 | 22 | 2.4043e-05 | 1.9991e-04 |
| 640.snp | 28202 | 12146 | A | C | 57 | 2 | 58 | 11 | 1.8697e-05 | 1.5776e-04 |
| 659.snp | 30025 | 76692 | A | G | 37 | 1 | 32 | 1 | 2.8564e-04 | 2.0723e-03 |
| 678.snp | 30075 | 418220 | C | T | 208 | 6 | 93 | 30 | 4.5629e-03 | 2.5752e-02 |
| 683.snp | 30075 | 460147 | G | A | 62 | 2 | 57 | 11 | 1.6777e-03 | 1.0488e-02 |
| 735.snp | 27955 | 126669 | T | C | 70 | 3 | 58 | 16 | 2.9242e-03 | 1.7609e-02 |
| 738.snp | 27955 | 130423 | T | A | 13 | 0 | 12 | 0 | 3.9062e-03 | 2.2675e-02 |
| 853.snp | 30069 | 298275 | G | A | 2649 | 158 | 1632 | 411 | 3.6531e-62 | 5.2603e-61 |
| 945.snp | 30162 | 1086214 | A | G | 31 | 2 | 20 | 3 | 3.2917e-03 | 1.9628e-02 |
| 1008.snp | 29977 | 49190 | A | G | 26 | 0 | 36 | 2 | 8.3862e-04 | 5.6209e-03 |
| 1016.snp | 29977 | 84701 | A | G | 20 | 1 | 34 | 4 | 4.0293e-05 | 3.2862e-04 |
| 1046.snp | 30072 | 243949 | C | T | 639 | 24 | 905 | 279 | 1.7644e-20 | 2.3072e-19 |
| 1047.snp | 30072 | 244261 | C | T | 722 | 87 | 1323 | 114 | 1.4270e-21 | 1.8951e-20 |
| 1049.snp | 30072 | 282071 | A | G | 73 | 10 | 164 | 47 | 4.0958e-06 | 3.6660e-05 |
| 1052.snp | 30072 | 285329 | C | T | 36 | 1 | 46 | 3 | 1.1068e-05 | 9.6049e-05 |
| 1056.snp | 29851 | 541749 | C | T | 473 | 64 | 106 | 33 | 9.8709e-04 | 6.4489e-03 |
| 1064.snp | 29851 | 808224 | G | A | 59 | 3 | 84 | 13 | 5.3776e-09 | 5.6901e-08 |
| 1065.snp | 29851 | 808403 | A | C | 28 | 1 | 51 | 8 | 8.6148e-06 | 7.5294e-05 |
| 1066.snp | 29851 | 809006 | G | A | 53 | 0 | 128 | 14 | 2.2204e-16 | 2.7841e-15 |
| 1067.snp | 29851 | 812549 | G | A | 35 | 3 | 222 | 37 | 2.2189e-06 | 2.0528e-05 |
| 1077.snp | 30138 | 127551 | G | A | 82 | 8 | 332 | 15 | 1.0437e-10 | 1.1665e-09 |
| 1174.snp | 30138 | 1310673 | C | T | 10 | 0 | 10 | 0 | 0 | 0 |
| 1232.snp | 29578 | 63421 | C | T | 293 | 41 | 207 | 80 | 8.0447e-03 | 4.2482e-02 |
| 1234.snp | 29905 | 23505 | G | T | 78 | 2 | 72 | 20 | 1.0985e-03 | 7.1550e-03 |
| 1235.snp | 29905 | 29584 | T | C | 163 | 51 | 344 | 103 | 8.1550e-05 | 6.4062e-04 |
| 1236.snp | 29905 | 179083 | C | T | 160 | 16 | 98 | 16 | 1.1077e-09 | 1.2034e-08 |
| 1286.snp | 29624 | 327206 | G | A | 26 | 0 | 50 | 9 | 3.5857e-05 | 2.9441e-04 |
| 1392.snp | 29883 | 469880 | T | C | 537 | 172 | 735 | 164 | 1.9852e-11 | 2.2602e-10 |
| 1416.snp | 29693 | 303394 | T | C | 85 | 1 | 179 | 29 | 3.4492e-16 | 4.3186e-15 |
| 1417.snp | 29693 | 304019 | C | T | 72 | 4 | 66 | 18 | 1.3310e-03 | 8.5199e-03 |
| 1418.snp | 29693 | 304535 | T | C | 89 | 10 | 105 | 12 | 4.2325e-12 | 4.8839e-11 |
| 1423.snp | 29693 | 364798 | T | A | 320 | 8 | 95 | 2 | 1.0135e-11 | 1.1617e-10 |
| 1424.snp | 29693 | 366439 | C | T | 807 | 101 | 114 | 27 | 1.0327e-06 | 9.7543e-06 |
| 1425.snp | 29693 | 502299 | G | T | 278 | 56 | 317 | 123 | 1.3655e-03 | 8.7338e-03 |
| 1426.snp | 29693 | 510973 | G | T | 181 | 15 | 73 | 19 | 4.9466e-04 | 3.4509e-03 |
| 1429.snp | 29693 | 517068 | A | C | 16 | 1 | 50 | 3 | 4.6156e-04 | 3.2492e-03 |
| 1430.snp | 29693 | 522211 | G | T | 44 | 5 | 51 | 9 | 3.5857e-05 | 2.9413e-04 |
| 1431.snp | 29693 | 524314 | A | G | 198 | 11 | 305 | 80 | 3.9764e-12 | 4.5947e-11 |
| 1436.snp | 29693 | 541825 | G | C | 166 | 2 | 199 | 2 | 3.8169e-06 | 3.4272e-05 |
| 1437.snp | 29693 | 541857 | T | C | 69 | 21 | 121 | 22 | 6.6304e-03 | 3.5608e-02 |
| 1438.snp | 29693 | 541918 | G | T | 234 | 8 | 267 | 52 | 2.4549e-18 | 3.1475e-17 |
| 1456.snp | 29693 | 823506 | T | A | 41 | 3 | 29 | 6 | 6.1394e-03 | 3.3519e-02 |
| 1491.snp | 30045 | 478920 | C | G | 95 | 28 | 61 | 16 | 1.5989e-03 | 1.0031e-02 |
| 1519.snp | 30099 | 357602 | T | A | 10 | 0 | 10 | 0 | 0 | 0 |
| 1526.snp | 30099 | 397106 | C | A | 10 | 0 | 10 | 0 | 0 | 0 |
| 1563.snp | 29660 | 67991 | G | T | 421 | 5 | 574 | 188 | 2.7845e-11 | 3.1534e-10 |
| 1565.snp | 29660 | 145721 | G | A | 16 | 1 | 44 | 11 | 3.3193e-03 | 1.9779e-02 |
| 1579.snp | 27467 | 5670 | A | T | 265 | 7 | 127 | 8 | 1.0993e-13 | 1.3277e-12 |
| 1582.snp | 29669 | 34025 | C | T | 58 | 4 | 25 | 3 | 4.5245e-04 | 3.1983e-03 |
| 1583.snp | 29669 | 38143 | G | C | 177 | 8 | 195 | 75 | 8.8576e-03 | 4.5979e-02 |
| 1584.snp | 29669 | 73220 | A | C | 61 | 4 | 25 | 5 | 9.2645e-03 | 4.7974e-02 |
| 1596.snp | 29669 | 151081 | C | T | 1144 | 28 | 790 | 16 | 1.0191e-43 | 1.4383e-42 |
| 1597.snp | 29669 | 181752 | A | C | 27 | 5 | 33 | 7 | 4.0851e-03 | 2.3570e-02 |
| 1615.snp | 29813 | 199282 | A | G | 1006 | 58 | 550 | 223 | 3.9319e-04 | 2.8072e-03 |
| 1653.snp | 28592 | 94190 | G | A | 22 | 2 | 40 | 7 | 1.6742e-04 | 1.2551e-03 |
| 1655.snp | 28592 | 97287 | A | C | 28 | 0 | 52 | 6 | 1.0501e-07 | 1.0500e-06 |
| 1658.snp | 29599 | 13596 | T | G | 55 | 2 | 84 | 22 | 1.5632e-04 | 1.1802e-03 |
| 1752.snp | 29726 | 637021 | T | C | 10 | 0 | 10 | 0 | 0 | 0 |
| 1844.snp | 28583 | 35439 | C | A | 25 | 0 | 107 | 11 | 9.2645e-03 | 4.7945e-02 |
| 1884.snp | 29681 | 244957 | C | T | 168 | 23 | 131 | 46 | 5.2022e-03 | 2.9015e-02 |
| 1903.snp | 29585 | 14910 | C | T | 510 | 9 | 355 | 5 | 1.1283e-04 | 8.7267e-04 |
| 1904.snp | 29585 | 91659 | T | C | 39 | 1 | 66 | 14 | 3.0417e-05 | 2.5071e-04 |
| 1911.snp | 29585 | 148178 | G | T | 61 | 5 | 75 | 23 | 5.2095e-03 | 2.9037e-02 |
| 1919.snp | 28962 | 84014 | A | C | 186 | 32 | 187 | 47 | 8.7133e-09 | 9.1516e-08 |
| 1941.snp | 29836 | 192938 | C | T | 184 | 21 | 454 | 174 | 6.6066e-05 | 5.2187e-04 |
| 1942.snp | 29836 | 195095 | A | G | 257 | 42 | 500 | 208 | 2.4832e-03 | 1.5081e-02 |
| 1984.snp | 30010 | 15706 | C | T | 10 | 0 | 10 | 0 | 0 | 0 |
| 2005.snp | 29692 | 121838 | C | T | 69 | 3 | 51 | 1 | 3.5857e-05 | 2.9384e-04 |
| 2006.snp | 29692 | 148436 | G | A | 43 | 1 | 34 | 7 | 1.8736e-03 | 1.1551e-02 |
| 2010.snp | 29692 | 283134 | C | A | 3105 | 47 | 953 | 24 | 3.7353e-36 | 5.1948e-35 |
| 2011.snp | 29917 | 301451 | C | A | 70 | 5 | 24 | 2 | 5.4543e-05 | 4.3609e-04 |
| 2014.snp | 29917 | 484400 | C | A | 68 | 4 | 31 | 7 | 8.6751e-03 | 4.5196e-02 |
| 2023.snp | 29917 | 544581 | C | A | 13 | 0 | 25 | 2 | 9.2645e-03 | 4.7916e-02 |
| 2024.snp | 29917 | 586140 | C | A | 52 | 5 | 69 | 13 | 4.3602e-06 | 3.8986e-05 |
| 2025.snp | 29917 | 588359 | C | T | 26 | 1 | 18 | 1 | 1.3734e-04 | 1.0518e-03 |
| 2026.snp | 29917 | 597353 | A | T | 29 | 0 | 29 | 4 | 3.6906e-04 | 2.6550e-03 |
| 2081.snp | 30131 | 1532311 | C | T | 10 | 0 | 10 | 0 | 0 | 0 |
| 2228.snp | 30026 | 229514 | C | T | 52 | 14 | 222 | 83 | 3.5503e-03 | 2.1096e-02 |
| 2229.snp | 30026 | 230875 | C | T | 53 | 6 | 280 | 104 | 4.5199e-04 | 3.1976e-03 |
| 2230.snp | 30026 | 233212 | G | A | 153 | 10 | 456 | 116 | 1.3835e-18 | 1.7818e-17 |
| 2234.snp | 30026 | 273291 | A | T | 1204 | 37 | 368 | 73 | 4.7406e-24 | 6.3648e-23 |
| 2242.snp | 30026 | 346711 | C | T | 54 | 7 | 39 | 4 | 2.3836e-06 | 2.1815e-05 |
| 2243.snp | 30026 | 347351 | C | T | 47 | 2 | 49 | 8 | 2.0715e-05 | 1.7291e-04 |
| 2251.snp | 30026 | 622314 | A | G | 20 | 0 | 18 | 3 | 8.5165e-03 | 4.4697e-02 |
| 2276.snp | 29969 | 108161 | G | A | 45 | 4 | 17 | 2 | 4.2811e-03 | 2.4469e-02 |
| 2344.snp | 29822 | 703938 | C | T | 10 | 0 | 10 | 0 | 0 | 0 |
| 2371.snp | 29822 | 1106408 | G | A | 10 | 0 | 10 | 0 | 0 | 0 |
| 2386.snp | 29930 | 427052 | T | A | 28 | 1 | 15 | 1 | 1.5259e-03 | 9.6732e-03 |
| 2399.snp | 30174 | 179219 | T | C | 10 | 0 | 48 | 0 | 9.7656e-04 | 6.4692e-03 |
| 2409.snp | 30174 | 213769 | G | C | 35 | 3 | 14 | 1 | 1.5259e-03 | 9.6660e-03 |
| 2410.snp | 30174 | 257857 | A | C | 13 | 0 | 10 | 0 | 2.4414e-04 | 1.8034e-03 |
| 2439.snp | 30174 | 503367 | C | T | 42 | 1 | 25 | 3 | 4.5245e-04 | 3.1956e-03 |
| 2468.snp | 30174 | 776256 | A | C | 142 | 47 | 157 | 47 | 7.4961e-04 | 5.0720e-03 |
| 2526.snp | 30174 | 1365035 | C | T | 48 | 2 | 29 | 4 | 3.6906e-04 | 2.6527e-03 |
| 2550.snp | 30174 | 1683092 | T | C | 10 | 0 | 19 | 2 | 3.9062e-03 | 2.3004e-02 |
| 2562.snp | 30174 | 1749515 | C | T | 2798 | 49 | 2127 | 78 | 1.6222e-21 | 2.1475e-20 |
| 2580.snp | 30174 | 1811186 | G | A | 124 | 5 | 248 | 79 | 2.4498e-06 | 2.2326e-05 |
| 2593.snp | 30174 | 1903657 | C | G | 246 | 30 | 312 | 106 | 3.9645e-06 | 3.5560e-05 |
| 2594.snp | 30174 | 1903757 | G | A | 157 | 17 | 203 | 72 | 7.6320e-04 | 5.1558e-03 |
| 2599.snp | 30174 | 1967313 | G | A | 147 | 2 | 96 | 29 | 9.4778e-04 | 6.3030e-03 |
| 2607.snp | 30174 | 2086835 | G | A | 157 | 20 | 303 | 33 | 2.2521e-14 | 2.7472e-13 |
| 2611.snp | 30174 | 2109678 | G | A | 114 | 8 | 99 | 2 | 1.1372e-05 | 9.8382e-05 |
| 2613.snp | 30174 | 2111290 | C | A | 177 | 14 | 112 | 35 | 7.8495e-04 | 5.2944e-03 |
| 2630.snp | 30174 | 2178050 | G | A | 383 | 14 | 45 | 12 | 8.0158e-03 | 4.2408e-02 |
| 2631.snp | 30174 | 2178228 | C | T | 330 | 25 | 33 | 0 | 5.1803e-05 | 4.1574e-04 |
| 2637.snp | 30174 | 2201450 | T | A | 10 | 0 | 16 | 0 | 9.7656e-04 | 6.4592e-03 |
| 2652.snp | 30174 | 2278563 | T | A | 20 | 0 | 13 | 0 | 4.9439e-03 | 2.7829e-02 |
| 2717.snp | 30174 | 2697668 | A | T | 391 | 38 | 195 | 65 | 1.4307e-04 | 1.0830e-03 |
| 2743.snp | 30174 | 3130787 | G | A | 57 | 0 | 47 | 0 | 1.6777e-03 | 1.0495e-02 |
| 2967.snp | 29929 | 1327212 | C | T | 10 | 0 | 10 | 0 | 0 | 0 |
| 3023.snp | 28124 | 8721 | G | A | 111 | 1 | 192 | 3 | 4.8106e-14 | 5.8432e-13 |
| 3056.snp | 27687 | 29009 | T | A | 277 | 71 | 314 | 125 | 3.2436e-03 | 1.9478e-02 |
| 3058.snp | 28507 | 28349 | C | T | 36 | 1 | 37 | 6 | 2.1412e-04 | 1.5899e-03 |
| 3060.snp | 28507 | 63373 | G | A | 621 | 21 | 425 | 141 | 2.3522e-08 | 2.4406e-07 |
| 3061.snp | 28507 | 63457 | G | A | 694 | 49 | 507 | 173 | 1.0719e-08 | 1.1230e-07 |
| 3064.snp | 28507 | 129133 | T | C | 12 | 0 | 34 | 4 | 2.4414e-04 | 1.8018e-03 |
| 3066.snp | 28507 | 130906 | G | A | 22 | 0 | 34 | 8 | 5.7031e-03 | 3.1561e-02 |
| 3069.snp | 28507 | 135599 | G | T | 229 | 39 | 172 | 57 | 2.3627e-04 | 1.7498e-03 |
| 3098.snp | 29595 | 4705 | T | A | 1908 | 11 | 749 | 2 | 9.2298e-15 | 1.1422e-13 |
| 3204.snp | 27837 | 97014 | A | G | 852 | 18 | 1185 | 30 | 1.6296e-19 | 2.1083e-18 |
| 3231.snp | 29780 | 41166 | A | T | 83 | 8 | 64 | 4 | 6.3087e-05 | 4.9879e-04 |
| 3232.snp | 29780 | 54599 | A | T | 31 | 7 | 57 | 14 | 8.6751e-03 | 4.5168e-02 |
| 3234.snp | 29780 | 228276 | G | A | 20 | 3 | 23 | 4 | 5.9002e-03 | 3.2379e-02 |
| 3235.snp | 29780 | 289578 | G | A | 90 | 2 | 166 | 44 | 2.6603e-07 | 2.6081e-06 |
| 3237.snp | 29780 | 348335 | C | T | 702 | 83 | 1040 | 307 | 9.6395e-27 | 1.3149e-25 |
| 3243.snp | 29780 | 443091 | T | A | 180 | 14 | 213 | 40 | 2.7983e-13 | 3.3560e-12 |
| 3244.snp | 29780 | 443217 | A | G | 67 | 7 | 72 | 6 | 6.4115e-10 | 7.0190e-09 |
| 3245.snp | 29780 | 448732 | T | C | 52 | 1 | 45 | 8 | 1.1393e-04 | 8.7885e-04 |
| 3279.snp | 29765 | 77114 | A | T | 709 | 22 | 706 | 25 | 8.5655e-15 | 1.0631e-13 |
| 3280.snp | 29765 | 77236 | C | T | 660 | 22 | 661 | 220 | 6.6874e-12 | 7.6959e-11 |
| 3283.snp | 29765 | 84086 | G | A | 222 | 10 | 293 | 7 | 8.3955e-14 | 1.0154e-12 |
| 3287.snp | 29765 | 106691 | C | A | 36 | 1 | 39 | 5 | 1.6624e-05 | 1.4111e-04 |
| 3295.snp | 29949 | 30833 | T | C | 157 | 9 | 117 | 40 | 4.8861e-03 | 2.7540e-02 |
| 3300.snp | 29784 | 30219 | A | T | 78 | 4 | 33 | 5 | 2.8564e-04 | 2.0741e-03 |
| 3305.snp | 29784 | 79822 | G | C | 19 | 1 | 20 | 3 | 3.2917e-03 | 1.9738e-02 |
| 3311.snp | 29784 | 89008 | A | G | 24 | 2 | 66 | 9 | 5.4543e-05 | 4.3568e-04 |
| 3312.snp | 29784 | 142442 | A | G | 47 | 4 | 58 | 6 | 3.2396e-08 | 3.3369e-07 |
| 3313.snp | 29784 | 144304 | T | A | 112 | 3 | 77 | 5 | 2.2389e-06 | 2.0579e-05 |
| 3374.snp | 27914 | 110136 | A | C | 17 | 0 | 19 | 2 | 1.4880e-03 | 9.4747e-03 |
| 3375.snp | 27914 | 133718 | G | A | 71 | 3 | 96 | 14 | 1.2372e-10 | 1.3774e-09 |
| 3425.snp | 28748 | 89177 | G | A | 101 | 0 | 35 | 0 | 1.8866e-05 | 1.5872e-04 |
| 3427.snp | 29966 | 56452 | G | A | 23 | 0 | 33 | 7 | 4.0851e-03 | 2.3554e-02 |
| 3430.snp | 30205 | 56246 | A | T | 48 | 0 | 36 | 0 | 1.6043e-09 | 1.7341e-08 |
| 3431.snp | 30205 | 226828 | G | A | 65 | 4 | 38 | 5 | 1.6624e-05 | 1.4097e-04 |
| 3433.snp | 30205 | 257355 | C | G | 124 | 39 | 157 | 51 | 4.9720e-04 | 3.4630e-03 |
| 3435.snp | 30205 | 461717 | G | C | 294 | 47 | 190 | 62 | 7.1367e-05 | 5.6114e-04 |
| 3436.snp | 30205 | 467276 | T | G | 146 | 8 | 232 | 41 | 1.3468e-18 | 1.7372e-17 |
| 3437.snp | 30205 | 585715 | G | C | 40 | 3 | 119 | 24 | 7.6818e-08 | 7.7352e-07 |
| 3439.snp | 30205 | 751781 | C | A | 180 | 8 | 40 | 8 | 5.8177e-04 | 3.9870e-03 |
| 3444.snp | 27894 | 124968 | A | G | 1 | 0 | 1 | 0 | 0 | 0 |
| 3543.snp | 27956 | 120958 | A | T | 16 | 0 | 101 | 7 | 4.2811e-03 | 2.4436e-02 |
| 3713.snp | 29846 | 104370 | T | C | 12 | 1 | 10 | 0 | 4.9439e-03 | 2.7810e-02 |
| 3738.snp | 30148 | 245095 | A | G | 87 | 4 | 35 | 8 | 5.7031e-03 | 3.1541e-02 |
| 3749.snp | 30148 | 450584 | G | A | 10 | 0 | 11 | 0 | 9.7656e-04 | 6.4443e-03 |
| 3792.snp | 30128 | 41815 | T | C | 148 | 2 | 140 | 5 | 8.2231e-12 | 9.4505e-11 |
| 3793.snp | 29682 | 80931 | A | C | 21 | 0 | 19 | 1 | 1.3734e-04 | 1.0499e-03 |
| 3799.snp | 29682 | 201439 | C | A | 18 | 1 | 31 | 5 | 7.0777e-04 | 4.8157e-03 |
| 3800.snp | 29682 | 202533 | C | T | 58 | 1 | 42 | 9 | 8.6724e-04 | 5.7900e-03 |
| 3802.snp | 29682 | 214949 | T | C | 107 | 6 | 95 | 2 | 1.9825e-04 | 1.4733e-03 |
| 3803.snp | 29682 | 215238 | T | C | 389 | 58 | 262 | 93 | 1.2397e-04 | 9.5537e-04 |
| 3805.snp | 29682 | 218125 | C | T | 11 | 0 | 114 | 26 | 9.7656e-04 | 6.4393e-03 |
| 3820.snp | 29783 | 161090 | A | G | 134 | 19 | 120 | 42 | 5.7182e-03 | 3.1482e-02 |
| 3821.snp | 29783 | 161316 | A | C | 112 | 6 | 121 | 40 | 2.1201e-03 | 1.2968e-02 |
| 3838.snp | 29629 | 54528 | T | A | 10 | 0 | 10 | 0 | 0 | 0 |
| 3901.snp | 29709 | 14378 | T | C | 140 | 16 | 243 | 81 | 2.3720e-05 | 1.9741e-04 |
| 3902.snp | 29913 | 14479 | C | A | 923 | 113 | 1157 | 414 | 3.3467e-14 | 4.0767e-13 |
| 3905.snp | 29709 | 32778 | G | T | 175 | 18 | 111 | 38 | 6.2372e-03 | 3.3709e-02 |
| 3927.snp | 29709 | 156562 | T | C | 188 | 5 | 54 | 2 | 8.9781e-05 | 6.9949e-04 |
| 3941.snp | 28524 | 3145 | C | T | 81 | 3 | 37 | 1 | 8.3862e-04 | 5.6121e-03 |
| 3943.snp | 28524 | 7075 | C | T | 52 | 4 | 54 | 3 | 7.5807e-04 | 5.1252e-03 |
| 3952.snp | 60625 | 55604 | G | A | 65 | 12 | 36 | 5 | 4.3703e-05 | 3.5272e-04 |
| 4057.snp | 30170 | 769539 | C | T | 12 | 1 | 1 | 0 | 4.9439e-03 | 2.7774e-02 |
| 4124.snp | 30170 | 1844205 | G | C | 178 | 27 | 100 | 34 | 6.7329e-03 | 3.6068e-02 |
| 4126.snp | 30170 | 1851577 | G | A | 32 | 3 | 31 | 6 | 2.7541e-03 | 1.6667e-02 |
| 4129.snp | 30170 | 1866337 | T | G | 48 | 1 | 62 | 10 | 8.8486e-07 | 8.4423e-06 |
| 4134.snp | 30170 | 2101685 | G | A | 318 | 39 | 182 | 55 | 8.1647e-06 | 7.1433e-05 |
| 4136.snp | 30170 | 2140036 | A | G | 28 | 2 | 20 | 3 | 3.2917e-03 | 1.9725e-02 |
| 4146.snp | 30170 | 2418995 | C | G | 1125 | 20 | 939 | 37 | 8.7243e-07 | 8.3330e-06 |
| 4148.snp | 30170 | 2419782 | G | A | 194 | 10 | 349 | 75 | 4.8863e-20 | 6.3701e-19 |
| 4149.snp | 30170 | 2431623 | T | C | 165 | 8 | 99 | 10 | 1.6734e-06 | 1.5634e-05 |
| 4154.snp | 30170 | 2786225 | A | G | 93 | 2 | 44 | 6 | 5.3625e-06 | 4.7748e-05 |
| 4157.snp | 30170 | 2806400 | C | T | 39 | 1 | 16 | 1 | 4.6156e-04 | 3.2412e-03 |
| 4160.snp | 30170 | 2852038 | C | T | 102 | 8 | 253 | 96 | 1.9625e-03 | 1.2047e-02 |
| 4163.snp | 30170 | 3142383 | T | C | 159 | 9 | 77 | 9 | 1.4228e-04 | 1.0780e-03 |
| 4169.snp | 30170 | 3260920 | C | T | 49 | 1 | 89 | 17 | 2.4710e-07 | 2.4280e-06 |
| 4181.snp | 30170 | 3637590 | T | C | 375 | 19 | 296 | 112 | 6.5890e-04 | 4.4975e-03 |
| 4182.snp | 30170 | 3685567 | G | C | 45 | 3 | 22 | 3 | 1.2359e-03 | 7.9831e-03 |
| 4185.snp | 30170 | 3707608 | G | A | 84 | 0 | 59 | 0 | 1.8697e-05 | 1.5807e-04 |
| 4187.snp | 30170 | 3874616 | T | C | 10 | 0 | 36 | 0 | 3.9062e-03 | 2.2925e-02 |
| 4188.snp | 30170 | 3883191 | T | G | 29 | 2 | 44 | 10 | 1.2308e-03 | 7.9677e-03 |
| 4189.snp | 30170 | 3888230 | A | G | 21 | 0 | 40 | 4 | 9.5367e-07 | 9.0886e-06 |
| 4190.snp | 30170 | 3972456 | A | G | 1182 | 75 | 978 | 29 | 2.0167e-24 | 2.7162e-23 |
| 4194.snp | 30170 | 4029489 | G | A | 6262 | 221 | 4393 | 1597 | 3.7617e-44 | 5.3180e-43 |
| 4195.snp | 30170 | 4029629 | G | C | 8906 | 579 | 7205 | 240 | 3.4801e-107 | 5.1147e-106 |
| 4204.snp | 30170 | 4157706 | T | C | 24 | 3 | 40 | 8 | 5.8177e-04 | 3.9838e-03 |
| 4210.snp | 30170 | 4406026 | T | C | 52 | 6 | 104 | 32 | 8.6766e-04 | 5.7882e-03 |
| 4223.snp | 30170 | 4492531 | G | C | 111 | 36 | 251 | 100 | 9.2354e-03 | 4.7911e-02 |
| 4224.snp | 30170 | 4503927 | G | T | 337 | 15 | 192 | 64 | 1.3081e-04 | 1.0063e-03 |
| 4225.snp | 30170 | 4507094 | A | G | 69 | 2 | 82 | 17 | 2.3938e-06 | 2.1885e-05 |
| 4227.snp | 30170 | 4533650 | A | G | 46 | 0 | 83 | 0 | 7.1310e-06 | 6.2582e-05 |
| 4232.snp | 30170 | 4596539 | A | G | 29 | 3 | 32 | 7 | 4.0851e-03 | 2.3506e-02 |
| 4309.snp | 28489 | 8785 | A | G | 74 | 10 | 87 | 27 | 3.2279e-03 | 1.9397e-02 |
| 4311.snp | 30143 | 162842 | T | C | 11 | 0 | 78 | 10 | 9.7656e-04 | 6.4343e-03 |
| 4313.snp | 30143 | 167025 | T | G | 101 | 5 | 144 | 30 | 7.3853e-10 | 8.0541e-09 |
| 4314.snp | 30143 | 167462 | A | T | 61 | 5 | 92 | 8 | 1.6675e-10 | 1.8540e-09 |
| 4317.snp | 30143 | 271572 | C | T | 141 | 23 | 150 | 54 | 4.2362e-03 | 2.4245e-02 |
| 4382.snp | 29814 | 39690 | T | C | 43 | 2 | 54 | 12 | 2.7519e-04 | 2.0050e-03 |
| 4451.snp | 29648 | 72594 | A | T | 10 | 0 | 14 | 0 | 1.5259e-03 | 9.6446e-03 |
| 4476.snp | 30125 | 646434 | T | C | 73 | 2 | 118 | 5 | 1.7900e-04 | 1.3396e-03 |
| 4480.snp | 29812 | 110744 | A | T | 177 | 27 | 313 | 53 | 4.1461e-17 | 5.2605e-16 |
| 4487.snp | 29815 | 49070 | G | T | 1369 | 559 | 1602 | 723 | 2.0930e-03 | 1.2811e-02 |
| 4523.snp | 29788 | 73294 | T | C | 732 | 233 | 659 | 182 | 2.4927e-15 | 3.1119e-14 |
| 4535.snp | 29005 | 58544 | G | A | 11 | 0 | 10 | 0 | 3.9062e-03 | 2.2878e-02 |
| 4542.snp | 29758 | 101355 | C | T | 47 | 1 | 22 | 3 | 1.2359e-03 | 7.9771e-03 |
| 4544.snp | 29758 | 110713 | G | T | 19 | 1 | 24 | 5 | 9.2645e-03 | 4.7800e-02 |
| 4569.snp | 30093 | 139687 | T | C | 106 | 5 | 56 | 13 | 3.8103e-04 | 2.7227e-03 |
| 4585.snp | 29634 | 28940 | T | C | 14 | 0 | 10 | 0 | 3.9062e-03 | 2.2862e-02 |
| 4587.snp | 29634 | 111270 | A | T | 24 | 0 | 12 | 0 | 2.4414e-04 | 1.7972e-03 |
| 4600.snp | 29634 | 229139 | A | T | 80 | 9 | 200 | 34 | 3.1518e-11 | 3.5647e-10 |
| 4604.snp | 29634 | 333285 | C | T | 50 | 2 | 137 | 29 | 4.3194e-09 | 4.5875e-08 |
| 4616.snp | 29634 | 409438 | A | C | 49 | 1 | 55 | 4 | 1.8974e-03 | 1.1664e-02 |
| 4620.snp | 29634 | 416274 | A | C | 12 | 1 | 15 | 1 | 4.9439e-03 | 2.7756e-02 |
| 4628.snp | 29634 | 456935 | C | T | 236 | 20 | 308 | 105 | 5.3435e-06 | 4.7628e-05 |
| 4765.snp | 29737 | 331885 | C | T | 32 | 2 | 61 | 8 | 5.3087e-07 | 5.1338e-06 |
| 4823.snp | 30073 | 435885 | G | A | 108 | 23 | 105 | 36 | 7.9782e-03 | 4.2236e-02 |
| 5051.snp | 29908 | 2023760 | G | A | 34 | 2 | 49 | 13 | 5.3219e-03 | 2.9566e-02 |
| 5061.snp | 29948 | 99656 | C | T | 1 | 0 | 17 | 2 | 4.2811e-03 | 2.4371e-02 |
| 5115.snp | 29724 | 681570 | C | A | 45 | 9 | 40 | 9 | 1.8296e-03 | 1.1321e-02 |
| 5273.snp | 60214 | 2114 | C | T | 78 | 8 | 104 | 33 | 1.5883e-03 | 9.9874e-03 |
| 5362.snp | 29898 | 20362 | C | T | 90 | 3 | 133 | 48 | 8.6148e-03 | 4.4937e-02 |
| 5363.snp | 29898 | 30504 | C | T | 74 | 11 | 134 | 29 | 2.0156e-08 | 2.0938e-07 |
| 5383.snp | 29968 | 31196 | G | A | 79 | 1 | 122 | 28 | 1.9177e-07 | 1.9019e-06 |
| 5385.snp | 29968 | 70959 | A | T | 47 | 7 | 26 | 5 | 4.0409e-03 | 2.3377e-02 |
| 5386.snp | 29968 | 109001 | G | A | 54 | 0 | 21 | 0 | 3.2917e-03 | 1.9697e-02 |
| 5389.snp | 29968 | 226146 | G | A | 96 | 17 | 43 | 5 | 2.2913e-06 | 2.1038e-05 |
| 5435.snp | 29830 | 45731 | C | T | 71 | 5 | 79 | 15 | 1.0318e-06 | 9.7574e-06 |
| 5438.snp | 29830 | 83997 | C | T | 20 | 2 | 49 | 10 | 5.0450e-04 | 3.4967e-03 |
| 5446.snp | 29830 | 374905 | C | T | 11 | 0 | 11 | 0 | 3.9062e-03 | 2.2815e-02 |
| 5447.snp | 29830 | 387710 | C | A | 101 | 10 | 130 | 15 | 7.0427e-06 | 6.1870e-05 |
| 5450.snp | 29830 | 446960 | C | A | 85 | 13 | 38 | 6 | 8.7522e-05 | 6.8313e-04 |
| 5463.snp | 29830 | 561988 | A | C | 10 | 0 | 10 | 0 | 0 | 0 |
| 6023.snp | 30147 | 4394375 | G | A | 1051 | 60 | 1934 | 125 | 3.0891e-187 | 4.5637e-186 |
| 6048.snp | 28623 | 76300 | T | C | 711 | 14 | 326 | 123 | 3.2634e-04 | 2.3616e-03 |
| 6054.snp | 28623 | 116965 | C | G | 1695 | 97 | 678 | 27 | 5.6520e-12 | 6.5132e-11 |
| 6059.snp | 28623 | 122855 | G | C | 16 | 0 | 40 | 5 | 5.8177e-04 | 3.9806e-03 |
| 6065.snp | 28623 | 128628 | G | A | 17 | 1 | 32 | 6 | 1.2034e-03 | 7.8084e-03 |
| 6067.snp | 28623 | 129588 | A | T | 87 | 11 | 77 | 23 | 3.1245e-03 | 1.8789e-02 |
| 6069.snp | 28623 | 130450 | G | A | 98 | 6 | 152 | 38 | 1.2320e-07 | 1.2290e-06 |
| 6074.snp | 28693 | 7885 | C | T | 41 | 4 | 18 | 1 | 1.3734e-04 | 1.0480e-03 |
| 6095.snp | 29993 | 126233 | C | T | 143 | 1 | 174 | 3 | 1.1716e-12 | 1.3914e-11 |
| 6096.snp | 29993 | 334536 | T | A | 31 | 3 | 40 | 5 | 1.9581e-05 | 1.6424e-04 |
| 6183.snp | 29790 | 322657 | T | A | 177 | 35 | 145 | 48 | 8.2386e-04 | 5.5263e-03 |
| 6231.snp | 29889 | 294121 | G | A | 10 | 0 | 10 | 0 | 0 | 0 |
| 6237.snp | 29889 | 429051 | G | C | 38 | 0 | 92 | 30 | 4.5629e-03 | 2.5769e-02 |
| 6305.snp | 29656 | 156872 | T | C | 10 | 0 | 10 | 0 | 0 | 0 |
| 6334.snp | 29630 | 107215 | G | A | 26 | 0 | 58 | 1 | 1.8697e-05 | 1.5792e-04 |
| 6335.snp | 29630 | 112722 | G | C | 30 | 1 | 25 | 5 | 9.2645e-03 | 4.7714e-02 |
| 6344.snp | 29630 | 368039 | C | T | 10 | 0 | 11 | 0 | 3.9062e-03 | 2.2768e-02 |
| 6424.snp | 29048 | 13949 | A | T | 10 | 0 | 10 | 0 | 0 | 0 |
| 6428.snp | 30171 | 214323 | C | A | 18 | 0 | 10 | 0 | 3.9062e-03 | 2.2753e-02 |
| 6434.snp | 29615 | 232301 | A | T | 43 | 10 | 29 | 6 | 6.1394e-03 | 3.3391e-02 |
| 6478.snp | 28833 | 46875 | T | C | 20 | 0 | 12 | 0 | 3.9062e-03 | 2.2737e-02 |
| 6482.snp | 29651 | 67140 | A | G | 56 | 1 | 79 | 23 | 1.8453e-03 | 1.1393e-02 |
| 6500.snp | 29916 | 141809 | G | T | 212 | 21 | 178 | 48 | 1.9411e-07 | 1.9229e-06 |
| 6502.snp | 29916 | 165488 | G | T | 1321 | 16 | 321 | 6 | 6.3161e-21 | 8.2720e-20 |
| 6505.snp | 30152 | 61725 | A | C | 38 | 2 | 85 | 19 | 9.9610e-06 | 8.6793e-05 |
| 6506.snp | 30152 | 61913 | C | T | 165 | 13 | 220 | 84 | 3.8863e-03 | 2.2981e-02 |
| 6516.snp | 30152 | 874442 | G | A | 27 | 1 | 17 | 0 | 4.2811e-03 | 2.4306e-02 |
| 6526.snp | 30152 | 1611292 | T | C | 50 | 4 | 71 | 22 | 7.5601e-03 | 4.0297e-02 |
| 6527.snp | 30152 | 1612269 | A | G | 68 | 8 | 71 | 19 | 9.0174e-04 | 6.0062e-03 |
| 6576.snp | 29806 | 62906 | A | C | 55 | 3 | 80 | 16 | 1.5967e-06 | 1.4966e-05 |
| 6578.snp | 29806 | 89839 | T | C | 419 | 25 | 213 | 54 | 1.2819e-09 | 1.3891e-08 |
| 6593.snp | 29646 | 233209 | G | T | 4454 | 2029 | 6478 | 2886 | 5.7792e-06 | 5.1139e-05 |
| 6640.snp | 29840 | 416315 | G | A | 80 | 5 | 30 | 6 | 2.7541e-03 | 1.6655e-02 |
| 6656.snp | 28331 | 5538 | T | G | 40 | 7 | 36 | 7 | 8.3862e-04 | 5.6077e-03 |
| 6681.snp | 29863 | 174316 | A | C | 10 | 0 | 10 | 0 | 0 | 0 |
| 6724.snp | 28772 | 13307 | C | A | 35 | 4 | 42 | 11 | 6.4229e-03 | 3.4646e-02 |
| 6738.snp | 29279 | 26382 | C | T | 143 | 0 | 112 | 0 | 5.3926e-05 | 4.3196e-04 |
| 6741.snp | 29279 | 64237 | G | C | 28 | 4 | 18 | 3 | 8.5165e-03 | 4.4560e-02 |
| 6758.snp | 29670 | 49129 | C | A | 739 | 115 | 438 | 155 | 8.5616e-07 | 8.1867e-06 |
| 6760.snp | 29670 | 53240 | A | G | 162 | 33 | 263 | 84 | 1.7676e-06 | 1.6441e-05 |
| 6761.snp | 29670 | 53404 | G | A | 1464 | 12 | 1818 | 47 | 8.6228e-65 | 1.2480e-63 |
| 6797.snp | 29776 | 201158 | G | T | 244 | 2 | 251 | 90 | 2.9046e-04 | 2.1055e-03 |
| 6798.snp | 29776 | 201258 | C | G | 246 | 14 | 242 | 86 | 2.2596e-04 | 1.6749e-03 |
| 6891.snp | 30184 | 600811 | G | A | 97 | 3 | 95 | 32 | 8.6326e-03 | 4.5003e-02 |
| 6932.snp | 29751 | 63734 | A | T | 229 | 10 | 195 | 8 | 2.9050e-06 | 2.6417e-05 |
| 6933.snp | 29751 | 65835 | G | C | 77 | 2 | 136 | 4 | 4.9573e-05 | 3.9822e-04 |
| 6955.snp | 29751 | 452677 | T | C | 68 | 1 | 223 | 56 | 3.4741e-10 | 3.8527e-09 |
| 6956.snp | 29751 | 452743 | G | A | 77 | 2 | 260 | 64 | 3.3485e-12 | 3.9008e-11 |
| 6957.snp | 29751 | 452833 | C | T | 41 | 1 | 140 | 39 | 8.6648e-06 | 7.5576e-05 |
| 6965.snp | 29751 | 572952 | A | G | 86 | 6 | 50 | 1 | 4.0296e-04 | 2.8650e-03 |
| 6967.snp | 29751 | 639030 | A | G | 78 | 5 | 102 | 16 | 1.8791e-05 | 1.5824e-04 |
| 6995.snp | 30190 | 141645 | T | C | 179 | 6 | 107 | 19 | 2.5627e-09 | 2.7560e-08 |
| 7001.snp | 30190 | 308340 | C | T | 51 | 3 | 36 | 0 | 2.7289e-03 | 1.6538e-02 |
| 7006.snp | 30190 | 394547 | T | G | 10 | 0 | 17 | 2 | 4.2811e-03 | 2.4290e-02 |
| 7009.snp | 30190 | 458170 | C | T | 91 | 8 | 34 | 5 | 1.1283e-04 | 8.7109e-04 |
| 7013.snp | 30190 | 792244 | G | C | 131 | 0 | 98 | 0 | 1.1372e-05 | 9.8282e-05 |
| 7014.snp | 30190 | 793033 | C | T | 206 | 3 | 63 | 12 | 1.3380e-05 | 1.1482e-04 |
| 7015.snp | 30190 | 812578 | G | A | 10 | 0 | 10 | 0 | 0 | 0 |
| 7026.snp | 30190 | 1158725 | C | T | 4901 | 502 | 6942 | 1959 | 8.4381e-189 | 1.2487e-187 |
| 7027.snp | 30190 | 1159189 | G | T | 1043 | 158 | 2054 | 303 | 6.5818e-92 | 9.6401e-91 |
| 7028.snp | 30190 | 1161935 | G | A | 1081 | 129 | 1579 | 163 | 1.2130e-09 | 1.3161e-08 |
| 7029.snp | 30190 | 1170708 | A | C | 281 | 20 | 186 | 29 | 1.1510e-17 | 1.4692e-16 |
| 7030.snp | 30190 | 1211628 | G | T | 408 | 18 | 550 | 73 | 4.4823e-57 | 6.4219e-56 |
| 7032.snp | 30190 | 1264184 | T | C | 343 | 115 | 549 | 236 | 8.5863e-03 | 4.4815e-02 |
| 7033.snp | 30190 | 1266924 | G | A | 322 | 13 | 711 | 19 | 1.2374e-24 | 1.6746e-23 |
| 7035.snp | 30190 | 1284925 | C | A | 29 | 6 | 56 | 8 | 6.1394e-03 | 3.3370e-02 |
| 7036.snp | 30190 | 1293727 | T | G | 53 | 0 | 25 | 3 | 4.5245e-04 | 3.1877e-03 |
| 7045.snp | 30190 | 2197798 | T | A | 93 | 13 | 199 | 51 | 6.7452e-09 | 7.1020e-08 |
| 7046.snp | 30190 | 2310582 | C | T | 10 | 0 | 10 | 0 | 3.9062e-03 | 2.2706e-02 |
| 7048.snp | 30190 | 2311363 | C | T | 114 | 10 | 90 | 14 | 1.3285e-04 | 1.0211e-03 |
| 7060.snp | 30190 | 2501115 | G | A | 326 | 67 | 477 | 172 | 1.2243e-06 | 1.1539e-05 |
| 7061.snp | 30190 | 2520994 | A | C | 22 | 1 | 28 | 2 | 1.1683e-05 | 1.0066e-04 |
| 7064.snp | 30190 | 2527863 | T | A | 62 | 12 | 60 | 18 | 7.2988e-03 | 3.9002e-02 |
| 7067.snp | 30190 | 2773241 | C | T | 48 | 2 | 34 | 3 | 2.2189e-06 | 2.0439e-05 |
| 7074.snp | 30190 | 2806483 | C | T | 220 | 19 | 208 | 10 | 4.0052e-06 | 3.5887e-05 |
| 7082.snp | 30190 | 2825370 | C | T | 74 | 19 | 62 | 19 | 8.4484e-03 | 4.4449e-02 |
| 7093.snp | 30190 | 3033773 | T | C | 16 | 0 | 16 | 0 | 4.2811e-03 | 2.4274e-02 |
| 7096.snp | 30190 | 3052653 | G | A | 32 | 1 | 50 | 12 | 1.1257e-03 | 7.3267e-03 |
| 7097.snp | 30190 | 3053266 | G | C | 30 | 3 | 50 | 1 | 1.9581e-05 | 1.6408e-04 |
| 7105.snp | 30190 | 3143461 | G | C | 49 | 2 | 89 | 17 | 4.9845e-06 | 4.4475e-05 |
| 7107.snp | 30190 | 3150977 | G | T | 666 | 75 | 177 | 62 | 1.1568e-03 | 7.5228e-03 |
| 7108.snp | 30190 | 3152943 | C | T | 42 | 0 | 79 | 9 | 8.5812e-11 | 9.6036e-10 |
| 7109.snp | 30190 | 3200122 | A | G | 267 | 12 | 459 | 72 | 4.3293e-40 | 6.0603e-39 |
| 7110.snp | 30190 | 3211000 | G | C | 31 | 1 | 14 | 1 | 1.5259e-03 | 9.6303e-03 |
| 7121.snp | 30190 | 3446225 | T | A | 24 | 0 | 10 | 0 | 3.9062e-03 | 2.2691e-02 |
| 7125.snp | 30190 | 3556425 | G | T | 346 | 4 | 711 | 10 | 1.4977e-64 | 2.1639e-63 |
| 7128.snp | 30190 | 3654558 | C | T | 113 | 3 | 104 | 20 | 2.0062e-08 | 2.0866e-07 |
| 7198.snp | 29628 | 141776 | C | T | 131 | 11 | 37 | 7 | 8.3862e-04 | 5.6033e-03 |
| 7233.snp | 29816 | 119480 | A | C | 71 | 4 | 87 | 17 | 5.3275e-07 | 5.1461e-06 |
| 7236.snp | 29816 | 188430 | C | T | 40 | 0 | 31 | 2 | 1.7221e-06 | 1.6054e-05 |
| 7237.snp | 29816 | 198420 | G | A | 187 | 6 | 78 | 1 | 3.3915e-06 | 3.0678e-05 |
| 7238.snp | 29816 | 224144 | A | G | 23 | 3 | 23 | 4 | 5.9002e-03 | 3.2296e-02 |
| 7240.snp | 29816 | 309164 | T | C | 55 | 11 | 119 | 35 | 1.7900e-04 | 1.3384e-03 |
| 7241.snp | 29816 | 309261 | C | G | 120 | 7 | 182 | 50 | 2.8491e-07 | 2.7868e-06 |
| 7248.snp | 28176 | 61265 | T | C | 51 | 6 | 61 | 17 | 3.5359e-03 | 2.1026e-02 |
| 7250.snp | 30055 | 142957 | G | T | 152 | 56 | 155 | 56 | 6.8048e-03 | 3.6407e-02 |
| 7262.snp | 28883 | 69386 | C | A | 234 | 23 | 133 | 30 | 4.7922e-08 | 4.9006e-07 |
| 7343.snp | 29308 | 28740 | G | A | 290 | 107 | 208 | 25 | 2.7962e-04 | 2.0321e-03 |
| 7348.snp | 29950 | 52262 | A | G | 10 | 0 | 164 | 0 | 9.7656e-04 | 6.3899e-03 |
| 7438.snp | 28611 | 37884 | A | T | 184 | 4 | 105 | 2 | 6.5787e-08 | 6.6480e-07 |
| 7439.snp | 28611 | 38402 | T | C | 42 | 3 | 28 | 3 | 5.6818e-05 | 4.5301e-04 |
| 7441.snp | 28611 | 39383 | C | T | 73 | 4 | 56 | 11 | 4.1301e-05 | 3.3524e-04 |
| 7447.snp | 28611 | 49660 | G | A | 36 | 5 | 70 | 13 | 4.3703e-05 | 3.5205e-04 |
| 7472.snp | 29900 | 192020 | T | G | 93 | 9 | 77 | 22 | 1.5695e-03 | 9.8835e-03 |
| 7473.snp | 29900 | 252651 | A | G | 30 | 3 | 32 | 6 | 1.2034e-03 | 7.8025e-03 |
| 7476.snp | 29900 | 329883 | A | T | 34 | 1 | 70 | 5 | 1.1972e-07 | 1.1956e-06 |
| 7478.snp | 29900 | 429067 | T | C | 257 | 11 | 143 | 5 | 1.9829e-07 | 1.9620e-06 |
| 7500.snp | 30076 | 1116400 | C | A | 10 | 0 | 10 | 0 | 0 | 0 |
| 7517.snp | 29736 | 97153 | C | G | 130 | 8 | 26 | 5 | 4.0409e-03 | 2.3362e-02 |
| 7520.snp | 29736 | 190929 | G | C | 578 | 11 | 524 | 24 | 5.2009e-05 | 4.1700e-04 |
| 7527.snp | 29736 | 244174 | G | A | 370 | 31 | 188 | 60 | 3.7612e-05 | 3.0734e-04 |
| 7563.snp | 29827 | 126430 | G | A | 10 | 0 | 13 | 0 | 0 | 0 |
| 7589.snp | 29827 | 310979 | C | A | 19 | 3 | 24 | 5 | 9.2645e-03 | 4.7656e-02 |
| 7603.snp | 29827 | 497630 | A | T | 16 | 1 | 20 | 3 | 3.2917e-03 | 1.9656e-02 |
| 7610.snp | 29827 | 599357 | C | G | 26 | 5 | 18 | 2 | 4.0409e-03 | 2.3346e-02 |
| 7612.snp | 29827 | 611636 | A | C | 35 | 1 | 31 | 6 | 2.7541e-03 | 1.6643e-02 |
| 7619.snp | 29827 | 641903 | A | C | 52 | 2 | 75 | 17 | 4.1677e-05 | 3.3701e-04 |
| 7621.snp | 29827 | 647152 | A | T | 21 | 0 | 15 | 1 | 1.5259e-03 | 9.6161e-03 |
| 7623.snp | 29827 | 656505 | G | A | 82 | 1 | 55 | 16 | 9.2433e-03 | 4.7893e-02 |
| 7624.snp | 29827 | 666785 | A | C | 19 | 0 | 22 | 3 | 1.2359e-03 | 7.9651e-03 |
| 7626.snp | 29827 | 680767 | A | T | 12 | 0 | 109 | 29 | 2.4414e-04 | 1.7879e-03 |
| 7627.snp | 29827 | 681534 | G | A | 78 | 1 | 281 | 39 | 1.4592e-21 | 1.9347e-20 |
| 7628.snp | 29827 | 701020 | G | T | 254 | 42 | 139 | 16 | 1.5381e-06 | 1.4433e-05 |
| 7629.snp | 29827 | 736408 | T | G | 38 | 1 | 46 | 7 | 1.1068e-05 | 9.5951e-05 |
| 7631.snp | 29827 | 793885 | C | T | 113 | 5 | 71 | 10 | 2.4467e-08 | 2.5355e-07 |
| 7674.snp | 27942 | 37838 | C | T | 201 | 6 | 84 | 2 | 1.5632e-04 | 1.1792e-03 |
| 7679.snp | 29633 | 2534 | T | G | 102 | 15 | 840 | 229 | 4.1720e-11 | 4.6998e-10 |
| 7680.snp | 29633 | 2606 | A | C | 1764 | 87 | 2270 | 552 | 5.9112e-90 | 8.6431e-89 |
| 7681.snp | 29633 | 2804 | A | G | 1665 | 15 | 3634 | 77 | 6.9289e-193 | 1.0272e-191 |
| 7682.snp | 29633 | 3223 | T | C | 344 | 126 | 1397 | 385 | 5.9346e-05 | 4.7140e-04 |
| 7683.snp | 29633 | 8807 | T | C | 203 | 14 | 147 | 9 | 1.6323e-06 | 1.5283e-05 |
| 7684.snp | 29822 | 9522 | A | G | 49 | 2 | 70 | 14 | 6.7714e-06 | 5.9548e-05 |
| 7686.snp | 29633 | 219166 | C | A | 127 | 4 | 79 | 3 | 7.2114e-08 | 7.2787e-07 |
| 7748.snp | 29609 | 138931 | C | T | 73 | 15 | 214 | 66 | 1.0146e-05 | 8.8136e-05 |
| 7763.snp | 29609 | 272448 | A | G | 47 | 0 | 43 | 7 | 6.6098e-05 | 5.2068e-04 |
| 7765.snp | 29593 | 249051 | A | G | 63 | 6 | 71 | 22 | 7.5601e-03 | 4.0272e-02 |
| 7788.snp | 28582 | 117726 | A | T | 10 | 0 | 12 | 0 | 2.4414e-04 | 1.7849e-03 |
| 7806.snp | 29914 | 49507 | G | T | 35 | 2 | 65 | 8 | 1.6197e-07 | 1.6119e-06 |
| 7860.snp | 29453 | 26872 | A | G | 46 | 2 | 86 | 1 | 4.2569e-03 | 2.4347e-02 |
| 7907.snp | 27721 | 15688 | T | C | 77 | 2 | 56 | 16 | 5.2496e-03 | 2.9241e-02 |
| 7911.snp | 30179 | 104268 | T | C | 23 | 0 | 68 | 0 | 7.2943e-04 | 4.9551e-03 |
| 7934.snp | 29852 | 217127 | C | T | 515 | 115 | 588 | 251 | 4.3081e-03 | 2.4394e-02 |
| 7941.snp | 29852 | 910139 | T | A | 88 | 5 | 115 | 35 | 4.8484e-04 | 3.3851e-03 |
| 7972.snp | 28738 | 106857 | T | C | 110 | 4 | 63 | 15 | 3.4685e-04 | 2.5015e-03 |
| 7973.snp | 28738 | 106954 | G | T | 162 | 3 | 131 | 21 | 1.9884e-12 | 2.3387e-11 |
| 7984.snp | 29601 | 33469 | T | C | 558 | 49 | 1343 | 482 | 6.0718e-16 | 7.5911e-15 |
| 7990.snp | 29601 | 136216 | C | A | 518 | 35 | 467 | 187 | 6.1762e-04 | 4.2191e-03 |
| 8143.snp | 30028 | 146524 | C | T | 204 | 28 | 110 | 29 | 2.0355e-05 | 1.7007e-04 |
| 8144.snp | 30028 | 148556 | G | A | 320 | 1 | 166 | 53 | 1.0229e-04 | 7.9335e-04 |
| 8167.snp | 29915 | 115606 | A | T | 1338 | 166 | 1046 | 395 | 5.5187e-10 | 6.0571e-09 |
| 8170.snp | 29915 | 129872 | T | A | 184 | 9 | 70 | 20 | 1.9401e-03 | 1.1919e-02 |
| 8171.snp | 29915 | 188972 | T | C | 31 | 2 | 42 | 11 | 6.4229e-03 | 3.4624e-02 |
| 8174.snp | 27916 | 16351 | C | G | 41 | 1 | 29 | 6 | 6.1394e-03 | 3.3306e-02 |
| 8183.snp | 30059 | 93493 | T | C | 147 | 9 | 123 | 2 | 5.6330e-11 | 6.3289e-10 |
| 8184.snp | 30059 | 93650 | G | A | 171 | 1 | 85 | 0 | 5.3776e-09 | 5.6831e-08 |
| 8268.snp | 29994 | 123516 | A | C | 10 | 0 | 14 | 0 | 9.7656e-04 | 6.3850e-03 |
| 8283.snp | 29740 | 81607 | C | T | 15 | 1 | 35 | 8 | 5.7031e-03 | 3.1480e-02 |
| 8324.snp | 29848 | 173284 | C | T | 10 | 0 | 10 | 0 | 0 | 0 |
| 8455.snp | 29841 | 426103 | A | G | 10 | 0 | 10 | 0 | 0 | 0 |
| 8510.snp | 27967 | 18159 | A | C | 241 | 12 | 205 | 76 | 2.6700e-03 | 1.6204e-02 |
