# Supplementary material for: Genomic imprinting, methylation and parent-of-origin effects in reciprocal hybrid endosperm of castor bean
Source: Nucleic Acids Res. 2014 May 5;42(11):6987–98. doi: 10.1093/nar/gku375 (PMC4066788; doi:10.1093/nar/gku375)
Supplement: SUPPLEMENTARY DATA [file supp_gku375_nar-00469-v-2014-File009.zip › Supplementary_Table_S4.docx]

| **Supplementary Table S4.** Genes with paternally-biased expression (PEGs, FDR<0.05) in both hybrid endosperms. m, Maternal alleles; p, Paternal alleles. | | | | | | | | | | |
| --- | --- | --- | --- | --- | --- | --- | --- | --- | --- | --- |
| **SNP No.** | **Scaffold** | **Position** | **SNP_ZB107** | **SNP_ZB306** | **ZB107×ZB306** | | **ZB306×ZB107** | | **p-value** | **FDR** |
|  |  |  |  |  | **m_ZB107** | **p_ZB306** | **m_ZB306** | **p_ZB107** |  |  |
| 1133.snp | 30138 | 1075178 | G | A | 3 | 44 | 1 | 45 | 9.7656e-04 | 6.4793e-03 |
| 1140.snp | 30138 | 1086643 | C | A | 289 | 211 | 87 | 64 | 1.8324e-03 | 1.1322e-02 |
| 1257.snp | 27985 | 280748 | T | G | 1016 | 652 | 849 | 539 | 4.3643e-08 | 4.4738e-07 |
| 1329.snp | 30078 | 612217 | G | C | 62 | 46 | 40 | 32 | 9.6769e-03 | 4.9688e-02 |
| 1369.snp | 29883 | 207657 | C | T | 63 | 55 | 22 | 50 | 9.3571e-05 | 7.2703e-04 |
| 1617.snp | 29813 | 255499 | A | G | 21 | 20 | 24 | 26 | 5.0064e-03 | 2.8014e-02 |
| 1792.snp | 29726 | 1020657 | A | T | 35 | 29 | 83 | 66 | 6.2317e-03 | 3.3786e-02 |
| 1794.snp | 29726 | 1032668 | A | T | 109 | 100 | 80 | 61 | 1.3740e-03 | 8.7751e-03 |
| 1814.snp | 29726 | 1110783 | C | T | 144 | 112 | 55 | 63 | 1.0022e-05 | 8.7149e-05 |
| 1820.snp | 29726 | 1124531 | T | C | 164 | 133 | 62 | 63 | 8.5496e-07 | 8.1844e-06 |
| 1996.snp | 30010 | 202029 | C | T | 60 | 51 | 87 | 62 | 4.1556e-03 | 2.3847e-02 |
| 2033.snp | 30131 | 228139 | C | T | 125 | 112 | 167 | 122 | 4.1506e-05 | 3.3626e-04 |
| 2097.snp | 30131 | 1977629 | C | T | 49 | 43 | 118 | 101 | 4.2136e-04 | 2.9884e-03 |
| 2166.snp | 28193 | 134420 | G | C | 98 | 70 | 45 | 36 | 4.5488e-03 | 2.5706e-02 |
| 2198.snp | 29773 | 138720 | T | G | 36 | 31 | 58 | 64 | 4.2132e-03 | 2.4161e-02 |
| 2212.snp | 30074 | 555391 | G | A | 21 | 800 | 29 | 707 | 0 | 0 |
| 2214.snp | 30074 | 557554 | T | C | 9 | 61 | 14 | 189 | 4.3410e-28 | 5.9594e-27 |
| 2215.snp | 30074 | 559897 | A | G | 7 | 43 | 2 | 59 | 6.0748e-20 | 7.9074e-19 |
| 2279.snp | 30060 | 446437 | A | G | 41 | 38 | 73 | 72 | 3.3625e-04 | 2.4312e-03 |
| 236.snp | 28166 | 348765 | G | A | 589 | 422 | 1558 | 983 | 2.7123e-12 | 3.1640e-11 |
| 237.snp | 28166 | 356035 | T | C | 64333 | 40633 | 72793 | 40170 | 5.1171e-84 | 7.4438e-83 |
| 241.snp | 28166 | 446219 | C | T | 1135 | 779 | 596 | 501 | 5.0736e-17 | 6.4278e-16 |
| 244.snp | 28166 | 531739 | C | T | 1 | 55 | 2 | 38 | 5.5620e-21 | 7.3181e-20 |
| 245.snp | 28166 | 533958 | T | A | 7 | 95 | 1 | 66 | 1.8368e-40 | 2.5797e-39 |
| 2770.snp | 29929 | 127302 | G | A | 1467 | 902 | 1549 | 852 | 2.8207e-03 | 1.7010e-02 |
| 2771.snp | 29929 | 129684 | C | T | 2443 | 1664 | 3800 | 2328 | 2.0140e-21 | 2.6581e-20 |
| 2879.snp | 29929 | 685697 | T | C | 388 | 313 | 577 | 391 | 5.7605e-09 | 6.0802e-08 |
| 2880.snp | 29929 | 685855 | C | T | 1493 | 1029 | 1922 | 1088 | 2.5200e-05 | 2.0912e-04 |
| 3114.snp | 29333 | 216956 | G | A | 87 | 80 | 52 | 48 | 1.1198e-04 | 8.6690e-04 |
| 3197.snp | 28226 | 173495 | C | T | 126 | 89 | 143 | 93 | 7.4753e-03 | 3.9870e-02 |
| 3256.snp | 30053 | 31238 | G | A | 147 | 111 | 122 | 83 | 4.5332e-03 | 2.5635e-02 |
| 3337.snp | 29904 | 281222 | T | C | 600 | 359 | 203 | 257 | 4.6535e-04 | 3.2517e-03 |
| 3346.snp | 29904 | 521588 | G | C | 63 | 49 | 46 | 45 | 2.0096e-03 | 1.2319e-02 |
| 3464.snp | 29728 | 123204 | A | G | 632 | 588 | 825 | 565 | 8.9952e-13 | 1.0698e-11 |
| 3522.snp | 29661 | 313207 | T | C | 88 | 69 | 336 | 209 | 1.2126e-03 | 7.8559e-03 |
| 3562.snp | 29489 | 4995 | G | A | 5 | 10 | 61 | 48 | 1.7779e-03 | 1.1049e-02 |
| 359.snp | 29003 | 5303 | T | C | 27 | 24 | 28 | 26 | 5.3219e-03 | 2.9585e-02 |
| 3730.snp | 29686 | 250251 | G | C | 53 | 49 | 88 | 61 | 9.6517e-03 | 4.9588e-02 |
| 3828.snp | 29629 | 36601 | G | C | 57963 | 49497 | 46985 | 55033 | 0 | 0 |
| 3944.snp | 29850 | 107570 | G | A | 463 | 307 | 585 | 370 | 5.8865e-06 | 5.2034e-05 |
| 3957.snp | 29739 | 124446 | G | T | 28 | 25 | 39 | 37 | 6.5109e-03 | 3.5077e-02 |
| 4006.snp | 29739 | 941628 | T | A | 89 | 90 | 94 | 83 | 2.4280e-06 | 2.2174e-05 |
| 4035.snp | 29739 | 1223397 | G | A | 1 | 12 | 1 | 12 | 3.3529e-06 | 3.0393e-05 |
| 4036.snp | 29739 | 1223550 | A | G | 1 | 22 | 1 | 29 | 8.8816e-12 | 1.0194e-10 |
| 4095.snp | 30170 | 1306976 | A | G | 37 | 35 | 25 | 29 | 3.9339e-04 | 2.8063e-03 |
| 422.snp | 29868 | 127630 | G | C | 39 | 36 | 62 | 61 | 4.9466e-04 | 3.4481e-03 |
| 4253.snp | 29805 | 72183 | G | A | 27 | 26 | 51 | 40 | 4.0983e-03 | 2.3550e-02 |
| 4305.snp | 27519 | 165931 | C | T | 35 | 29 | 15 | 18 | 6.2317e-03 | 3.3721e-02 |
| 4364.snp | 28320 | 241421 | G | A | 87 | 70 | 104 | 76 | 1.1688e-03 | 7.5951e-03 |
| 4677.snp | 29794 | 259656 | C | A | 118 | 80 | 110 | 97 | 5.7079e-03 | 3.1445e-02 |
| 4687.snp | 29794 | 446690 | T | C | 65 | 52 | 141 | 99 | 8.6766e-04 | 5.7837e-03 |
| 4694.snp | 29794 | 534269 | G | A | 95 | 65 | 86 | 68 | 8.0917e-03 | 4.2704e-02 |
| 4863.snp | 29908 | 162503 | G | C | 93 | 68 | 221 | 147 | 1.5801e-03 | 9.9431e-03 |
| 4997.snp | 29908 | 1469310 | T | C | 52 | 76 | 120 | 81 | 6.0300e-03 | 3.2964e-02 |
| 4998.snp | 29908 | 1469689 | A | C | 30 | 102 | 111 | 86 | 8.1780e-05 | 6.4183e-04 |
| 5101.snp | 29724 | 120146 | T | C | 56 | 51 | 65 | 57 | 9.3757e-05 | 7.2781e-04 |
| 5159.snp | 29844 | 430388 | T | C | 117 | 107 | 52 | 51 | 1.8791e-05 | 1.5840e-04 |
| 5181.snp | 29844 | 641032 | C | A | 3592 | 2098 | 9024 | 5289 | 1.6638e-12 | 1.9651e-11 |
| 5210.snp | 29844 | 789273 | G | A | 61 | 46 | 65 | 48 | 5.5932e-03 | 3.0973e-02 |
| 5248.snp | 29844 | 1089448 | T | A | 33 | 30 | 142 | 103 | 1.5989e-03 | 1.0024e-02 |
| 5287.snp | 29912 | 115303 | C | T | 199 | 162 | 132 | 102 | 2.9826e-05 | 2.4631e-04 |
| 5474.snp | 30147 | 38510 | C | T | 360 | 226 | 229 | 144 | 3.8562e-03 | 2.2819e-02 |
| 5484.snp | 30147 | 75302 | T | C | 1502 | 844 | 1669 | 947 | 3.9553e-04 | 2.8192e-03 |
| 5502.snp | 30147 | 391547 | G | A | 15 | 17 | 25 | 28 | 1.8736e-03 | 1.1543e-02 |
| 5527.snp | 30147 | 634833 | T | C | 102 | 170 | 193 | 140 | 1.7346e-05 | 1.4694e-04 |
| 5613.snp | 30147 | 1323733 | A | G | 72 | 56 | 15 | 180 | 1.4073e-03 | 8.9813e-03 |
| 5630.snp | 30147 | 1401537 | T | C | 1061 | 821 | 390 | 429 | 3.7873e-30 | 5.2077e-29 |
| 5677.snp | 30147 | 1782894 | T | A | 81 | 66 | 78 | 69 | 1.3451e-04 | 1.0311e-03 |
| 5751.snp | 30147 | 2177694 | C | T | 334 | 211 | 290 | 199 | 5.6103e-04 | 3.8634e-03 |
| 5759.snp | 30147 | 2285239 | C | T | 0 | 16 | 1 | 14 | 3.9062e-03 | 2.2799e-02 |
| 5791.snp | 30147 | 2466355 | C | T | 51 | 41 | 21 | 23 | 2.4800e-03 | 1.5073e-02 |
| 5794.snp | 30147 | 2481782 | C | A | 11 | 12 | 16 | 17 | 9.2645e-03 | 4.7771e-02 |
| 5938.snp | 30147 | 3330752 | G | C | 26 | 24 | 19 | 21 | 5.3219e-03 | 2.9547e-02 |
| 6008.snp | 30147 | 3983097 | A | C | 57 | 50 | 162 | 137 | 1.6415e-04 | 1.2317e-03 |
| 6032.snp | 30147 | 4607094 | A | G | 160 | 150 | 102 | 89 | 1.6649e-06 | 1.5571e-05 |
| 6111.snp | 28460 | 22883 | G | T | 573 | 390 | 2022 | 1399 | 3.8632e-09 | 4.1184e-08 |
| 6168.snp | 28152 | 226018 | C | T | 62 | 48 | 26 | 24 | 5.3219e-03 | 2.9528e-02 |
| 6316.snp | 28830 | 55205 | C | A | 1 | 48 | 0 | 44 | 4.3703e-05 | 3.5239e-04 |
| 6678.snp | 29863 | 138101 | T | A | 126 | 88 | 60 | 52 | 1.7833e-03 | 1.1067e-02 |
| 6910.snp | 28629 | 123664 | T | C | 36 | 850 | 41 | 516 | 3.3376e-62 | 4.8141e-61 |
| 6972.snp | 29200 | 23655 | T | C | 38070 | 24411 | 29174 | 27942 | 2.1921e-293 | 3.2725e-292 |
| 6999.snp | 30190 | 282511 | C | T | 447 | 432 | 205 | 201 | 1.0250e-16 | 1.2909e-15 |
| 7494.snp | 30076 | 1075897 | G | A | 29 | 25 | 19 | 25 | 6.5109e-03 | 3.5010e-02 |
| 759.snp | 30064 | 198012 | T | C | 494 | 353 | 848 | 480 | 3.9205e-03 | 2.2727e-02 |
| 7646.snp | 30005 | 54698 | C | T | 5 | 60 | 4 | 45 | 2.4043e-05 | 1.9971e-04 |
| 7714.snp | 29981 | 151462 | C | T | 54 | 47 | 103 | 88 | 4.1201e-04 | 2.9245e-03 |
| 7767.snp | 27742 | 36799 | A | C | 263 | 169 | 725 | 476 | 8.0293e-04 | 5.3943e-03 |
| 7796.snp | 28582 | 161121 | T | C | 61 | 191 | 623 | 387 | 1.6110e-05 | 1.3688e-04 |
| 7797.snp | 28582 | 161198 | G | C | 102 | 332 | 980 | 575 | 8.4618e-05 | 6.6350e-04 |
| 7799.snp | 28582 | 161428 | C | T | 17 | 52 | 151 | 98 | 6.5492e-03 | 3.5194e-02 |
| 7816.snp | 30183 | 669532 | T | C | 210 | 182 | 230 | 175 | 1.7414e-07 | 1.7311e-06 |
| 7968.snp | 27574 | 121594 | A | G | 107 | 80 | 57 | 52 | 3.7392e-04 | 2.6764e-03 |
| 8015.snp | 30169 | 817783 | A | G | 5594 | 4288 | 8277 | 6071 | 1.0725e-140 | 1.5817e-139 |
| 810.snp | 29729 | 199927 | C | T | 432 | 360 | 291 | 238 | 3.4436e-12 | 4.0007e-11 |
| 8314.snp | 29848 | 72042 | G | A | 24 | 28 | 72 | 53 | 5.2635e-03 | 2.9299e-02 |
| 8343.snp | 29848 | 316916 | G | C | 42 | 34 | 21 | 27 | 6.6304e-03 | 3.5541e-02 |
| 8407.snp | 29848 | 962812 | G | C | 81 | 82 | 191 | 145 | 1.5176e-06 | 1.4256e-05 |
| 8533.snp | 28644 | 204405 | C | T | 2871 | 2363 | 4893 | 3171 | 1.1947e-43 | 1.6835e-42 |
| 855.snp | 30069 | 303373 | G | C | 372 | 322 | 608 | 561 | 1.7652e-18 | 2.2700e-17 |
| 939.snp | 29168 | 40836 | C | T | 113 | 104 | 90 | 71 | 2.5170e-04 | 1.8370e-03 |
|  |  |  |  |  |  |  |  |  |  |  |
