# Supplementary material for: Genomic imprinting, methylation and parent-of-origin effects in reciprocal hybrid endosperm of castor bean
Source: Nucleic Acids Res. 2014 May 5;42(11):6987–98. doi: 10.1093/nar/gku375 (PMC4066788; doi:10.1093/nar/gku375)
Supplement: SUPPLEMENTARY DATA [file supp_gku375_nar-00469-v-2014-File009.zip › Supplementary_Table_S5.docx]

| **Supplementary Table S5.** Genes with maternally-biased expression (MEGs, >90% maternal reads in both hybrid endosperms). m, Maternal alleles; p, Paternal alleles. The marks * denote that these genes were experimentally confirmed. | | | | | | | | | | | |
| --- | --- | --- | --- | --- | --- | --- | --- | --- | --- | --- | --- |
| **SNP No.** | **Scaffold** | **Position** | **SNP_**  **ZB107** | **SNP_**  **ZB306** | **ZB107×ZB306** | | **ZB306×ZB107** | |  |  | **Annotation** |
|  |  |  |  |  | **m_ZB107** | **p_ZB306** | **m_ZB306** | **p_ZB107** | **FDR** | **Gene ID** |  |
| 356.snp | 27448 | 22694 | G | T | 16 | 1 | 33 | 7 | 2.3538e-02 | 27448.m000067 | conserved hypothetical protein |
| 358.snp* | 27448 | 28491 | G | A | 132 | 6 | 88 | 1 | 2.2281e-05 | 27448.m000068 | amino acid transporter, putative |
| 1579.snp | 27467 | 5670 | A | T | 265 | 7 | 127 | 8 | 1.3277e-12 | 27467.m000162 | Allene oxide cyclase 4, chloroplast precursor, putative |
| 3204.snp* | 27837 | 97014 | A | G | 852 | 18 | 1185 | 30 | 2.1083e-18 | 27837.m000165 | endomembrane-associated protein, putative |
| 3374.snp | 27914 | 110136 | A | C | 17 | 0 | 19 | 2 | 9.4747e-03 | 27914.m000418 | spliceosome associated protein, putative |
| 3375.snp | 27914 | 133718 | G | A | 71 | 3 | 96 | 14 | 1.3774e-09 | 27914.m000421 | nnp-1 protein, putative |
| 7674.snp | 27942 | 37838 | C | T | 201 | 6 | 84 | 2 | 1.1792e-03 | 27942.m000156 | zinc/iron transporter, putative |
| 738.snp | 27955 | 130423 | T | A | 13 | 0 | 12 | 0 | 2.2675e-02 | 27955.m000390 | conserved hypothetical protein |
| 3543.snp | 27956 | 120958 | A | T | 16 | 0 | 101 | 7 | 2.4436e-02 | 27956.m000354 | conserved hypothetical protein |
| 3023.snp* | 28124 | 8721 | G | A | 111 | 1 | 192 | 3 | 5.8432e-13 | 28124.m000232 | Cyclic nucleotide-gated ion channel, putative |
| 640.snp | 28202 | 12146 | A | C | 57 | 2 | 58 | 11 | 1.5776e-04 | 28202.m000129 | smad nuclear interacting protein, putative |
| 419.snp | 28350 | 30030 | A | G | 34 | 0 | 38 | 7 | 2.6445e-03 | 28350.m000106 | RNA-binding protein, putative |
| 3058.snp | 28507 | 28349 | C | T | 36 | 1 | 37 | 6 | 1.5899e-03 | 28507.m000157 | adenosine kinase, putative |
| 3066.snp | 28507 | 130906 | G | A | 22 | 0 | 34 | 8 | 3.1561e-02 | 28507.m000165 | conserved hypothetical protein |
| 3943.snp | 28524 | 7075 | C | T | 52 | 4 | 54 | 3 | 5.1252e-03 | 28524.m000012 | dehydroascorbate reductase, putative |
| 1844.snp | 28583 | 35439 | C | A | 25 | 0 | 107 | 1 | 4.7945e-02 | 28583.m000109 | conserved hypothetical protein |
| 1655.snp | 28592 | 97287 | A | C | 28 | 0 | 52 | 6 | 1.0500e-06 | 28592.m000289 | conserved hypothetical protein |
| 7438.snp | 28611 | 37884 | A | T | 184 | 4 | 105 | 2 | 6.6480e-07 | 28611.m000102 | RNA-binding protein, putative |
| 6054.snp* | 28623 | 116965 | C | G | 1695 | 97 | 678 | 27 | 6.5132e-11 | 28623.m000402 | histone h1/h5, putative |
| 6074.snp | 28693 | 7885 | C | T | 41 | 4 | 18 | 1 | 1.0480e-03 | 28693.m000103 | Pre-rRNA-processing protein ESF1, putative |
| 7973.snp* | 28738 | 106954 | G | T | 162 | 3 | 131 | 21 | 2.3387e-11 | 28738.m000149 | glutathione-s-transferase theta, gst, putative |
| 3425.snp* | 28748 | 89177 | G | A | 101 | 0 | 35 | 0 | 1.5872e-04 | 28748.m000215 | ubiquitin-protein ligase, putative |
| 6478.snp | 28833 | 46875 | T | C | 20 | 0 | 12 | 0 | 2.2737e-02 | 28833.m000159 | 1-phosphatidylinositol-4,5-bisphosphate phosphodiesterase, putative |
| 6738.snp* | 29279 | 26382 | C | T | 143 | 0 | 112 | 0 | 4.3196e-04 | 29279.m000134 | glutaredoxin-1, grx1, putative |
| 1903.snp | 29585 | 14910 | C | T | 510 | 9 | 355 | 5 | 8.7267e-04 | 29585.m000574 | heat shock protein, putative |
| 1904.snp | 29585 | 91659 | T | C | 39 | 1 | 66 | 14 | 2.5071e-04 | 29585.m000583 | amino acid binding protein, putative |
| 3098.snp* | 29595 | 4705 | T | A | 1908 | 11 | 749 | 2 | 1.1422e-13 | 29595.m000277 | Tyramine N-feruloyltransferase 4/11, putative |
| 1658.snp | 29599 | 13596 | T | G | 55 | 2 | 84 | 22 | 1.1802e-03 | 29599.m000170 | conserved hypothetical protein |
| 7763.snp | 29609 | 272448 | A | G | 47 | 0 | 43 | 7 | 5.2068e-04 | 29609.m000609 | Dynamin-2A, putative |
| 433.snp | 29610 | 116390 | G | A | 30 | 0 | 38 | 9 | 2.2360e-02 | 29610.m000401 | conserved hypothetical protein |
| 6334.snp | 29630 | 107215 | G | A | 26 | 0 | 58 | 1 | 1.5792e-04 | 29630.m000793 | glycogen phosphorylase, putative |
| 6344.snp | 29630 | 368039 | C | T | 10 | 0 | 11 | 0 | 2.2768e-02 | 29630.m000833 | alpha-amylase, putative |
| 7680.snp* | 29633 | 2606 | A | C | 1764 | 87 | 2270 | 552 | 8.6431e-89 | 29633.m000882 | glycine-rich RNA-binding protein, putative |
| 7748.snp | 29633 | 8807 | T | C | 203 | 14 | 147 | 9 | 1.5283e-05 | 29633.m000883 | conserved hypothetical protein |
| 7686.snp* | 29633 | 219166 | C | A | 127 | 4 | 79 | 3 | 7.2787e-07 | 29633.m000919 | conserved hypothetical protein |
| 4587.snp | 29634 | 111270 | A | T | 24 | 0 | 12 | 0 | 1.7972e-03 | 29634.m002068 | conserved hypothetical protein |
| 4600.snp | 29634 | 229139 | A | T | 80 | 9 | 200 | 34 | 3.5647e-10 | 29634.m002088 | ring finger protein, putative |
| 4604.snp | 29634 | 333285 | C | T | 50 | 2 | 137 | 29 | 4.5875e-08 | 29634.m002104 | conserved hypothetical protein |
| 4620.snp | 29634 | 416274 | A | C | 12 | 1 | 15 | 1 | 2.7756e-02 | 29634.m002119 | syntaxin, arabidopsis thaliana, putative |
| 4451.snp | 29648 | 72594 | A | T | 10 | 0 | 14 | 0 | 9.6446e-03 | 29648.m001921 | Basic blue protein, putative |
| 1596.snp* | 29669 | 151081 | C | T | 1144 | 28 | 790 | 16 | 1.4383e-42 | 29669.m000815 | 60S acidic ribosomal protein P0, putative |
| 1597.snp | 29669 | 181752 | A | C | 27 | 5 | 33 | 7 | 2.3570e-02 | 29669.m000820 | syntaxin, putative |
| 6761.snp | 29670 | 53404 | G | A | 1464 | 12 | 1818 | 47 | 1.2480e-63 | 29670.m000149 | lupus la ribonucleoprotein, putative |
| 3800.snp | 29682 | 202533 | C | T | 58 | 1 | 42 | 9 | 5.7900e-03 | 29682.m000596 | conserved hypothetical protein |
| 3799.snp | 29682 | 201439 | C | A | 18 | 1 | 31 | 5 | 4.8157e-03 | 29682.m000596 | conserved hypothetical protein |
| 3802.snp* | 29682 | 214949 | T | C | 107 | 6 | 95 | 2 | 1.4733e-03 | 29682.m000597 | big map kinase/bmk, putative |
| 2006.snp | 29692 | 148436 | G | A | 43 | 1 | 34 | 7 | 1.1551e-02 | 29692.m000529 | phosphoglucomutase, putative |
| 2010.snp* | 29692 | 283134 | C | A | 3105 | 47 | 953 | 24 | 5.1948e-35 | 29692.m000544 | ARF GTPase activator, putative |
| 1418.snp* | 29693 | 304535 | T | C | 89 | 10 | 105 | 12 | 4.8839e-11 | 29693.m001994 | Protein AFR, putative |
| 1430.snp | 29693 | 522211 | G | T | 44 | 5 | 51 | 9 | 2.9413e-04 | 29693.m002028 | conserved hypothetical protein |
| 1431.snp* | 29693 | 524314 | A | G | 198 | 1 | 305 | 8 | 4.5947e-11 | 29693.m002029 | conserved hypothetical protein |
| 1436.snp* | 29693 | 541825 | G | C | 166 | 2 | 199 | 2 | 3.4272e-05 | 29693.m002034 | 50 kDa &#946;-ketoavyl-ACP synthase (KASB) |
| 3927.snp | 29709 | 156562 | T | C | 188 | 5 | 54 | 2 | 6.9949e-04 | 29709.m001197 | 40S ribosomal protein S11, putative |
| 7517.snp | 29736 | 97153 | C | G | 130 | 8 | 26 | 5 | 2.3362e-02 | 29736.m002015 | UDP-galactose transporter, putative |
| 4765.snp | 29737 | 331885 | C | T | 32 | 2 | 61 | 8 | 5.1338e-06 | 29737.m001250 | Ribonuclease III, putative |
| 8283.snp | 29740 | 81607 | C | T | 15 | 1 | 35 | 8 | 3.1480e-02 | 29740.m000478 | mitochondrial carrier protein, putative |
| 86.snp | 29745 | 106238 | C | A | 26 | 4 | 20 | 3 | 1.9642e-02 | 29745.m000366 | RNA binding motif protein, putative |
| 6933.snp | 29751 | 65835 | G | C | 77 | 2 | 136 | 4 | 3.9822e-04 | 29751.m001799 | RNA binding motif protein, putative |
| 6932.snp* | 29751 | 63734 | A | T | 229 | 10 | 195 | 8 | 2.6417e-05 | 29751.m001799 | RNA binding motif protein, putative |
| 6956.snp | 29751 | 452743 | G | A | 77 | 2 | 260 | 64 | 3.9008e-11 | 29751.m001870 | protein phosphatase 2c, putative |
| 6965.snp | 29751 | 572952 | A | G | 86 | 6 | 50 | 1 | 2.8650e-03 | 29751.m001884 | ubiquinone biosynthesis protein, putative |
| 6967.snp | 29751 | 639030 | A | G | 78 | 5 | 102 | 16 | 1.5824e-04 | 29751.m001894 | sorting and assembly machinery (sam50) protein, putative |
| 4542.snp* | 29758 | 101355 | C | T | 47 | 1 | 22 | 3 | 7.9771e-03 | 29758.m000646 | run and tbc1 domain containing 3, plant, putative |
| 3279.snp | 29765 | 77114 | A | T | 709 | 22 | 706 | 25 | 1.0631e-13 | 29765.m000727 | nucleic acid binding protein, putative |
| 3283.snp* | 29765 | 84086 | G | A | 222 | 10 | 293 | 7 | 1.0154e-12 | 29765.m000727 | nucleic acid binding protein, putative |
| 3287.snp | 29765 | 106691 | C | A | 36 | 1 | 39 | 5 | 1.4111e-04 | 29765.m000729 | phosphoribosylamine--glycine ligase |
| 3234.snp | 29780 | 228276 | G | A | 20 | 3 | 23 | 4 | 3.2379e-02 | 29780.m001342 | conserved hypothetical protein |
| 3237.snp* | 29780 | 348335 | C | T | 702 | 37 | 1040 | 83 | 1.3149e-25 | 29780.m001362 | nucleic acid binding protein, putative |
| 3244.snp* | 29780 | 443217 | A | G | 67 | 7 | 72 | 6 | 7.0190e-09 | 29780.m001373 | eukaryotic translation initiation factor 3 subunit, putative |
| 3243.snp | 29780 | 443091 | T | A | 180 | 14 | 213 | 40 | 3.3560e-12 | 29780.m001373 | eukaryotic translation initiation factor 3 subunit, putative |
| 3245.snp | 29780 | 448732 | T | C | 52 | 1 | 45 | 8 | 8.7885e-04 | 29780.m001374 | peptide transporter, putative |
| 3300.snp | 29784 | 30219 | A | T | 78 | 4 | 33 | 5 | 2.0741e-03 | 29784.m000358 | RING-H2 finger protein ATL4M, putative |
| 3305.snp | 29784 | 79822 | G | C | 19 | 1 | 20 | 3 | 1.9738e-02 | 29784.m000365 | conserved hypothetical protein |
| 3311.snp | 29784 | 89008 | A | G | 24 | 2 | 66 | 9 | 4.3568e-04 | 29784.m000366 | DNA-directed RNA polymerase II largest subunit, putative |
| 3313.snp | 29784 | 144304 | T | A | 112 | 3 | 77 | 5 | 2.0579e-05 | 29784.m000370 | Arginine/serine-rich-splicing factor, putative |
| 3312.snp | 29784 | 142442 | A | G | 47 | 4 | 58 | 6 | 3.3369e-07 | 29784.m000370 | Arginine/serine-rich-splicing factor, putative |
| 199.snp | 29792 | 198561 | C | T | 10 | 0 | 77 | 1 | 7.3293e-20 | 29792.m000624 | cytochrome P450, putative |
| 6576.snp | 29806 | 62906 | A | C | 55 | 3 | 80 | 16 | 1.4966e-05 | 29806.m000937 | Splicing factor 3B subunit, putative |
| 4382.snp | 29814 | 39690 | T | C | 43 | 2 | 54 | 12 | 2.0050e-03 | 29814.m000721 | conserved hypothetical protein |
| 7233.snp | 29816 | 119480 | A | C | 71 | 4 | 87 | 17 | 5.1461e-06 | 29816.m000674 | conserved hypothetical protein |
| 7236.snp* | 29816 | 188430 | C | T | 40 | 0 | 31 | 2 | 1.6054e-05 | 29816.m000679 | conserved hypothetical protein |
| 7237.snp | 29816 | 198420 | G | A | 187 | 6 | 78 | 1 | 3.0678e-05 | 29816.m000680 | Vitellogenic carboxypeptidase, putative |
| 7238.snp | 29816 | 224144 | A | G | 23 | 3 | 23 | 4 | 3.2296e-02 | 29816.m000684 | (Di)nucleoside polyphosphate hydrolase, putative |
| 7684.snp | 29822 | 9522 | A | G | 49 | 2 | 70 | 14 | 5.9548e-05 | 29822.m003326 | l-aspartate oxidase, putative |
| 7603.snp* | 29827 | 497630 | A | T | 16 | 1 | 20 | 3 | 1.9656e-02 | 29827.m002618 | pentatricopeptide repeat-containing protein, putative |
| 7610.snp | 29827 | 599357 | C | G | 26 | 5 | 18 | 2 | 2.3346e-02 | 29827.m002636 | Beta-amylase, putative |
| 7612.snp | 29827 | 611636 | A | C | 35 | 1 | 31 | 6 | 1.6643e-02 | 29827.m002639 | Rop guanine nucleotide exchange factor, putative |
| 7624.snp | 29827 | 666785 | A | C | 19 | 0 | 22 | 3 | 7.9651e-03 | 29827.m002648 | aberrant large forked product, putative |
| 7628.snp* | 29827 | 701020 | G | T | 254 | 42 | 139 | 16 | 1.4433e-05 | 29827.m002653 | AP-1 complex subunit gamma-2, putative |
| 7629.snp | 29827 | 736408 | T | G | 38 | 1 | 46 | 7 | 9.5951e-05 | 29827.m002656 | conserved hypothetical protein |
| 7631.snp | 29827 | 793885 | C | T | 113 | 5 | 71 | 10 | 2.5355e-07 | 29827.m002663 | conserved hypothetical protein |
| 5435.snp | 29830 | 45731 | C | T | 71 | 5 | 79 | 15 | 9.7574e-06 | 29830.m001387 | calcium-dependent protein kinase, putative |
| 5446.snp | 29830 | 374905 | C | T | 11 | 0 | 11 | 0 | 2.2815e-02 | 29830.m001434 | poly(A) polymerase, putative |
| 5447.snp* | 29830 | 387710 | C | A | 101 | 10 | 130 | 15 | 6.1870e-05 | 29830.m001435 | plant ubiquilin, putative |
| 372.snp | 29839 | 123752 | T | G | 76 | 1 | 49 | 10 | 2.0222e-03 | 29839.m000431 | diphosphomevalonate decarboxylase, putative |
| 6640.snp | 29840 | 416315 | G | A | 80 | 5 | 30 | 6 | 1.6655e-02 | 29840.m000624 | conserved hypothetical protein |
| 1066.snp | 29851 | 809006 | G | A | 53 | 0 | 128 | 14 | 2.7841e-15 | 29851.m002492 | Flavonol 4'-sulfotransferase, putative |
| 1067.snp | 29851 | 812549 | G | A | 35 | 3 | 222 | 37 | 2.0528e-05 | 29851.m002493 | sulfotransferase, putative |
| 5363.snp | 29898 | 30504 | C | T | 74 | 11 | 134 | 29 | 2.0938e-07 | 29898.m000065 | Pectate lyase precursor, putative |
| 7473.snp | 29900 | 252651 | A | G | 30 | 3 | 32 | 6 | 7.8025e-03 | 29900.m001577 | Magnesium-chelatase subunit chlI, chloroplast precursor, putative |
| 7478.snp* | 29900 | 429067 | T | C | 257 | 11 | 143 | 5 | 1.9620e-06 | 29900.m001607 | Heterogeneous nuclear ribonucleoprotein A1, putative |
| 1236.snp* | 29905 | 179083 | C | T | 160 | 16 | 98 | 16 | 1.2034e-08 | 29905.m000439 | Anthocyanin 5-aromatic acyltransferase, putative |
| 6502.snp* | 29916 | 165488 | G | T | 1321 | 16 | 321 | 6 | 8.2720e-20 | 29916.m000533 | heat-shock protein, putative |
| 2011.snp | 29917 | 301451 | C | A | 70 | 5 | 24 | 2 | 4.3609e-04 | 29917.m001982 | conserved hypothetical protein |
| 2014.snp | 29917 | 484400 | C | A | 68 | 4 | 31 | 7 | 4.5196e-02 | 29917.m002001 | conserved hypothetical protein |
| 2024.snp* | 29917 | 586140 | C | A | 52 | 5 | 69 | 13 | 3.8986e-05 | 29917.m002014 | Cerebral protein, putative |
| 2026.snp | 29917 | 597353 | A | T | 29 | 0 | 29 | 4 | 2.6550e-03 | 29917.m002015 | protein kinase atn1, putative |
| 7348.snp | 29950 | 52262 | A | G | 10 | 0 | 164 | 0 | 6.3899e-03 | 29950.m001124 | conserved hypothetical protein |
| 3427.snp | 29966 | 56452 | G | A | 23 | 0 | 33 | 7 | 2.3554e-02 | 29966.m000222 | serine/threonine protein kinase, putative |
| 2276.snp | 29969 | 108161 | G | A | 45 | 4 | 17 | 2 | 2.4469e-02 | 29969.m000270 | ribosomal RNA methyltransferase, putative |
| 1016.snp | 29977 | 84701 | A | G | 20 | 1 | 34 | 4 | 3.2862e-04 | 29977.m000261 | Mitochondrial GTPase, putative |
| 6095.snp* | 29993 | 126233 | C | T | 143 | 1 | 174 | 3 | 1.3914e-11 | 29993.m001038 | glyoxalase II, putative |
| 2234.snp* | 30026 | 273291 | A | T | 1204 | 37 | 368 | 73 | 6.3648e-23 | 30026.m001474 | preprotein translocase secy subunit, putative |
| 2242.snp | 30026 | 346711 | C | T | 54 | 7 | 39 | 4 | 2.1815e-05 | 30026.m001484 | Heat shock factor protein, putative |
| 2251.snp* | 30026 | 622314 | A | G | 20 | 0 | 18 | 3 | 4.4697e-02 | 30026.m001516 | mitochondrial inner membrane protease subunit, putative |
| 8183.snp* | 30059 | 93493 | T | C | 147 | 9 | 123 | 2 | 6.3289e-10 | 30059.m000466 | ELL-associated factor, putative |
| 201.snp | 30068 | 41581 | G | A | 121 | 7 | 82 | 12 | 2.9148e-08 | 30068.m002516 | hypothetical protein |
| 202.snp | 30068 | 75824 | A | G | 66 | 4 | 116 | 17 | 2.2983e-11 | 30068.m002522 | 26S proteasome non-atpase regulatory subunit, putative |
| 206.snp | 30068 | 337763 | G | A | 27 | 3 | 59 | 8 | 1.2163e-03 | 30068.m002570 | conserved hypothetical protein |
| 1047.snp* | 30072 | 244261 | C | T | 722 | 87 | 1323 | 114 | 1.8951e-20 | 30072.m000961 | conserved hypothetical protein |
| 683.snp | 30075 | 460147 | G | A | 62 | 2 | 57 | 11 | 1.0488e-02 | 30075.m001187 | conserved hypothetical protein |
| 3792.snp | 30128 | 41815 | T | C | 148 | 2 | 140 | 5 | 9.4505e-11 | 30128.m008600 | aldo/keto reductase, putative |
| 490.snp* | 30128 | 45520 | G | A | 118 | 12 | 104 | 27 | 1.9743e-04 | 30128.m008601 | aldo/keto reductase, putative |
| 513.snp* | 30128 | 441911 | T | C | 125 | 0 | 109 | 20 | 4.8124e-07 | 30128.m008669 | vesicle-associated membrane protein, putative |
| 522.snp | 30128 | 514696 | G | T | 41 | 5 | 20 | 2 | 3.4995e-03 | 30128.m008688 | conserved hypothetical protein |
| 530.snp | 30128 | 790197 | C | T | 25 | 0 | 22 | 0 | 3.2338e-02 | 30128.m008739 | excision repair cross-complementing 1 ercc1, putative |
| 531.snp* | 30128 | 797269 | G | A | 100 | 7 | 64 | 10 | 3.5769e-06 | 30128.m008741 | UDP-N-acetylglucosamine transferase subunit alg13, putative |
| 532.snp* | 30128 | 814607 | T | C | 114 | 5 | 113 | 7 | 1.8309e-05 | 30128.m008744 | chloroplast-targeted copper chaperone, putative |
| 548.snp | 30128 | 1614824 | T | C | 39 | 2 | 21 | 3 | 1.9683e-02 | 30128.m008904 | conserved hypothetical protein |
| 551.snp | 30128 | 1744911 | G | A | 57 | 5 | 25 | 4 | 1.4638e-02 | 30128.m008925 | conserved hypothetical protein |
| 587.snp | 30128 | 2139523 | C | T | 36 | 4 | 55 | 10 | 2.1522e-04 | 30128.m009008 | hypothetical protein |
| 595.snp | 30128 | 2195151 | A | T | 27 | 2 | 42 | 7 | 5.2116e-04 | 30128.m009021 | conserved hypothetical protein |
| 1077.snp | 30138 | 127551 | G | A | 82 | 8 | 332 | 15 | 1.1665e-09 | 30138.m003840 | conserved hypothetical protein |
| 4311.snp | 30143 | 162842 | T | C | 11 | 0 | 78 | 10 | 6.4343e-03 | 30143.m001180 | zinc finger protein, putative |
| 6023.snp* | 30147 | 4394375 | G | A | 1051 | 60 | 1934 | 125 | 4.5637e-186 | 30147.m014083 | conserved hypothetical protein |
| 6527.snp | 30152 | 1612269 | A | G | 68 | 8 | 71 | 19 | 6.0062e-03 | 30152.m002434 | conserved hypothetical protein |
| 4146.snp* | 30170 | 2418995 | C | G | 1125 | 20 | 939 | 37 | 8.3330e-06 | 30170.m013704 | conserved hypothetical protein |
| 4126.snp | 30170 | 1851577 | G | A | 32 | 3 | 31 | 6 | 1.6667e-02 | 30170.m013935 | Protein Z, putative |
| 4136.snp | 30170 | 2140036 | A | G | 28 | 2 | 20 | 3 | 1.9725e-02 | 30170.m013983 | zinc ion binding protein, putative |
| 4157.snp* | 30170 | 2806400 | C | T | 39 | 1 | 16 | 1 | 3.2412e-03 | 30170.m014093 | 26S protease regulatory subunit S10b, putative |
| 4163.snp* | 30170 | 3142383 | T | C | 159 | 9 | 77 | 9 | 1.0780e-03 | 30170.m014148 | amino acid transporter, putative |
| 4169.snp | 30170 | 3260920 | C | T | 49 | 1 | 89 | 17 | 2.4280e-06 | 30170.m014165 | Myosin heavy chain, striated muscle, putative |
| 4149.snp* | 30170 | 2431623 | T | C | 165 | 8 | 99 | 10 | 1.5634e-05 | 30170.m014221 | transporter, putative |
| 4227.snp | 30170 | 4533650 | A | G | 46 | 0 | 83 | 0 | 6.2582e-05 | 30170.m014245 | Squamosa promoter-binding protein, putative |
| 4182.snp | 30170 | 3685567 | G | C | 45 | 3 | 22 | 3 | 7.9831e-03 | 30170.m014274 | t4o12.20, putative |
| 4185.snp | 30170 | 3707608 | G | A | 84 | 0 | 59 | 0 | 1.5807e-04 | 30170.m014280 | ring finger protein, putative |
| 4187.snp | 30170 | 3874616 | T | C | 10 | 0 | 36 | 0 | 2.2925e-02 | 30170.m014311 | prolyl endopeptidase, putative |
| 4189.snp | 30170 | 3888230 | A | G | 21 | 0 | 40 | 4 | 9.0886e-06 | 30170.m014312 | estradiol 17 beta-dehydrogenase, putative |
| 4190.snp* | 30170 | 3972456 | A | G | 1182 | 75 | 978 | 29 | 2.7162e-23 | 30170.m014328 | conserved hypothetical protein |
| 4195.snp* | 30170 | 4029629 | G | C | 8906 | 579 | 7205 | 240 | 5.1147e-106 | 30170.m014341 | conserved hypothetical protein |
| 2562.snp* | 30174 | 1749515 | C | T | 2798 | 49 | 2127 | 78 | 2.1475e-20 | 30174.m008614 | Aquaporin PIP1.3, putative |
| 2607.snp | 30174 | 2086835 | G | A | 157 | 20 | 303 | 33 | 2.7472e-13 | 30174.m008670 | Late embryogenesis abundant protein Lea14-A, putative |
| 2611.snp | 30174 | 2109678 | G | A | 114 | 8 | 99 | 2 | 9.8382e-05 | 30174.m008673 | conserved hypothetical protein |
| 2631.snp* | 30174 | 2178228 | C | T | 330 | 25 | 33 | 0 | 4.1574e-04 | 30174.m008685 | hypothetical protein |
| 2637.snp* | 30174 | 2201450 | T | A | 10 | 0 | 16 | 0 | 6.4592e-03 | 30174.m008690 | Protein UNUSUAL FLORAL ORGANS, putative |
| 2652.snp | 30174 | 2278563 | T | A | 20 | 0 | 13 | 0 | 2.7829e-02 | 30174.m008702 | f27j15.15, putative |
| 2399.snp | 30174 | 179219 | T | C | 10 | 0 | 48 | 0 | 6.4692e-03 | 30174.m008767 | Palmitoyl-protein thioesterase 1 precursor, putative |
| 2409.snp | 30174 | 213769 | G | C | 35 | 3 | 14 | 1 | 9.6660e-03 | 30174.m008773 | hypothetical protein |
| 2526.snp | 30174 | 1365035 | C | T | 48 | 2 | 29 | 4 | 2.6527e-03 | 30174.m008983 | UDP-galactose:MGDG galactosyltransferase, putative |
| 2743.snp | 30174 | 3130787 | G | A | 57 | 0 | 47 | 0 | 1.0495e-02 | 30174.m009117 | pentatricopeptide repeat-containing protein, putative |
| 7911.snp | 30179 | 104268 | T | C | 23 | 0 | 68 | 0 | 4.9551e-03 | 30179.m000563 | serine/threonine protein kinase, putative |
| 7121.snp | 30190 | 3446225 | T | A | 24 | 0 | 11 | 0 | 2.2691e-02 | 30190.m010802 | Centromeric protein E, putative |
| 7125.snp | 30190 | 3556425 | G | T | 346 | 4 | 711 | 10 | 2.1639e-63 | 30190.m010822 | conserved hypothetical protein |
| 7128.snp* | 30190 | 3654558 | C | T | 113 | 3 | 104 | 20 | 2.0866e-07 | 30190.m010836 | protein binding protein, putative |
| 7001.snp | 30190 | 308340 | C | T | 51 | 3 | 36 | 0 | 1.6538e-02 | 30190.m010870 | smg-7, putative |
| 7009.snp | 30190 | 458170 | C | T | 91 | 8 | 34 | 5 | 8.7109e-04 | 30190.m010889 | coatomer gamma subunit, putative |
| 7014.snp | 30190 | 793033 | C | T | 206 | 3 | 63 | 12 | 1.1482e-04 | 30190.m010937 | sulfite reductase, putative |
| 7013.snp* | 30190 | 792244 | G | C | 131 | 0 | 98 | 0 | 9.8282e-05 | 30190.m010937 | sulfite reductase, putative |
| 7028.snp* | 30190 | 1161935 | G | A | 1081 | 129 | 1579 | 163 | 1.3161e-08 | 30190.m010992 | Vacuolar-processing enzyme precursor |
| 7029.snp* | 30190 | 1170708 | A | C | 281 | 20 | 186 | 29 | 1.4692e-16 | 30190.m010995 | heat shock protein 70 (HSP70)-interacting protein, putative |
| 7030.snp* | 30190 | 1211628 | G | T | 408 | 18 | 550 | 73 | 6.4219e-56 | 30190.m011003 | lipid binding protein, putative |
| 7033.snp | 30190 | 1266924 | G | A | 322 | 13 | 711 | 19 | 1.6746e-23 | 30190.m011011 | Amine oxidase [copper-containing] precursor, putative |
| 7048.snp* | 30190 | 2311363 | C | T | 114 | 10 | 90 | 14 | 1.0211e-03 | 30190.m011167 | hypothetical protein |
| 7061.snp | 30190 | 2520994 | A | C | 22 | 1 | 28 | 2 | 1.0066e-04 | 30190.m011209 | conserved hypothetical protein |
| 7074.snp* | 30190 | 2806483 | C | T | 220 | 19 | 208 | 10 | 3.5887e-05 | 30190.m011249 | rad25/xp-B DNA repair helicase, putative |
| 7093.snp | 30190 | 3033773 | T | C | 16 | 0 | 16 | 0 | 2.4274e-02 | 30190.m011279 | hypothetical protein |
| 7097.snp | 30190 | 3053266 | G | C | 30 | 3 | 50 | 1 | 1.6408e-04 | 30190.m011281 | sumo ligase, putative |
| 7105.snp | 30190 | 3143461 | G | C | 49 | 2 | 89 | 17 | 4.4475e-05 | 30190.m011294 | 50S ribosomal protein L25, putative |
| 6995.snp* | 30190 | 141645 | T | C | 179 | 6 | 107 | 19 | 2.7560e-08 | 30190.m011315 | conserved hypothetical protein |
| 7109.snp* | 30190 | 3200122 | A | G | 267 | 12 | 459 | 72 | 6.0603e-39 | 30190.m011345 | transcription factor, putative |
| 3430.snp | 30205 | 56246 | A | T | 48 | 0 | 36 | 0 | 1.7341e-08 | 30205.m001575 | Poly(rC)-binding protein, putative |
| 3436.snp* | 30205 | 467276 | T | G | 146 | 8 | 232 | 41 | 1.7372e-17 | 30205.m001590 | sugar transporter, putative |
| 7520.snp | 29736 | 190929 | G | C | 578 | 11 | 524 | 24 | 4.1700e-04 | 29736.m002024 | AMP-activated protein kinase, gamma regulatory subunit, putative |
| 7860.snp | 29453 | 26872 | A | G | 46 | 2 | 86 | 1 | 2.4347e-02 | 29453.m000062 | BRASSINOSTEROID INSENSITIVE 1-associated receptor kinase 1 precursor, putative |
