# Supplementary material for: Genomic imprinting, methylation and parent-of-origin effects in reciprocal hybrid endosperm of castor bean
Source: Nucleic Acids Res. 2014 May 5;42(11):6987–98. doi: 10.1093/nar/gku375 (PMC4066788; doi:10.1093/nar/gku375)
Supplement: SUPPLEMENTARY DATA [file supp_gku375_nar-00469-v-2014-File009.zip › Supplementary_Table_S6.docx]

| **Supplementary Table S6.** Genes with paternally-biased expression (PEGs, >90% paternal reads in both hybrid endosperms). m, Maternal alleles; p, Paternal alleles. | | | | | | | | | | | |
| --- | --- | --- | --- | --- | --- | --- | --- | --- | --- | --- | --- |
|  | | | | | | | | | | | |
| **SNP No.** | **Scaffold** | **Position** | **SNP_**  **ZB107** | **SNP_**  **ZB306** | **ZB107×ZB306** | | **ZB306×ZB107** | | **FDR** | **Gene ID** | **Annotation** |
|  |  |  |  |  | **m_ZB107** | **p_ZB306** | **m_ZB306** | **p_ZB107** |  |  |  |
| 2212.snp | 30074 | 555391 | G | A | 21 | 800 | 29 | 707 | 0 | 30074.m001408 | conserved hypothetical protein |
| 7646.snp | 30005 | 54698 | C | T | 5 | 60 | 4 | 45 | 1.9971e-04 | 30005.m001245 | conserved hypothetical protein |
| 2214.snp | 30074 | 557554 | T | C | 9 | 61 | 14 | 189 | 5.9594e-27 | 30074.m001409 | conserved hypothetical protein |
| 244.snp | 28166 | 531739 | C | T | 1 | 55 | 2 | 38 | 7.3181e-20 | 28166.m001094 | Homeobox protein FWA, putative |
| 245.snp | 28166 | 533958 | T | A | 7 | 95 | 1 | 66 | 2.5797e-39 | 28166.m001094 | Homeobox protein FWA, putative |
| 4036.snp | 29739 | 1223550 | A | G | 1 | 22 | 1 | 29 | 1.0194e-10 | 29739.m003761 | conserved hypothetical protein |
| 4035.snp | 29739 | 1223397 | G | A | 1 | 12 | 1 | 12 | 3.0393e-05 | 29739.m003761 | conserved hypothetical protein |
| 1133.snp | 30138 | 1075178 | G | A | 3 | 44 | 1 | 45 | 6.4793e-03 | 30138.m003979 | conserved hypothetical protein |
| 6316.snp | 28830 | 55205 | C | A | 1 | 48 | 0 | 44 | 3.5239e-04 | 28830.m000228 | cytochrome-c oxidase, putative |
| 5759.snp | 30147 | 2285239 | C | T | 0 | 16 | 1 | 14 | 2.2799e-02 | 30147.m014492 | conserved hypothetical protein |
| 6910.snp | 28629 | 123664 | T | C | 36 | 850 | 41 | 516 | 4.8141e-61 | 28629.m000565 | Beta-fructofuranosidase, insoluble  isoenzyme 1 precursor, putative |
|  |  |  |  |  |  |  |  |  |  |  |  |
