# Supplementary material for: Genomic imprinting, methylation and parent-of-origin effects in reciprocal hybrid endosperm of castor bean
Source: Nucleic Acids Res. 2014 May 5;42(11):6987–98. doi: 10.1093/nar/gku375 (PMC4066788; doi:10.1093/nar/gku375)
Supplement: SUPPLEMENTARY DATA [file supp_gku375_nar-00469-v-2014-File009.zip › Supplementary_Table_S7.docx]

| **Supplementary Table S7.** Maternal imprinted loci in intergenic region with >90% reads derivied from the maternal reads in both hybrid endosperms. | | | | | | | | | | | | | |  |
| --- | --- | --- | --- | --- | --- | --- | --- | --- | --- | --- | --- | --- | --- | --- |
|  | | | | | | | | | | | | | |  |
| **SNP No.** | **Scaffold** | **Position** | **SNP_**  **ZB107** | **SNP_**  **ZB306** | **ZB107×ZB306** | | **ZB306×ZB107** | |  | **Genomic region** | **Extented region** | **Gene ID** | |  |
|  |  |  |  |  | **m_ZB107** | **p_ZB306** | **m_ZB306** | **p_ZB107** | **FDR** |  |  |  |  |  |
| 3941.snp | 28524 | 3145 | C | T | 81 | 3 | 37 | 1 | 5.6121e-03 | 3'UTR | 3013-3343 | 28524.m000012 | |  |
| 6059.snp | 28623 | 122855 | G | C | 16 | 0 | 40 | 5 | 3.9806e-03 | 5'UTR | 122812-123978 | 28623.m000402 | |  |
| 4616.snp | 29634 | 409438 | A | C | 49 | 1 | 55 | 4 | 1.1664e-02 | 5'UTR | 409335-409595 | 29634.m002118 | |  |
| 458.snp | 29673 | 232083 | A | C | 17 | 0 | 20 | 0 | 1.9711e-02 | 3'UTR | 231679-232103 | 29673.m000930 | |  |
| 2005.snp | 29692 | 121838 | C | T | 69 | 3 | 51 | 1 | 2.9384e-04 | 3'UTR | 121639-121962 | 29692.m000527 | |  |
| 1423.snp | 29693 | 364798 | T | A | 320 | 8 | 95 | 2 | 1.1617e-10 | 5'UTR | 364516-364801 | 29693.m002009 | |  |
| 1429.snp | 29693 | 517068 | A | C | 16 | 1 | 50 | 3 | 3.2492e-03 | 5'UTR | 516912-517085 | 29693.m002027 | |  |
| 3231.snp | 29780 | 41166 | A | T | 83 | 8 | 64 | 4 | 4.9879e-04 | 5'UTR | 41123-41245 | 29780.m001319 | |  |
| 7621.snp | 29827 | 647152 | A | T | 21 | 0 | 15 | 1 | 9.6161e-03 | 3'UTR | 647079-647450 | 29827.m002644 | |  |
| 7476.snp | 29900 | 329883 | A | T | 34 | 1 | 70 | 5 | 1.1956e-06 | 5'UTR | 329427-329911 | 29900.m001589 | |  |
| 2023.snp | 29917 | 544581 | C | A | 13 | 0 | 25 | 2 | 4.7916e-02 | 3'UTR | 544217-544757 | 29917.m002006 | |  |
| 5386.snp | 29968 | 109001 | G | A | 54 | 0 | 21 | 0 | 1.9697e-02 | 3'UTR | 108937-109281 | 29968.m000648 | |  |
| 1008.snp | 29977 | 49190 | A | G | 26 | 0 | 36 | 2 | 5.6209e-03 | 3'UTR | 49142-49691 | 29977.m000253 | |  |
| 659.snp | 30025 | 76692 | A | G | 37 | 1 | 32 | 1 | 2.0723e-03 | 3'UTR | 76482-76786 | 30025.m000580 | |  |
| 212.snp | 30068 | 896517 | C | T | 10867 | 105 | 9500 | 140 | 0 | 5'UTR | 896427-896553 | 30068.m002651 | |  |
| 1052.snp | 30072 | 285329 | C | T | 36 | 0 | 46 | 3 | 9.6049e-05 | 3'UTR | 285252-285462 | 30072.m000969 | |  |
| 4476.snp | 30125 | 646434 | T | C | 73 | 2 | 118 | 5 | 1.3396e-03 | 5'UTR | 646279-646466 | 30125.m001254 | |  |
| 545.snp | 30128 | 2E+06 | A | G | 309 | 6 | 303 | 8 | 1.6816e-10 | 5'UTR | 1582515-1582604 | 30128.m008897 | |  |
| 4314.snp | 30143 | 167462 | A | T | 61 | 5 | 92 | 8 | 1.8540e-09 | 3'UTR | 166936-167736 | 30143.m001180 | |  |
| 6516.snp | 30152 | 874442 | G | A | 27 | 1 | 17 | 0 | 2.4306e-02 | 3'UTR | 874387-874675 | 30152.m002403 | |  |
| 7067.snp | 30190 | 3E+06 | C | T | 48 | 2 | 34 | 3 | 2.0439e-05 | 3'UTR | 2773056-2773446 | 30190.m011245 | |  |
| 472.snp | 29673 | 326477 | T | C | 16 | 0 | 17 | 0 | 2.4403e-02 | 5'UTR | 326352-326550 | 29673.m000944 | |  |
| 3805.snp | 29682 | 218125 | C | T | 11 | 0 | 114 | 2 | 6.4393e-03 | intergenic region | 217908-218258 | | |  |
| 1286.snp | 29624 | 327206 | G | A | 26 | 0 | 50 | 9 | 2.9441e-04 | intergenic region | 326907-327453 | | |  |
| 1584.snp | 29669 | 73220 | A | C | 61 | 0 | 25 | 1 | 4.7974e-02 | intergenic region | 72991-73360 | |  | |
| 7627.snp | 29827 | 681534 | G | A | 78 | 0 | 281 | 9 | 1.9347e-20 | Intergenic region | 681189-682133 | | |  |
| 7626.snp | 29827 | 680767 | A | T | 12 | 0 | 109 | 2 | 1.7879e-03 | Intergenic region | 680606-680967 | | |  |
| 1234.snp | 29905 | 23505 | G | T | 78 | 0 | 72 | 0 | 7.1550e-03 | Intergenic region | 23282-23582 | |  | |
| 7036.snp | 30190 | 1E+06 | T | G | 53 | 0 | 25 | 0 | 3.1877e-03 | Intergenic region | 1293623-1294014 | | |  |
| 3431.snp | 30205 | 226828 | G | A | 65 | 0 | 38 | 0 | 1.4097e-04 | intergenic region | 225966-226959 | | |  |
