# Supplementary material for: Genomic imprinting, methylation and parent-of-origin effects in reciprocal hybrid endosperm of castor bean
Source: Nucleic Acids Res. 2014 May 5;42(11):6987–98. doi: 10.1093/nar/gku375 (PMC4066788; doi:10.1093/nar/gku375)
Supplement: SUPPLEMENTARY DATA [file supp_gku375_nar-00469-v-2014-File009.zip › Supplementary_Table_S8.docx]

**Supplementary Table S8.** Genes with non-imprinted allele-specific expression pattern (>90% paternal reads in both hybrid endosperms). m, Maternal alleles; p, Paternal alleles.

| **SNP No.** | **Scaffold** | **Position** | **SNP_ZB107** | **SNP_ZB306** | **ZB107×ZB306** | | **ZB306×ZB107** | |  | **Gene ID** | **Annotation** |
| --- | --- | --- | --- | --- | --- | --- | --- | --- | --- | --- | --- |
|  |  |  |  |  | **m_ZB107** | **p_ZB306** | **m_ZB306** | **p_ZB107** | **FDR** |  |  |
| **ZB107 allele** |  |  |  |  |  |  |  |  |  |  |  |
| 1858.snp | 28597 | 51300 | T | G | 90 | 0 | 0 | 120 | 2.3067e-02 | 28597.m000122 | N-acetyltransferase, putative |
| 4787.snp | 30027 | 370350 | C | T | 17 | 0 | 0 | 30 | 1.3030e-04 | 30027.m000848 | conserved hypothetical protein |
| 6083.snp | 30101 | 99455 | A | G | 22 | 0 | 0 | 6 | 1.7925e-03 | 30101.m000376 | conserved hypothetical protein |
| 5083.snp | 28492 | 22423 | C | T | 125 | 0 | 0 | 70 | 4.8392e-04 | 28492.m000467 | electron carrier, putative |
| 3317.snp | 29904 | 69461 | C | T | 35 | 0 | 0 | 36 | 6.5313e-10 | 29904.m002897 | conserved hypothetical protein |
| 1950.snp | 30114 | 166147 | C | T | 36 | 0 | 0 | 56 | 1.7363e-08 | 30114.m000526 | serine carboxypeptidase, putative |
| **ZB306 allele** | **Scaffold** | **Position** | **SNP_ZB107** | **SNP_ZB306** | **m_ZB107** | **p_ZB306** | **m_ZB306** | **p_ZB107** | **FDR** | **Gene ID** | **Annotation** |
| 5724.snp | 30147 | 1973377 | T | C | 3 | 143 | 138 | 2 | 1.7268e-35 | 30147.m014437 | proteasome subunit alpha type |
| 1027.snp | 29785 | 484216 | T | G | 1 | 106 | 89 | 1 | 2.4071e-23 | 29785.m000971 | tubulin beta chain |
| 1810.snp | 29726 | 1108438 | G | A | 0 | 73 | 33 | 0 | 2.5854e-09 | 29726.m004084 | f3e22.6 protein |
| 3829.snp | 29629 | 36636 | C | A | 1387 | 10063 | 9509 | 1457 | 0 | 29629.m001355 | 11S globulin subunit beta precursor |
